# BMI1 Overexpression Alleviates Metabolic Syndrome‐Related Liver Injury by Inhibiting the Activation of the METTL3/AHNAK Axis

**DOI:** 10.1002/kjm2.70308

**Published:** 2026-10-26

**Authors:** Yun‐Jiang Chen, Bei Song, Kai‐Yu Cai, Yue‐Ling Du, Yan‐Chun Gong

**Affiliations:** ^1^ Department of General Medicine Ruijin Hospital, Shanghai Jiao Tong University School of Medicine Shanghai People's Republic of China

**Keywords:** AHNAK, BMI1, liver injury, metabolic syndrome, METTL3

## Abstract

Abnormal protein levels have been demonstrated to contribute to the progression of various diseases, including metabolic syndrome (MetS). Here, we speculated that the downregulation of BMI1 might lead to the failure of METTL3 degradation and contribute to METTL3 overexpression in MetS‐related liver injury. Rats were fed a diet consisting of a high proportion of fat and fructose (HFHF), and genetic intervention was performed via tail vein injection of liver‐specific AAV vectors. In the liver tissue of rats induced by HFHF, BMI1 was downregulated, while METTL3 and AHNAK were upregulated. BMI1 overexpression suppressed HOMA‐IR levels, downregulated the serum levels of TC, TG, ALT, and AST, and reduced oxidative stress‐related markers and the number of lipid droplets in liver tissues in rats with HFHF. BMI1 overexpression promoted the ubiquitination and degradation of METTL3 in BRL 3A cells, while METTL3 overexpression enhanced the m6A modification of AHNAK, leading to upregulation of AHNAK. Overexpression of METTL3 or AHNAK attenuated the mitigating effect of BMI1 overexpression in vivo and in vitro by activating the TGF‐β/SMAD signaling. These findings suggest that BMI1 overexpression alleviates MetS‐associated liver injury by inhibiting AHNAK m6A methylation through promoting METTL3 degradation, thereby suppressing the TGF‐β/SMAD signaling.

## 1 Introduction

Metabolic syndrome (MetS) is a constellation of metabolic dysregulations, including insulin resistance, atherogenic dyslipidemia, central obesity, and hypertension [1, 2]. A higher prevalence of MetS has been reported in an urban community in China, and the prevalence was 33.9% among individuals aged ≥ 32 years, 32.5% among women, and 35.1% among men [3]. MetS is characterized by a series of lipid disorders, and nonalcoholic fatty liver disease and diabetic kidney disease are considered to be the common hepatic and renal manifestations of MetS following abnormal lipid metabolism [4]. Moreover, individuals diagnosed with MetS face an increased probability of developing damage to vital organs within multiple bodily systems [5]. Therefore, more efforts are needed to elucidate the molecular mechanisms underlying liver injury to improve the prognosis of these patients.

N6‐methyladenosine (m6A) is the most prevalent post‐transcriptional modification in eukaryotic RNA and plays a regulatory role in gene expression through various mechanisms, including nuclear export, localization, translation efficiency, and mRNA stability [6]. The life cycle of m6A methylation comprises three distinct components: writers, erasers, and readers, which are responsible for the addition, removal, and recognition of m6A, respectively [7]. Our previous study demonstrated that the writer methyltransferase‐like 3 (METTL3) facilitated the m6A modification of HDAC1 mRNA, and the knockdown of METTL3 suppressed HDAC1 and augmented FGF21 expression, consequently ameliorating liver injury in rats with MetS [8]. However, the upstream modifier of METTL3 in MetS‐related liver injury remains to be elucidated.

Abnormal protein levels have been demonstrated to contribute to the progression of various diseases, including MetS [9]. For instance, it has been established that SUMOylation of METTL3 modulates its function [10]. Here, we identified multiple ubiquitination sites on METTL3 and identified B cell‐specific Moloney murine leukemia virus integration site 1 (BMI1) as a potential E3 ubiquitin ligase that interacts with METTL3. BMI1 (also known as PCGF4 or RNF51), a component of the polycomb repressive complex 1, has been identified as a polycomb protein that plays a crucial role in preventing premature senescence and maintaining the self‐renewal capacity of stem cells [11]. The RING domain of BMI1 is also positioned on the unmodified nucleosome side, enabling the recruitment of the E2 enzyme to ubiquitinate H2AK119 within the same nucleosome or across nucleosomes [12]. As resveratrol attenuates high‐fat diet (HFD)‐induced hepatic lipotoxicity by upregulating BMI1 [13], we hypothesized that downregulation of BMI induced by HFD might contribute to the failure of METTL3 degradation, thereby aggravating MetS‐induced liver injury.

## 2 Materials and Methods

### 2.1 Cells and Lentiviruses

Rat hepatocyte BRL 3A cells (CL‐0036) were procured from Procell (Wuhan, Hubei, China) and cultured in BRL 3A cell complete medium (CM‐0036, Procell). The medium was changed twice weekly, and cells were maintained at 37°C in a 5% CO_2_ environment. The cell line used in this experiment was negative for mycoplasma and was identified via STR analysis.

BRL 3A cells were infected with lentiviruses overexpressing BMI1, METTL3, AHNAK, or a control vector in the presence of 8 μg/mL polystyrene and selected for 1 week using 1 μg/mL puromycin or 300 μg/mL neomycin.

### 2.2 Quantitative Polymerase Chain Reaction (qPCR)

Total RNA was isolated from BRL 3A cells using the TransZol Up Plus RNA Kit (ER501‐01‐V2, Transgen, Beijing, China) and reverse transcribed into cDNA using the PrimeScript RT Reagent Kit with gDNA Eraser (RR047A, Takara Holdings Inc., Kyoto, Japan). qPCR was conducted using TB Green Premix Ex Taq II (Tli RNaseH Plus) (RR820A, Takara) on the 7500 Fast Real‐Time Fluorescent Quantitative PCR System (4351107, Thermo Fisher Scientific Inc., Waltham, MA, USA). The specific sequences of the primers used were as follows: rat BMI1: forward primer 5′‐GCTGGATGCCAAGTGGTCT‐3′; reverse primer 5′‐GTGAGGGAACTGTGGGTGAG‐3′; rat AHNAK: forward primer 5′‐GCAGGGAGAGGAACCATGTC‐3′; reverse primer 5′‐CGGTCGAGTCTACCAACACC‐3′; rat β‐actin: forward primer 5′‐CCCGCGAGTACAACCTTCTT‐3′; reverse primer 5′‐AACACAGCCTGGATGGCTAC‐3′. The relative mRNA expression levels were calculated using the 2^−ΔΔCT^ method.

### 2.3 Cycloheximide (CHX) Chase Assay

BRL 3A cells infected with lentiviruses were treated with 100 μg/mL CHX (purity: ≥ 98%, MS0035‐1000MG, Maokang, Shanghai, China) and collected at designated time points (0, 3, 6, and 9 h) for subsequent Western blot analysis.

### 2.4 Ubiquitination Assays

BRL 3A cells infected with lentivirus were treated with 10 μM MG132 (Purity: 99.90%, HY‐13259, MedChemExpress, Monmouth Junction, NJ, USA) for 12 h. BRL 3A cells were lysed using IP lysis buffer and incubated overnight at 4°C with anti‐METTL3 antibody (1:50, 86132, Cell Signaling Technologies, Beverly, MA, USA). The immune complexes were purified using protein A‐G agarose and boiled for 10 min in SDS‐PAGE loading buffer before undergoing Western blot analysis.

### 2.5 Experimental Animals

The animal protocol was approved by the Committee on the Ethics of Animal Experiments of the Shanghai Jiao Tong University School of Medicine. Six‐week‐old male SD rats (200–250 g) were procured from SJA Laboratory Animal Co. Ltd. (Changsha, Hunan, China). All rats were housed in a thermostatically controlled environment with a 12‐h light/dark cycle.

After 1 week of adaptive feeding, rat modeling was performed based on our previous research [8]. Rats were fed an HFD (composition: 59% fat, 21% protein, 20% carbohydrates) with 15% fructose added to their drinking water. Rats in the normal group were fed a normal diet (ND, composition: 16% fat, 22% protein, 62% carbohydrates) and provided with normal drinking water. During the first week of HFD feeding, 100 μL of an adeno‐associated virus (AAV) vector (Hanbio, Shanghai, China) driven by the liver‐specific TBG promoter was administered via tail vein injection; the serotype was AAV8, and the viral titer was 1 × 10^13^ GC/mL. Overexpression (OE) AAVs included AAV‐BMI1, which contained the BMI1 (NM_001107368.3) CDS sequence; AAV‐METTL3, which contained the METTL3 (NM_001024794.1) CDS sequence; AAV‐AHNAK, which contained the AHNAK (NM_001398672.1) CDS sequence; and the control empty vector AAV‐NC. Systolic blood pressure and body weight were measured every 4 weeks. After 12 weeks of feeding, serum was collected via suborbital blood sampling, and fasting blood glucose levels were assessed in the rats. Rats were euthanized with 200 mg/kg sodium pentobarbital. Rat liver tissues were collected to prepare paraffin or frozen sections for histological staining or were stored frozen for subsequent testing.

### 2.6 Western Blot

Total protein was extracted from BRL 3A cells and rat liver tissue using RIPA buffer (R0010, Solarbio, Beijing, China). The Nuclear and Cytoplasmic Protein Extraction Kit (P0028, Beyotime, Shanghai, China) was used to extract total protein from different cellular fractions. The protein concentration was quantified using the Pierce BCA Protein Assay Kit (23225, Thermo Fisher). The extracted proteins were separated using SDS‐PAGE and transferred onto PVDF membranes. After blocking with 5% BSA for 1.5 h, the membranes were incubated with primary antibodies against BMI1 (1:2000, PA5‐87371, Thermo Fisher), METTL3 (1:1000, 86132, Cell Signaling Technologies), Ubiquitin (Ub, 1:1000, ab134953, Abcam, Cambridge, MA, USA), TGF‐β1 (1:1000, ab215715, Abcam), p‐SMAD2 (1:500, AP0269, ABclonal, Wuhan, Hubei, China), p‐SMAD3 (1:1000, AP0727, ABclonal), SMAD2 (1:1000, A19114, ABclonal), SMAD3 (1:1000, A16913, ABclonal), Lamin B1 (1:1000, ab16048, Abcam), β‐actin (1:1000, ab8227, Abcam), and ɑ‐tubulin (1:100, PA5‐19489, Thermo Fisher) at 4°C overnight. On the following day, the membranes were incubated with HRP‐conjugated goat anti‐rabbit antibody (1:5000, G‐21234, Thermo Fisher) at room temperature for 1 h. Finally, the membranes were soaked in ECL Western Blotting Substrate (PE0010, Solarbio). The protein levels were quantified using ImageJ software. β‐actin or ɑ‐tubulin was used as the loading control. Please refer to Supplementary Material 1 for independent biological replicates of liver samples from six rats per group in the animal experiments.

### 2.7 Co‐Immunoprecipitation (Co‐IP)

Immunoprecipitation Kit with Protein A + G Magnetic Beads (P2179S, Beyotime) was used to validate the interaction between BMI1 and METTL3. RL 3A cells or rat liver tissues were lysed using a pre‐configured lysis buffer containing an inhibitor. The lysate was incubated with anti‐BMI1 (1:25, PA5‐87371, Thermo Fisher) or IgG (1:50, ab172730, Abcam) on Protein A + G magnetic beads by rotation. The magnetic beads were incubated with the lysate overnight at 4°C and eluted, followed by Western blot analysis.

### 2.8 Homeostatic Model Assessment of Insulin Resistance (HOMA‐IR)

Fasting insulin and blood glucose levels were measured in rats. HOMA‐IR = [fasting insulin (μU/mL) × fasting blood glucose (mmol/L)]/22.5 [14].

### 2.9 Serum and Liver Biochemical Parameter Tests

The levels of oxidative stress markers in rat liver tissue were assessed using the lipid peroxidation (MDA) assay kit (ab118970, Abcam), superoxide dismutase (SOD) activity assay kit (ab65354, Abcam), and reduced glutathione (GSH) assay kit (A006‐1‐1, Nanjing JianCheng, Nanjing, Jiangsu, China). The alanine aminotransferase (ALT) assay kit (C009‐2‐1, JianCheng, Nanjing, Jiangsu, China) and the aspartate aminotransferase (AST) assay kit (C010‐2‐1, JianCheng) were used to measure the levels of liver damage markers in the serum. The total cholesterol (TC) assay kit (A111‐1‐1, JianCheng) and the triglyceride (TG) assay kit (A110‐1‐1, JianCheng) were used to measure lipid levels in serum and rat liver tissues.

### 2.10 ELISA

TGF‐β1 levels in BRL 3A cell culture supernatant and rat serum samples were assessed using the TGF‐β1 ELISA Kit (ml002856, MLBio, Shanghai, China). TGF‐β1 standards of various concentrations and the test samples were incubated with a working solution of biotinylated antibodies at 37°C in the dark for 1 h and with a 1× SA‐HRP working solution for 30 min. After the addition of the color development solution, the absorbance was measured at 450 nm using a microplate reader, and the TGF‐β1 concentration in the test samples was calculated based on the standard curve.

### 2.11 Hematoxylin–Eosin (HE) Staining

HE staining (MM1013‐200ML, Maokang) was performed on rat liver tissue paraffin sections. After dewaxing and rehydration, the rat liver paraffin‐embedded sections were sequentially placed in hematoxylin staining solution, blue staining solution, and eosin staining solution. Following incubation, the sections were dehydrated again using a gradient ethanol series, cleared with xylene, sealed, and observed under an optical microscope. The severity of liver damage in rats was assessed by pathologists unaware of the grouping using a semi‐quantitative scoring system, categorized into five grades based on damage severity: Grade 1, mild damage (< 1%); Grade 2, slight damage (1%–25%); Grade 3, moderate damage (26%–50%); Grade 4: moderate/severe injury (51%–75%); and Grade 5: severe injury (76%–100%).

### 2.12 Oil Red O Staining

Frozen sections of rat liver tissue were stained using the Modified Oil Red O Stain Kit (G1261, Solabio). The sections were fixed in 10% formalin for 30 min, then immersed in 60% isopropanol for 20 s, followed by immersion in modified Oil Red O staining solution for 10 min. Hematoxylin staining solution was added dropwise for counterstaining. After mounting with glycerol gelatin, the slides were observed under an optical microscope.

### 2.13 RNA Stability Assay

BRL 3A cells infected with OE‐NC or OE‐METTL3 lentivirus were treated with 2 mg/mL actinomycin D (purity: 99.74%, S8964, Selleck, Shanghai, China). Cells were harvested at 0, 2, 4, 8, 16, and 24 h post‐infection for RT‐qPCR analysis.

### 2.14 RIP

The Magna RIP RNA‐Binding Protein Immunoprecipitation Kit (17‐700, Sigma‐Aldrich Chemical Company, St Louis, MO, USA) was used. In short, total RNA was isolated from treated BRL 3A cells. The extracted RNA was fragmented into 100‐nucleotide‐sized fragments using RNA fragmentation buffer and incubated with magnetic beads‐conjugated anti‐m6A (1:50, A19841, ABclonal), anti‐METTL3 (1:10, 86132, Cell Signaling Technologies), or IgG (1:50, ab172730, Abcam) at 4°C for 2 h. Subsequently, elution buffer was added, and the eluted and purified RNA fragments were analyzed by RT‐qPCR.

### 2.15 Fluorescent in Situ Hybridization (FISH)

The FISH kit (Bes1001, BersinBio, Guangzhou, Guangdong, China) was used to visualize the subcellular localization of AHNAK mRNA. The BRL 3A cells were fixed with 4% paraformaldehyde, followed by permeabilization, dehydration, and other processing steps. The cells were incubated with pre‐incubation buffer at 37°C for 30 min to reduce nonspecific binding, denatured with the hybridization buffer containing the AHNAK mRNA probe at 73°C for 5 min, and quickly transferred to 37°C for overnight hybridization. After labeling the cell nuclei with DAPI, the samples were observed under a fluorescence microscope, and the fluorescence intensity in the nuclei and cytoplasm was measured separately using ImageJ software (v1.54g).

### 2.16 Statistical Analyses

Statistical analysis was performed using Prism 10.3.1 (GraphPad, San Diego, CA, USA). The results are expressed as the means ± SEM. In the context of in vivo experiments, n denotes the number of rats allocated to each group, and in vitro experiments utilized n to represent the number of independent biological replicates. An unpaired *t*‐test was used to compare two variables, and analysis of variance (ANOVA) was used to compare three or more variables, followed by Šídák's or Tukey's post hoc test. The level of statistical significance was set at *p* < 0.05.

## 3 Results

### 3.1 Overexpression of BMI1 Promotes Ubiquitination Modification of METTL3 in BRL 3A Cells

In the GPS‐Uber database (https://gpsuber.biocuckoo.cn/index.php), we predicted multiple ubiquitination sites on the METTL3 protein (Figure 1A). E3 ubiquitin ligases with METTL3 as the substrate were predicted in the Ubibrowser 2.0 database (http://ubibrowser.bio‐it.cn/ubibrowser_v3/) (Figure 1B). Based on confidence score ranking, we found that BMI1 had the highest confidence score (0.821) among the E3 ubiquitin ligases. Furthermore, BMI1 was predicted to contain a recognition motif within the protein sequence domain of METTL3 (Figure 1C).

**FIGURE 1:**
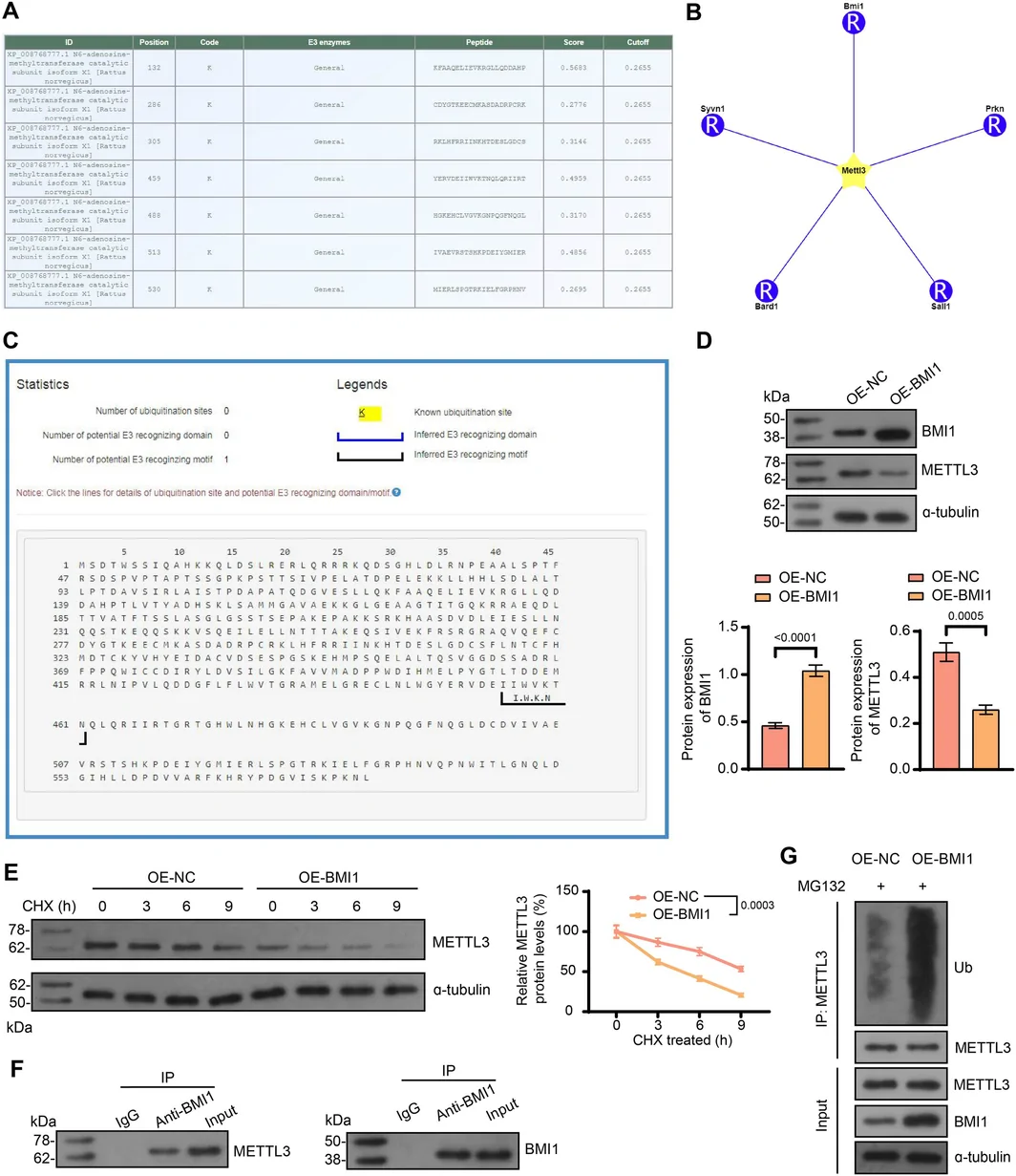
Overexpression of BMI1 promotes ubiquitination modification of METTL3. (A) Predicting ubiquitination sites on the METTL3 protein using the GPS‐Uber database. (B) Predicting E3 ubiquitin ligases with METTL3 as the substrate in the Ubibrowser 2.0 database. (C) BMI1 recognition motif within the protein sequence domain of METTL3. (D) Protein expression of both BMI1 and METTL3 in BRL 3A cells upon overexpression of BMI1 was examined using western blot analysis. (E) METTL3 protein stability in BRL 3A cells upon overexpression of BMI1 and CHX treatment was examined using western blot analysis. (F) The interaction between BMI1 and METTL3 in BRL 3A cells was examined using Co‐IP. (G) The ubiquitination level of METTL3 in BRL 3A cells following BMI1 overexpression was examined using a ubiquitination assay. Data are presented as the mean ± SEM (*n* = 5), and statistical significance was assessed using an unpaired *t*‐test or two‐way ANOVA.

First, to elucidate the relationship between BMI1 and METTL3, we overexpressed BMI1 in rat hepatocyte BRL 3A cells. We found that BMI1 protein expression was significantly elevated, whereas METTL3 protein expression was reduced following OE‐BMI1 infection (Figure 1D). We treated BRL 3A cells with CHX, and overexpression of BMI1 reduced the stability of the METTL3 protein (Figure 1E). Co‐IP experiments confirmed the protein–protein interaction between BMI1 and METTL3 in BRL 3A cells (Figure 1F). In ubiquitination assays, we found that elevated BMI1 expression significantly increased METTL3 ubiquitination levels (Figure 1G).

### 3.2 BMI1 Is Downregulated in Rats With MetS, and Its Overexpression Alleviates Liver Injury in Rats With MetS

A 12‐week HFHF diet was administered to rats to establish a MetS rat model, followed by genetic intervention involving BMI1 overexpression (OE‐BMI1) using AAV vectors. Reduced BMI1 expression was identified in the liver tissue of HFHF‐induced rats. However, its expression was significantly increased in liver tissues following OE‐BMI1 administration (Figure 2A). Co‐IP experiments revealed that the interaction between BMI1 and METTL3 in liver tissues was attenuated in HFHF‐induced rats, whereas BMI1 overexpression restored their interaction (Figure 2B). Starting from feeding a HFHF diet, rats were weighed, and their systolic blood pressure was measured every 4 weeks. Overexpression of BMI1 limited the gradual weight gain and blood pressure elevation induced by the HFHF diet in rats (Figure 2C,D). Additionally, upregulation of BMI1 expression suppressed HFHF‐induced hyperglycemia and HOMA‐IR in rats (Figure 2E,F). Overexpression of BMI1 also led to reduced serum TC, TG, ALT, and AST levels in HFHF rats (Figure 2G). Biochemical analysis revealed that in the HFHF rat liver tissues, the levels of MDA, TC, and TG were elevated, whereas SOD and GSH levels were reduced. BMI overexpression downregulated MDA, TC, and TG levels and upregulated SOD and GSH levels in the liver tissues of rats (Figure 2H). As shown in Figure 2I, BMI1 overexpression significantly ameliorated the increased inflammatory cell infiltration in rat livers induced by HFHF. Oil Red O staining revealed a significant reduction in the number of red lipid droplets in the liver tissue of HFHF‐induced rats following BMI1 overexpression (Figure 2J).

**FIGURE 2:**
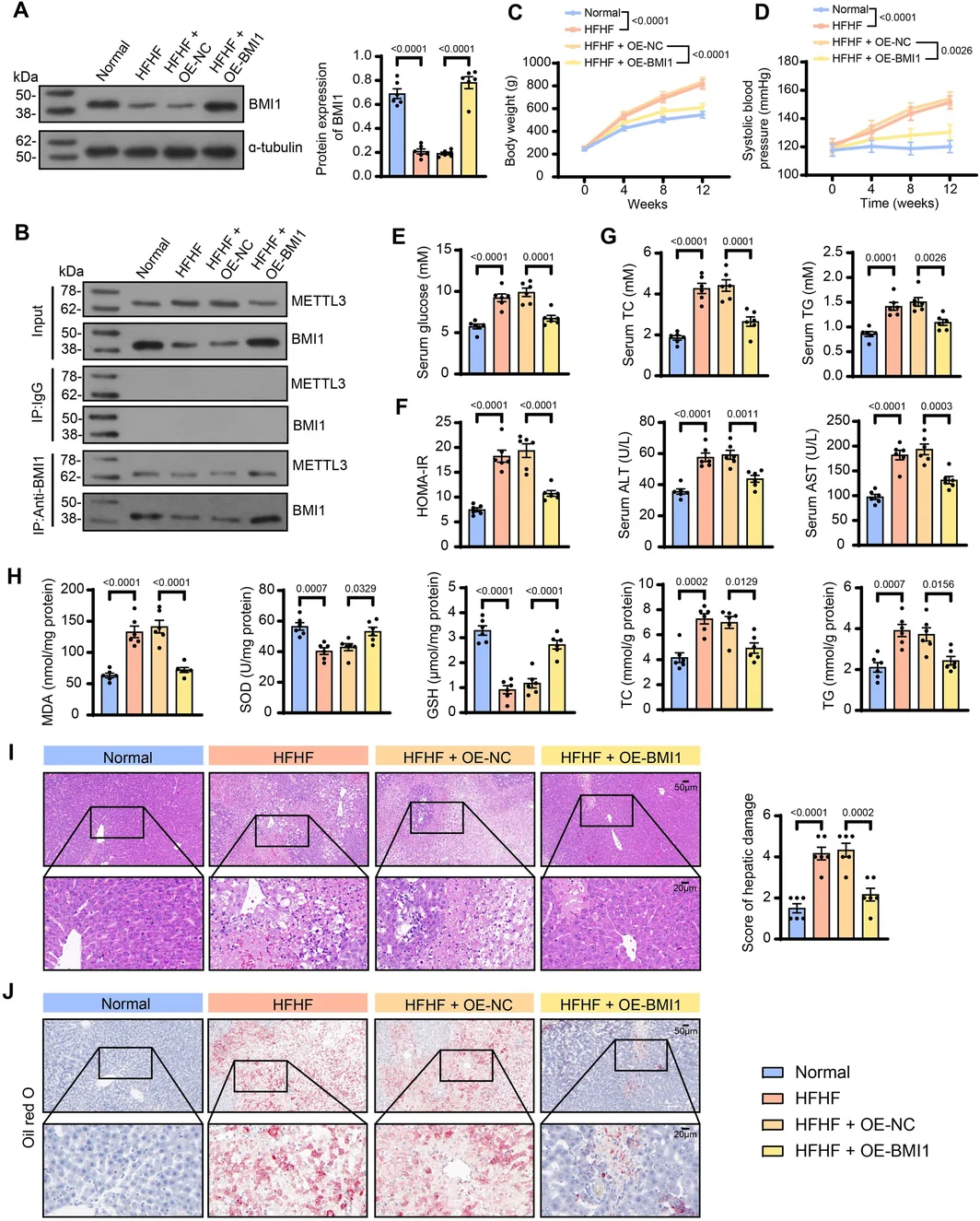
Overexpression of BMI1 alleviates MetS and liver injury in rats induced by HFHF. (A) BMI1 protein expression in liver tissues from rats induced with HFHF or pre‐administered with AAV vectors harboring OE‐BMI1 was examined using Western blot analysis. (B) The interaction between BMI1 and METTL3 in rat liver tissues was examined using Co‐IP. (C) The body weight of rats was measured every 4 weeks. (D) The systolic blood pressure of rats was measured every 4 weeks. The blood glucose levels (E) and HOMA‐IR (F) in rats were measured at the endpoint. (G) Detection of TC, TG, ALT, and AST levels in rat serum. (H) Biochemical analysis of MDA, SOD, GSH, TC, and TG levels in rat liver tissues. (I) Representative HE images of rat liver tissues and liver damage scores. (J) Representative Oil red O images of rat liver tissues. Data are presented as the means ± SEM (*n* = 6), and statistical significance was assessed using one‐way or two‐way ANOVA.

### 3.3 Overexpression of METTL3 Attenuates the Effect of BMI1 Overexpression Against Liver Injury in Rats With MetS

Following the overexpression of BMI1 in HFHF rats, combined gene intervention therapy with METTL3 overexpression was administered. Western blot analysis detected the upregulation of METTL3 expression in the liver tissue of HFHF‐induced rats, which was downregulated by overexpression of BMI1. However, ectopic expression of METTL3 increased METTL3 expression in the liver tissue of rats overexpressing BMI1 (Figure 3A). Combined overexpression of METTL3 further led to a gradual increase in body weight and blood pressure in HFHF‐induced rats (Figure 3B,C). Similarly, overexpression of METTL3 also increased blood glucose levels and HOMA‐IR in rats fed with HFHF (Figure 3D,E). The liver injury markers in the serum (Figure 3F) and oxidative stress markers and lipid levels (Figure 3G) in the liver were all enhanced when METTL3 was overexpressed in the liver. HE staining revealed that combined overexpression of METTL3 attenuated the anti‐inflammatory effect of BMI1 overexpression on the liver in HFHF‐induced rats (Figure 3H). An increased number of red lipid droplets was found in the liver tissues of METTL3‐overexpressing rats (Figure 3I).

**FIGURE 3:**
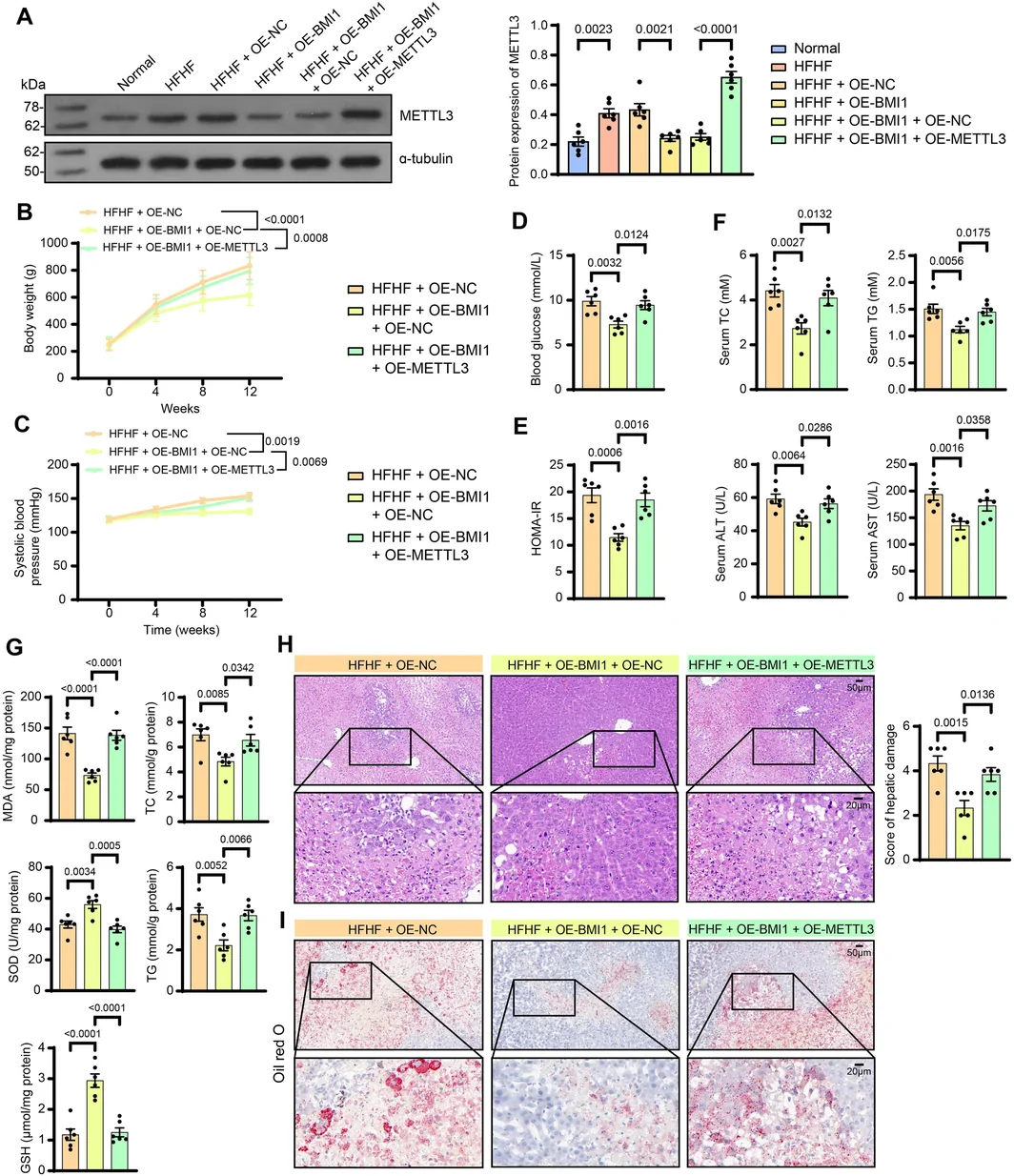
Overexpression of METTL3 attenuates the protective effect of BMI1 overexpression against liver injury in rats with MetS. The HFHF + OE‐NC group was the same as the HFHF + OE‐NC group shown in Figure 2, and all other groups were drawn from independent animal populations. (A) METTL3 protein expression in liver tissues from rats induced with HFHF or pre‐administered with AAV vectors harboring OE‐BMI1 or OE‐BMI1 + OE‐METTL3 was examined using western blot analysis. (B) Body weight of rats was measured every 4 weeks. (C) Systolic blood pressure of rats was measured every 4 weeks. Blood glucose levels (D) and HOMA‐IR (E) in rats were measured at the endpoint. (F) Detection of TC, TG, ALT, and AST levels in rat serum. (G) Biochemical analysis of MDA, SOD, GSH, TC, and TG levels in rat liver tissues. (H) Representative HE images of rat liver tissues and liver damage scores. (I) Representative Oil red O images of rat liver tissues. Data are presented as the means ± SEM (*n* = 6), and statistical significance was assessed using one‐way/two‐way ANOVA.

### 3.4 Overexpression of METTL3 Promotes m6A Modification of AHNAK mRNA

To investigate the novel functional targets of METTL3 in MetS, we analyzed its targets as a writer in the RM2Target database (http://rm2target.canceromics.org/#/home). Red genes represent validated targets, green genes indicate predicted binding with METTL3, and yellow genes indicate potential expression changes associated with METTL3 perturbation (Figure 4A). Using the GEPIA database (http://gepia.cancer‐pku.cn/index.html), we predicted a significant positive correlation between METTL3 and AHNAK expression in liver tissues (Figure 4B).

**FIGURE 4:**
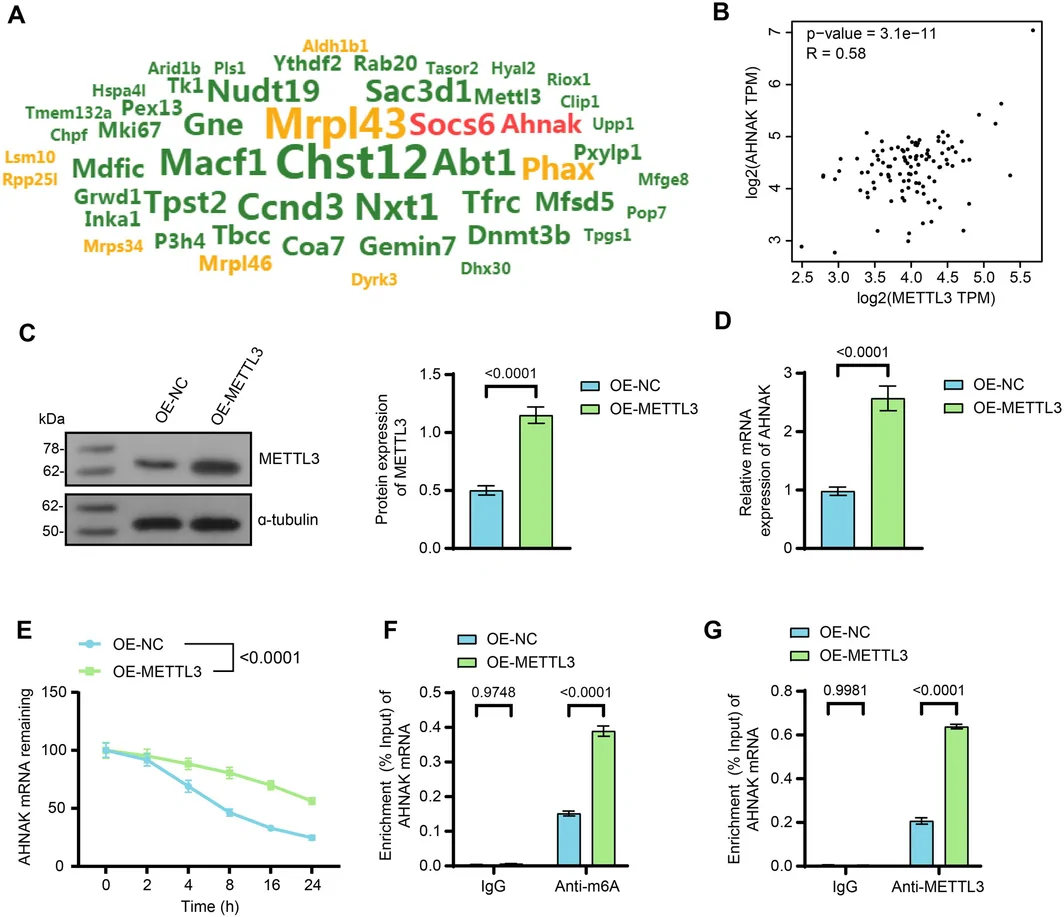
Overexpression of METTL3 promotes m6A modification of AHNAK mRNA. (A) Targets of METTL3 in the RM2Target database. (B) The correlation between METTL3 and AHNAK expression in liver tissues was predicted using the GEPIA database. (C) METTL3 protein expression in BRL 3A cells after infection with OE‐METTL3 was examined using western blot analysis. (D) AHNAK mRNA expression in BRL 3A cells after infection with OE‐METTL3 was examined using RT‐qPCR. (E) AHNAK mRNA stability in BRL 3A cells after infection with OE‐METTL3 was examined using RT‐qPCR. (F) m6A modification levels on AHNAK mRNA in BRL 3A cells following METTL3 overexpression were examined using m6A‐RIP‐qPCR. (G) The enrichment level of anti‐METTL3 on AHNAK mRNA in BRL 3A cells following METTL3 overexpression was examined using RIP‐qPCR. Data are presented as the means ± SEM (*n* = 5), and statistical significance was assessed using an unpaired *t*‐test or two‐way ANOVA.

BRL 3A cells were infected with METTL3‐overexpressing lentivirus (OE‐METTL3). Western blot analysis revealed a significant increase in METTL3 protein expression after OE‐METTL3 infection (Figure 4C). METTL3 overexpression promoted AHNAK mRNA expression (Figure 4D). FISH analysis revealed that METTL3 overexpression increased AHNAK mRNA expression in the cytoplasm, whereas AHNAK expression levels in the nucleus remained largely unchanged (Figure S1A). In addition, the AHNAK protein was exclusively expressed in the cytoplasm, and its expression in the cytoplasm increased following METTL3 overexpression (Figure S1B). The Human Protein Atlas project (https://www.proteinatlas.org/) also showed that AHNAK protein in the liver was exclusively expressed in the cytoplasm (Figure S1C). Actinomycin D assays indicated that METTL3 overexpression increased the stability of AHNAK mRNA in BRL 3A cells (Figure 4E). METTL3 overexpression not only increased m6A modification levels on AHNAK mRNA (Figure 4F) but also augmented the enrichment of AHNAK mRNA by anti‐METTL3 (Figure 4G).

### 3.5 BMI1 Mediates the Activation of the TGF‐β/SMAD Signaling Through the METTL3/AHNAK Axis

RT‐qPCR analysis revealed that BMI1 overexpression significantly reduced AHNAK mRNA expression, whereas combined METTL3 overexpression markedly increased AHNAK mRNA expression in BRL 3A cells (Figure 5A). Moreover, overexpression of BMI1 suppressed anti‐m6A and anti‐METTL3 on AHNAK mRNA, which was enhanced by the combined overexpression of METTL3 in BRL 3A cells (Figure 5B,C). Following BMI1 overexpression, the expression levels of AHNAK, TGF‐β1, p‐SMAD2, and p‐SMAD3 were significantly downregulated in BRL 3A cells. Concurrent OE‐AHNAK or OE‐METTL3 increased the expression levels of AHNAK, TGF‐β1, p‐SMAD2, and p‐SMAD3 in BRL 3A cells (Figure 5D). ELISA revealed that BMI1 overexpression inhibited TGF‐β1 release from BRL 3A cells, whereas the combined overexpression of AHNAK or METTL3 increased TGF‐β1 concentrations in the cell culture supernatant (Figure 5E).

**FIGURE 5:**
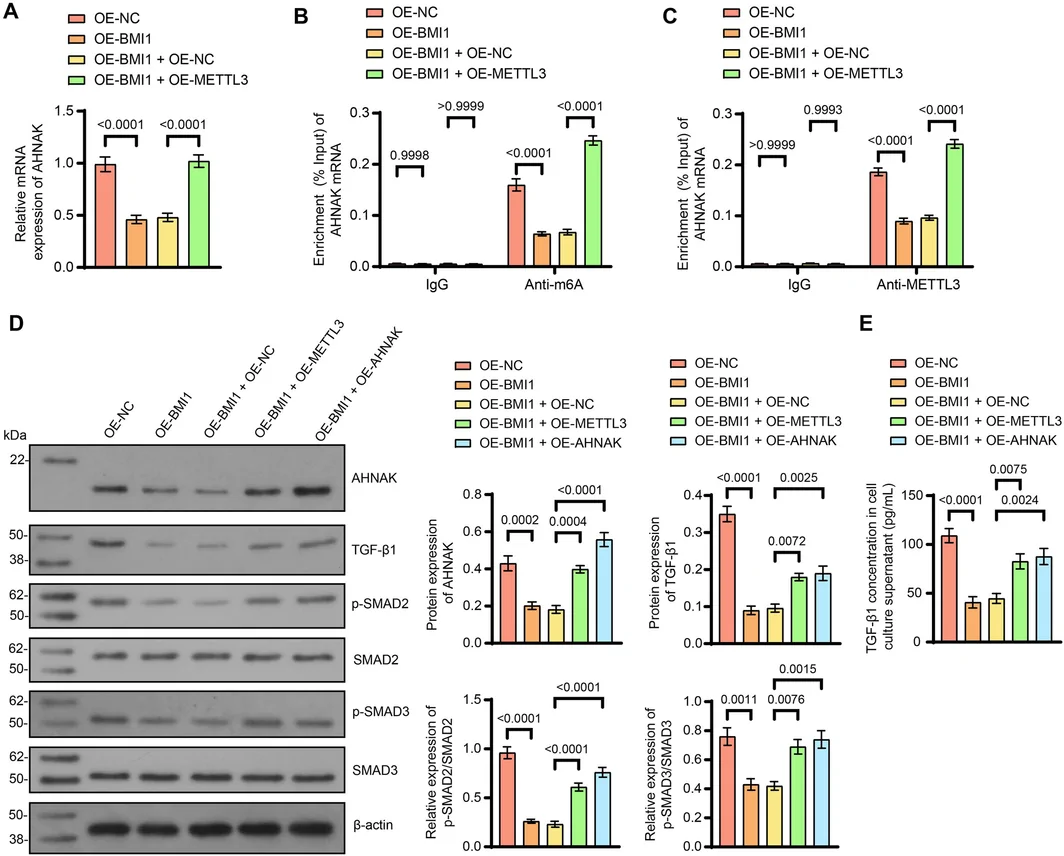
Overexpression of BMI1 impairs the TGF‐β/SMAD signaling through METTL3/AHNAK in BRL 3A cells. (A) AHNAK mRNA expression in BRL 3A cells after infection with OE‐BMI1 or OE‐BMI1 + OE‐METTL3 was detected using RT‐qPCR. (B) m6A modification levels on AHNAK mRNA in BRL 3A cells after infection with OE‐BMI1 or OE‐BMI1 + OE‐METTL3 were examined using m6A‐RIP‐qPCR. (C) The enrichment level of anti‐METTL3 on AHNAK mRNA in BRL 3A cells after infection with OE‐BMI1 or OE‐BMI1 + OE‐METTL3 was examined using RIP‐qPCR. (D) Protein expression of AHNAK, TGF‐β1, p‐SMAD2, p‐SMAD3, SMAD2, and SMAD3 in BRL 3A cells treated with overexpression of BMI1 and overexpression of either AHNAK or METTL3. (E) TGF‐β1 levels in cell culture supernatant were examined using ELISA. Data are presented as the means ± SEM (*n* = 5), and statistical significance was assessed using one‐way or two‐way ANOVA.

### 3.6 Overexpression of AHNAK Partially Overturns the Mitigating Effect of BMI1 Overexpression on Liver Injury in Rats

Rats overexpressing BMI1 were subjected to combined gene intervention using AAV vectors. Consistent with the in vitro findings, expression of AHNAK, TGF‐β, p‐SMAD2, and p‐SMAD3 was significantly elevated in the liver tissues of HFHF‐fed rats, which was reduced by overexpression of BMI1. Following combined gene interference treatment with OE‐METTL3 or OE‐AHNAK, the protein expression of AHNAK, TGF‐β, p‐SMAD2, and p‐SMAD3 was significantly elevated (Figure 6A). In addition, serum TGF‐β1 levels were significantly elevated in HFHF rats. Overexpression of BMI1 significantly reduced serum TGF‐β1 levels, whereas combined overexpression of METTL3 or AHNAK restored serum TGF‐β1 levels (Figure 6B).

**FIGURE 6:**
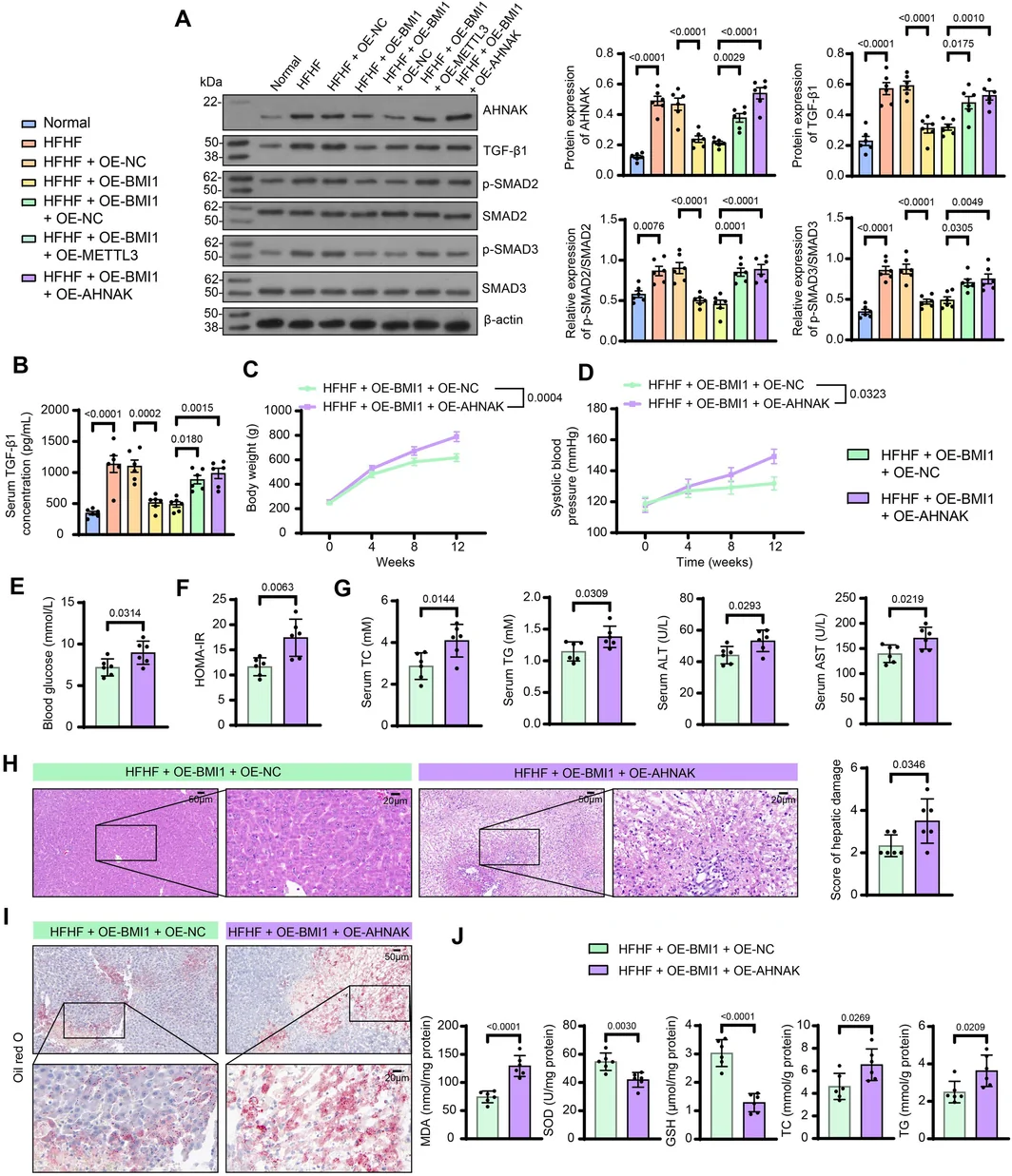
Overexpression of AHNAK partially reverses the ameliorative effect of BMI1 overexpression on liver injury in rats with MetS. Rats in all groups were drawn from separate animal populations. (A) Protein expression of AHNAK, TGF‐β1, p‐SMAD2, p‐SMAD3, SMAD2, and SMAD3 in liver tissues from rats treated with overexpression of BMI1 and overexpression of either AHNAK or METTL3. (B) TGF‐β1 concentrations in rat serum were examined using ELISA. (C) The body weight of rats was measured every 4 weeks. (D) The systolic blood pressure of rats was measured every 4 weeks. The blood glucose levels (E) and HOMA‐IR (F) in rats were measured at the endpoint. (G) Detection of TC, TG, ALT, and AST levels in rat serum. (H) Representative HE images of rat liver tissues and liver damage scores. (I) Representative Oil red O images of rat liver tissues. (J) Biochemical analysis of MDA, SOD, GSH, TC, and TG levels in rat liver tissues. Data are presented as the mean ± SEM (*n* = 6), and statistical significance was assessed using an unpaired *t*‐test or one‐way/two‐way ANOVA.

Combined overexpression of AHNAK in BMI1‐overexpressing HFHF rats resulted in progressive weight gain and elevated blood pressure (Figure 6C,D). Additionally, blood glucose levels increased, and HOMA‐IR was upregulated in rats with AHNAK overexpression (Figure 6E,F). The liver injury was again accentuated in rats overexpressing AHNAK, as evidenced by higher serum levels of TC, TG, ALT, and AST (Figure 6G), elevated HE scores (Figure 6H), and red lipid droplets (Figure 6I). Following OE‐AHNAK intervention, MDA, TC, and TG levels were increased, while SOD and GSH levels were decreased in the liver tissues of HFHF rats overexpressing BMI1 (Figure 6J).

## 4 Discussion

E3 ubiquitin ligases and deubiquitinases represent pivotal regulatory components of the ubiquitin‐proteasome system, which plays a critical role in maintaining intracellular protein homeostasis [15]. In the present study, we found that E3 ubiquitin ligase BMI1 is poorly expressed in the liver tissues of rats fed with HFHF. Mechanistically, reduced BMI1 expression failed to promote METTL3 degradation, thereby allowing METTL3 to maintain AHNAK mRNA stability thereby maintaining the stability of this mRNA. AHNAK enhances the liver injury induced by MetS through the TGF‐β/SMAD signaling (Figure 7).

**FIGURE 7:**
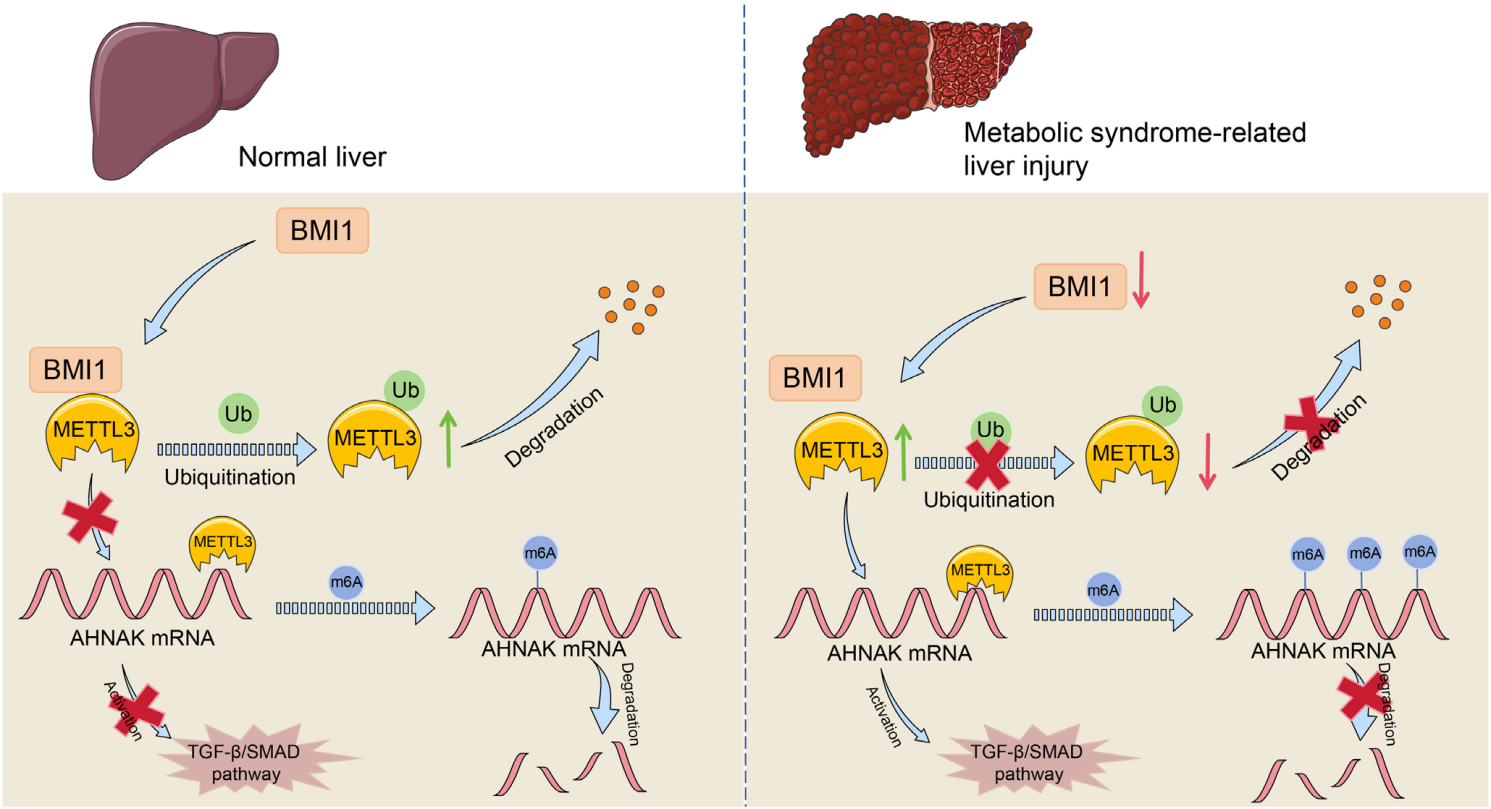
Schematic model. Dysregulated BMI1 expression reduces the ubiquitination of METTL3, leading to increased METTL3 expression. This subsequently enhances m6A methylation of AHNAK mRNA and activates the TGF‐β/SMAD signaling pathway, thereby promoting MetS‐associated liver injury.

Here, we predicted five RNF E3 ubiquitin ligases that might regulate ubiquitination modification of METTL3. Among them, BMI1 showed the highest confidence. Likewise, Okuyama et al. found that BMI1 can modulate IkBa ubiquitination in the cytoplasm [16]. As for its functional role, Herrero et al. found that high BMI1 expression was associated with an adult Sca1^+^ cardiac progenitor subpopulation with low reactive oxygen species levels [17], indicating its association with oxidative stress. The presence of persistent systemic inflammation and oxidative stress has been observed to be associated with MetS, which are hypothesized to play a pivotal role in the mediation of insulin resistance [18, 19]. Oxidative stress is defined as a state in which the antioxidant defense system of the body is unable to properly eliminate reactive oxygen species, and accumulated peroxides further break down into smaller oxidants, such as MDA, which cause irreversible damage to cells and tissues [20]. Indeed, rats exposed to HFHF exhibited signs of insulin resistance, dyslipidemia, and liver injury accompanied by lipid accumulation [21]. Our gain‐of‐function study showed that conditional overexpression of BMI1 alleviated insulin resistance and oxidative stress in the liver of rats fed with HFHF through the degradation of METTL3, since additional overexpression of METTL3 overturned its function. The mRNA and protein expression of METTL3 has been identified to be increased in the liver of piglets after lipopolysaccharide administration [22]. More relevantly, adipose METTL3, influenced by fasting, refeeding, and insulin, was upregulated in HFD‐induced obesity [23]. These findings suggest the broad involvement of METTL3 in liver diseases, either chronic or acute. Overexpression of METTL3 exacerbated HFD‐induced liver metabolic disorders and insulin resistance, while hepatocyte‐specific knockout of METTL3 alleviated HFD‐induced metabolic disorders by slowing weight gain, reducing lipid accumulation, and improving insulin sensitivity [24]. Application of the METTL3 methyltransferase inhibitor, STM2457, increased the level of insulin sensitivity in hepatocytes induced by free fatty acids [25].

Subsequently, we revealed AHNAK as a target of METTL3‐mediated m6A modification. Indeed, knockout of METTL3 in limbal stem cells induced in vivo cell proliferation and migration, thereby expediting the repair of corneal injury, and AHNAK was identified as the m6A target in this process [26]. AHNAK is a ~5600 amino acid tripartite protein composed of three distinct domains: amino‐terminal, carboxyl‐terminal, and central [27]. Interestingly, Kim et al. showed that AHNAK knockout mice exhibited reduced fat accumulation in the liver and decreased serum TG levels when provided with either a normal chow diet or an HFD [28]. In addition, AHNAK depletion accelerated liver regeneration after 70% partial hepatectomy by blocking the TGF‐β/SMAD signaling [29]. Moreover, both BMI1 and METTL3 have been reported to regulate the TGF‐β/SMAD signaling [30, 31]. TGF‐β signaling plays a significant yet complex role in regulating energy homeostasis and chronic liver conditions, ranging from simple steatosis to severe metabolic dysfunction‐associated steatohepatitis, and TGF‐β also drives fibrosis and cancer through aberrant SMAD2/3 activation [32]. The expression of TGF‐β1 and p‐Smad3 was found after 2 years of feeding rhesus monkeys with HFD [33]. These findings raise an interesting question: whether BMI1 blocks AHNAK‐mediated TGF‐β/SMAD signaling through the ubiquitination modification of METTL3 in hepatocytes. After verifying the indirect modulation of AHNAK by BMI1, we observed that the deficit of AHNAK‐mediated TGF‐β/SMAD signaling was rescued by overexpression of METTL3, which substantiated our hypothesis.

In the present study, the serum levels of ALT, TG, and glucose exhibited statistically significant disparities between the groups, although the absolute values remained within the normal physiological range. These findings suggest the presence of early metabolic and hepatic changes that may precede overt pathological elevations. Such subtle shifts are of particular significance in preclinical models, as they reflect the underlying metabolic stress and align with previous reports of early alterations in HFHF‐induced MetS [34, 35, 36].

## 5 Conclusion

In summary, we demonstrated that BMI1 overexpression effectively alleviates MetS in HFHF‐fed rats, and the anti‐MetS effect of BMI1 overexpression is related to the modulation of AHNAK‐mediated TGF‐β/SMAD signaling through the ubiquitination modification of METTL3.

## Funding

This work was supported by Shanghai “Rising Stars of Medical Talents” Youth Development Program (SHWSRS(2024)_70).

## Conflicts of Interest

The authors declare no conflicts of interest.

## Supporting information


**Figure S1:** Analysis of the subcellular localization of AHNAK. (A) The expression of AHNAK mRNA in the cytoplasm and nucleus following METTL3 overexpression was examined using FISH. (B) AHNAK protein expression in the nucleus and cytoplasm following METTL3 overexpression was examined using Western blot analysis. (C) Expression of AHNAK in liver samples from the Human Protein Atlas project. Data are presented as means ± SEM (*n* = 5), and statistical significance was assessed using an unpaired *t*‐test.

**Data S1:** Supporting Information.

## Data Availability

The datasets used and/or analyzed in the current study are available from the corresponding author upon reasonable request.
