# Supplementary material for: BMI1 Overexpression Alleviates Metabolic Syndrome‐Related Liver Injury by Inhibiting the Activation of the METTL3/AHNAK Axis
Source: Kaohsiung J Med Sci. 2026 Oct 26:e70308. Online ahead of print. doi: 10.1002/kjm2.70308 (PMC13697295; doi:10.1002/kjm2.70308)
Supplement: Supplementary file 1 [file KJM2-9999-e70308-s002.pdf]

Original western blots (Fig 2A,2B,3A,6A)

Fig 2A

RAT #1

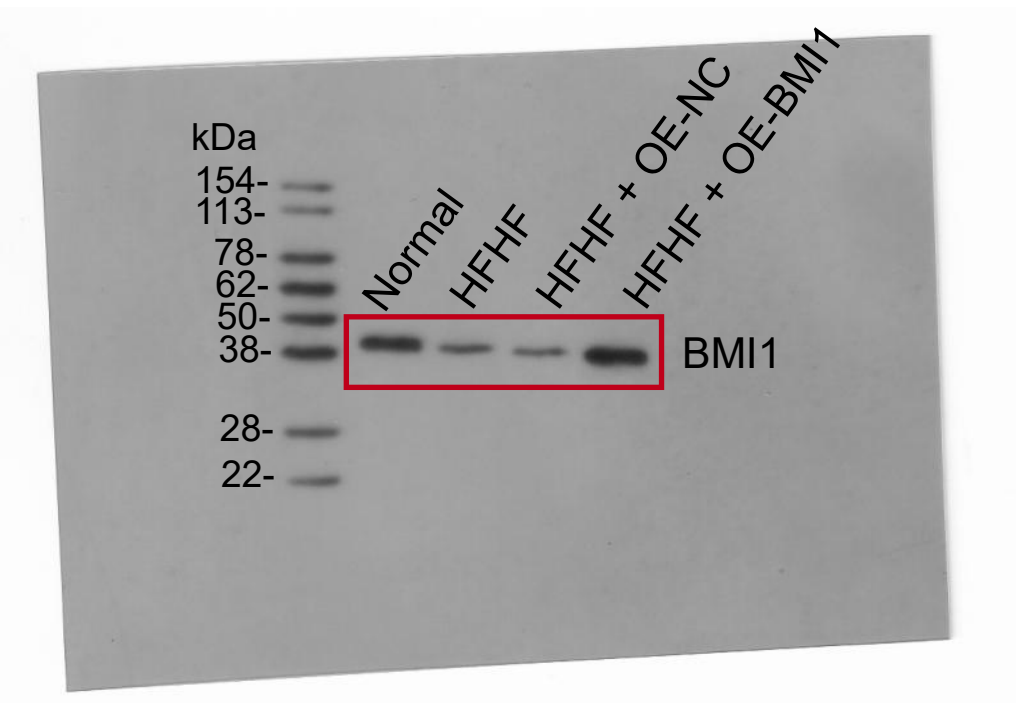

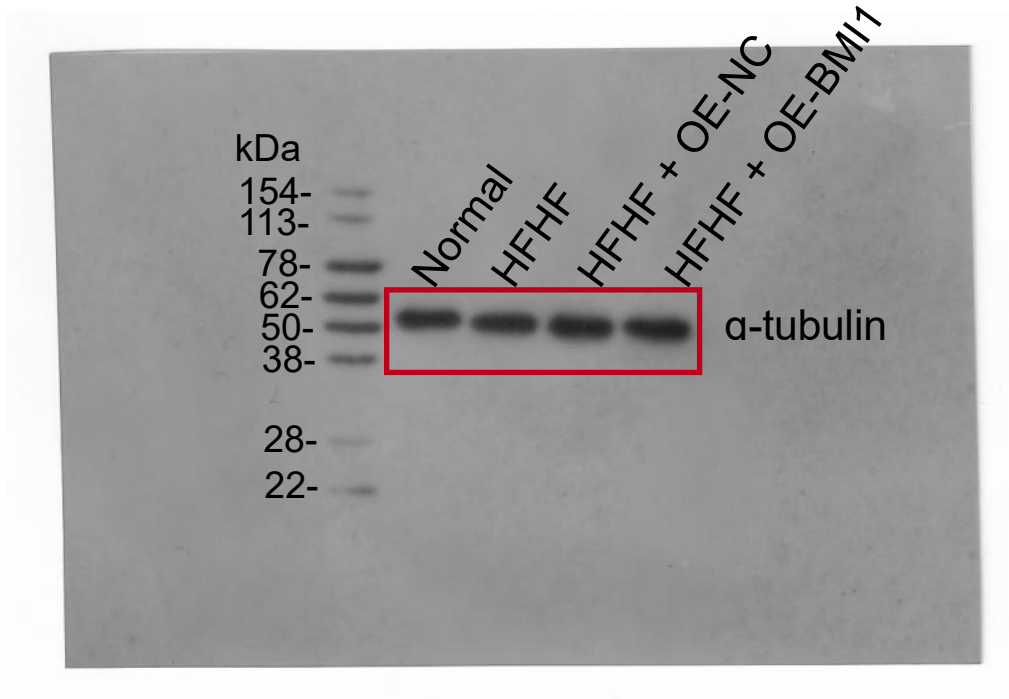

RAT #2

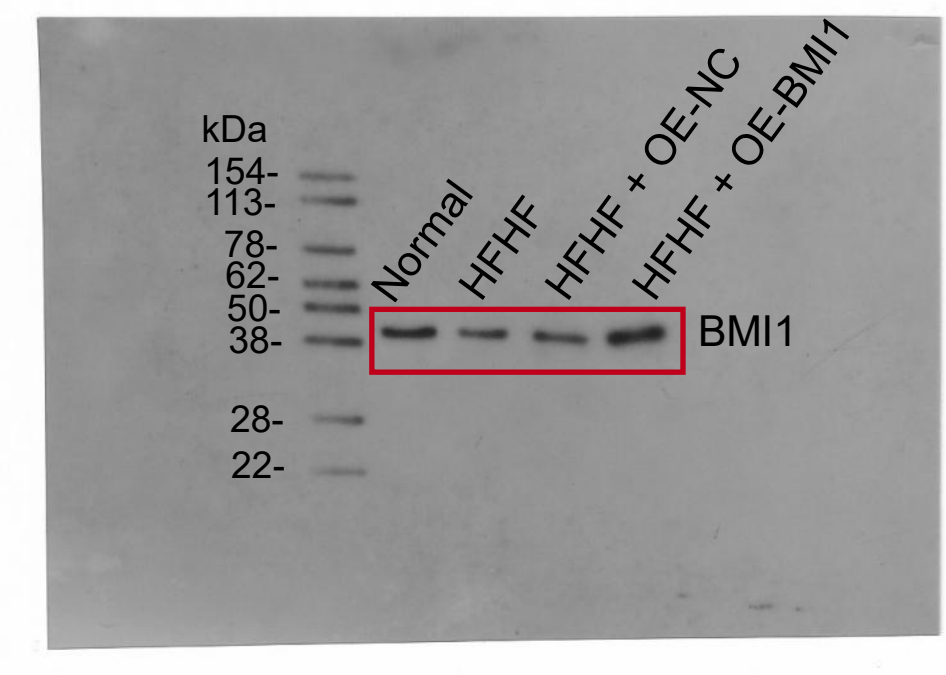

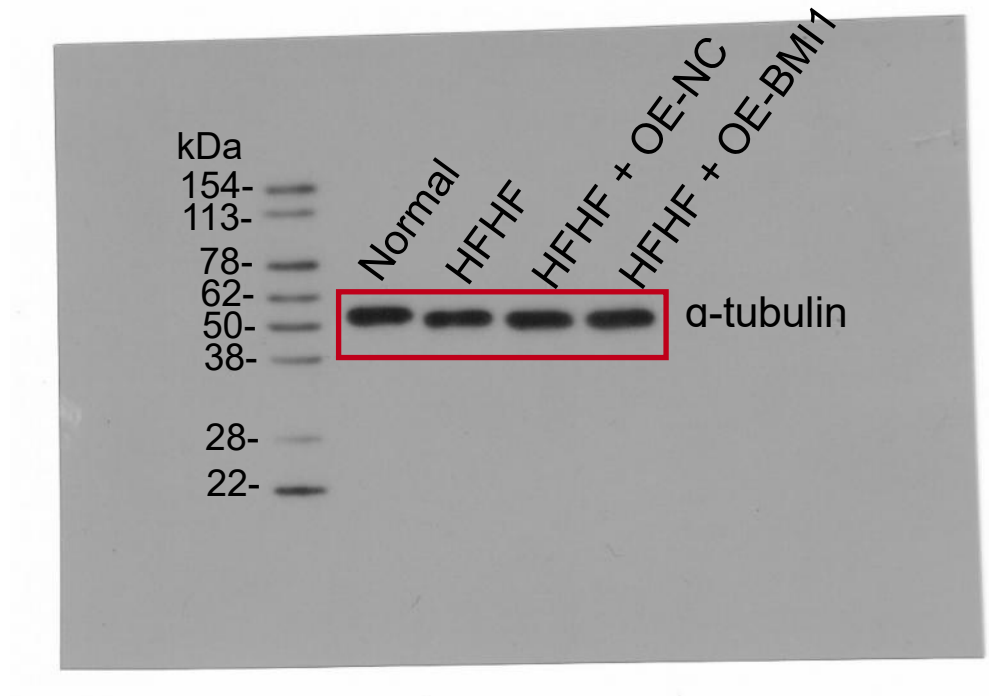

RAT #3

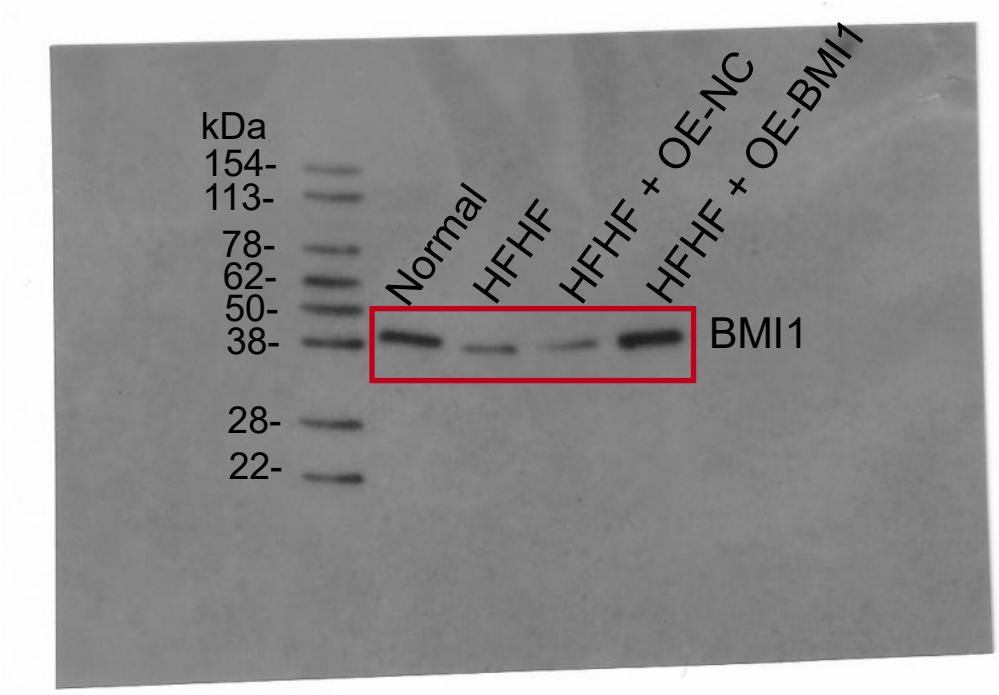

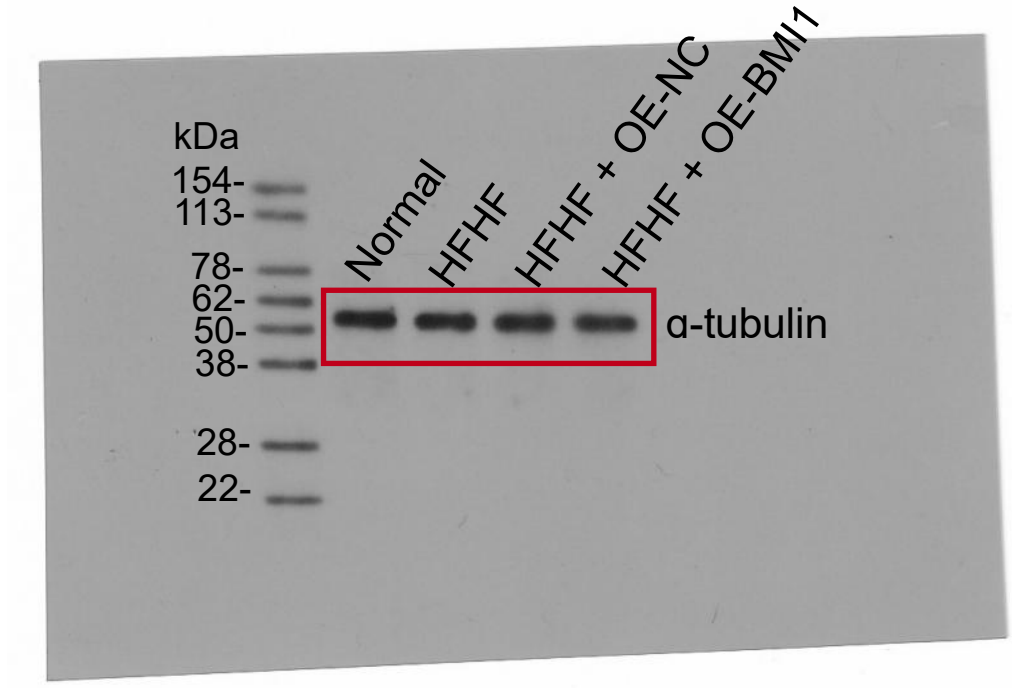

RAT #4

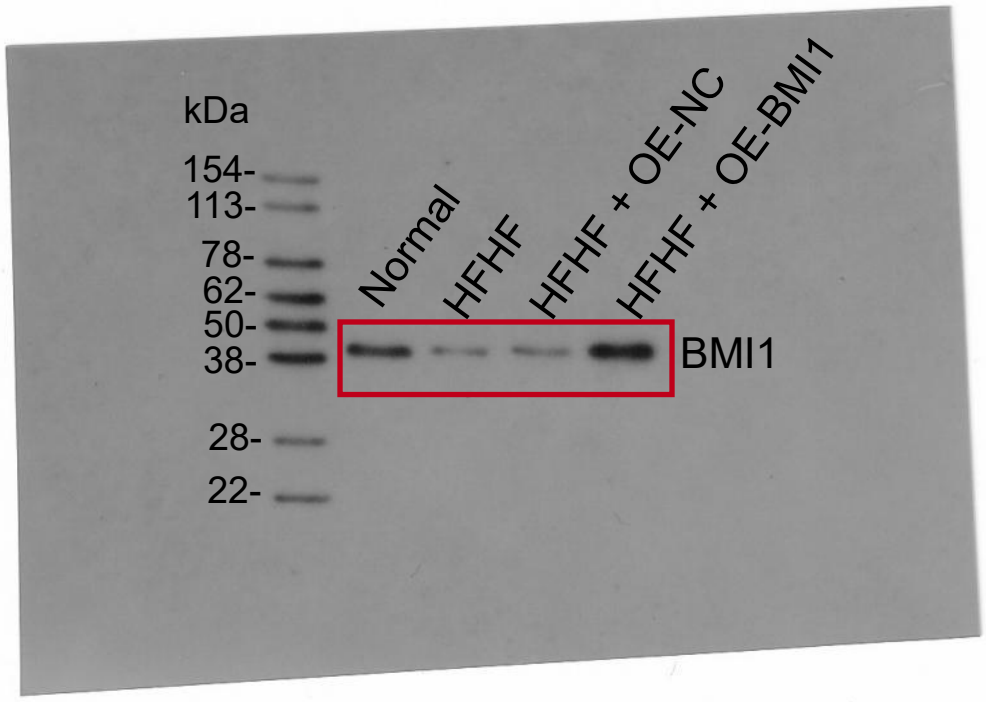

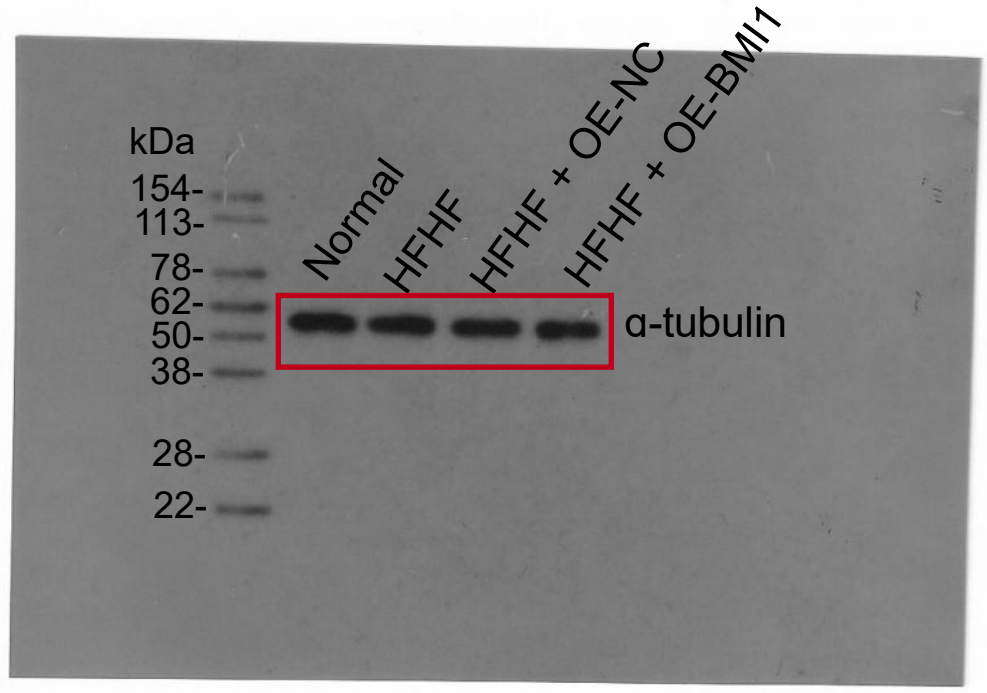

RAT #5

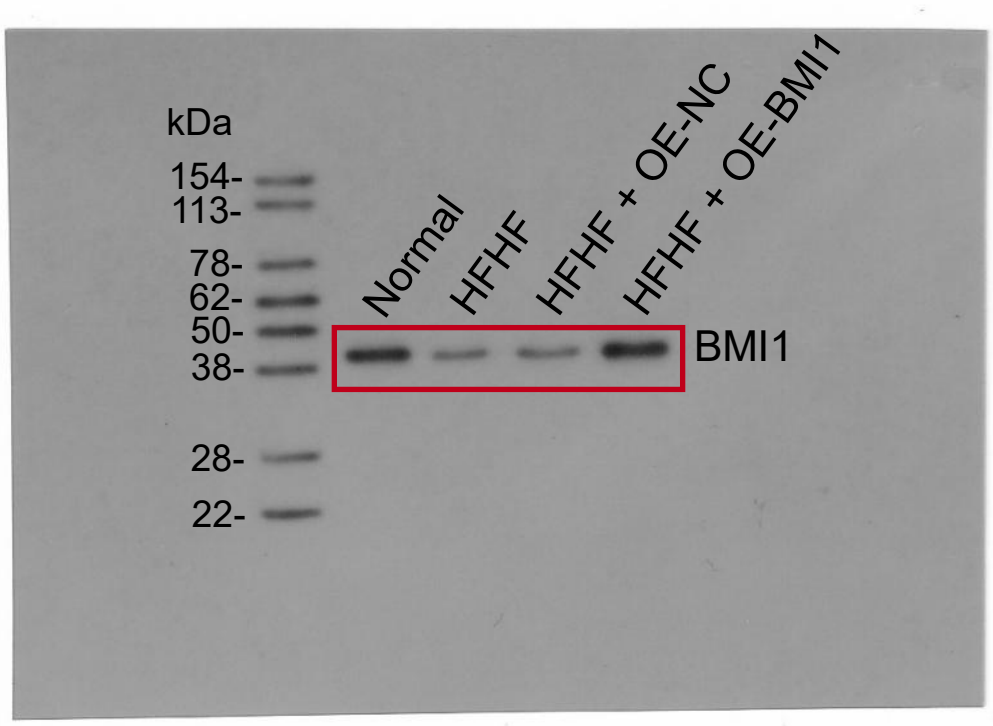

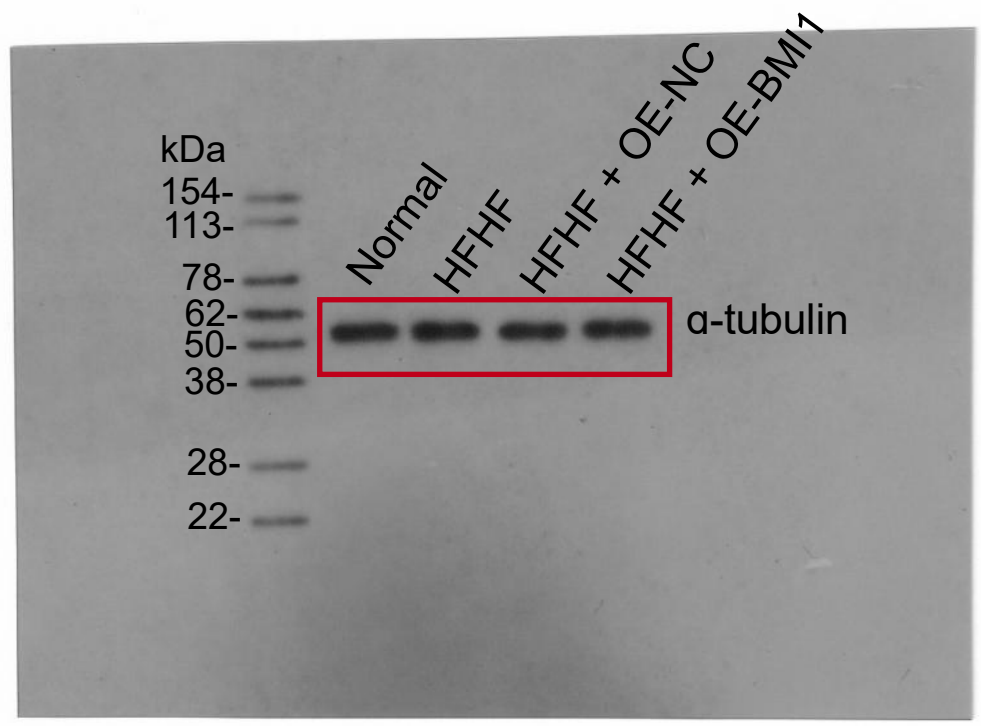

RAT #6

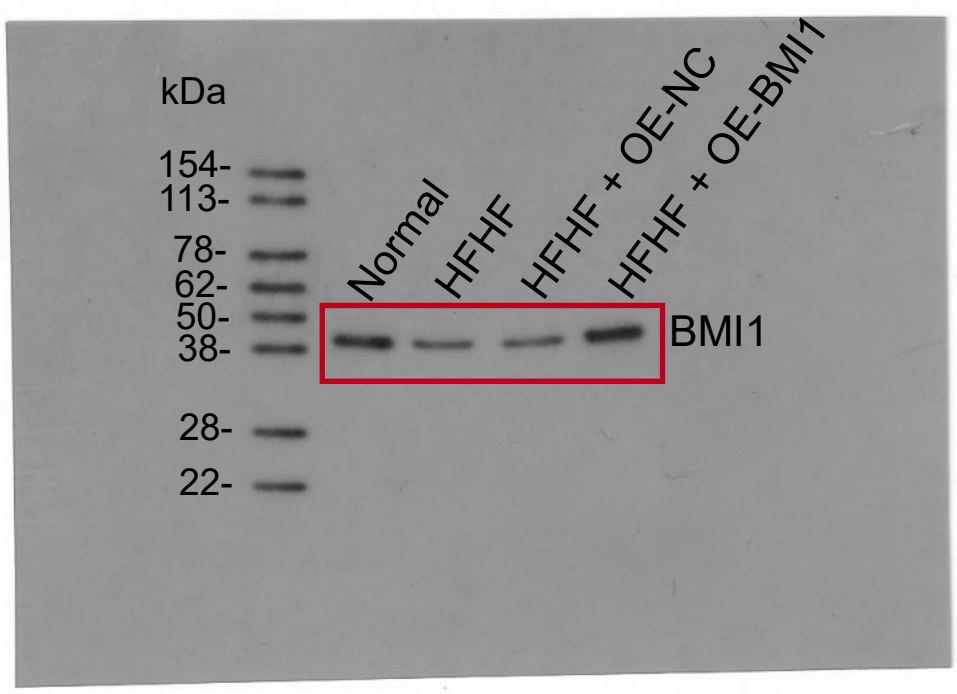

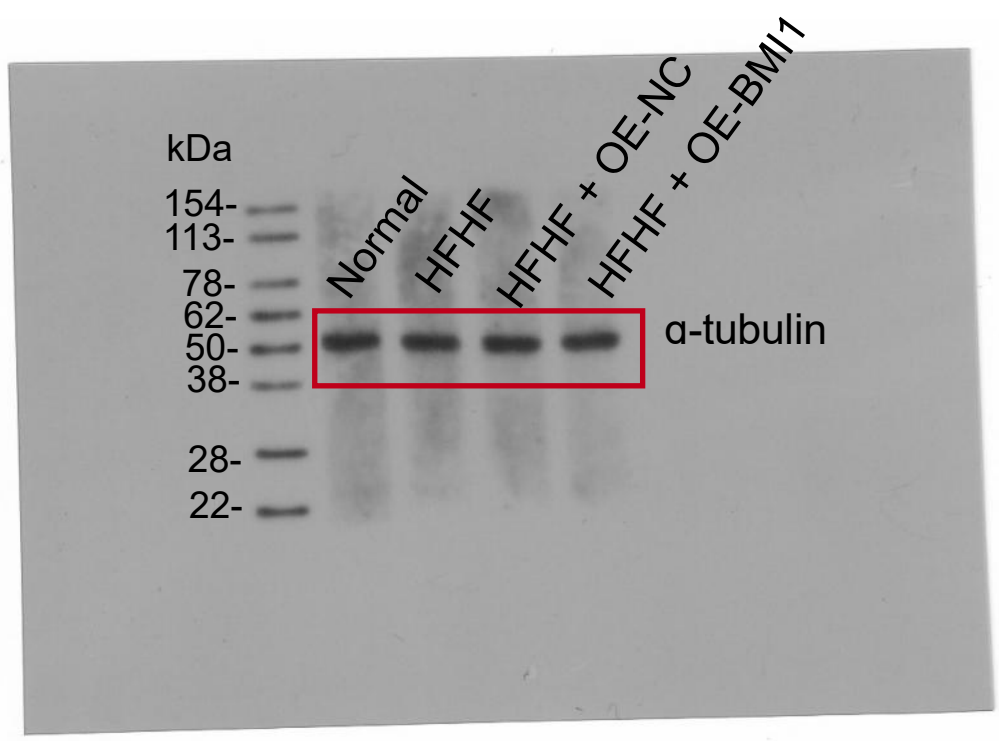

Fig 2B

RAT #1

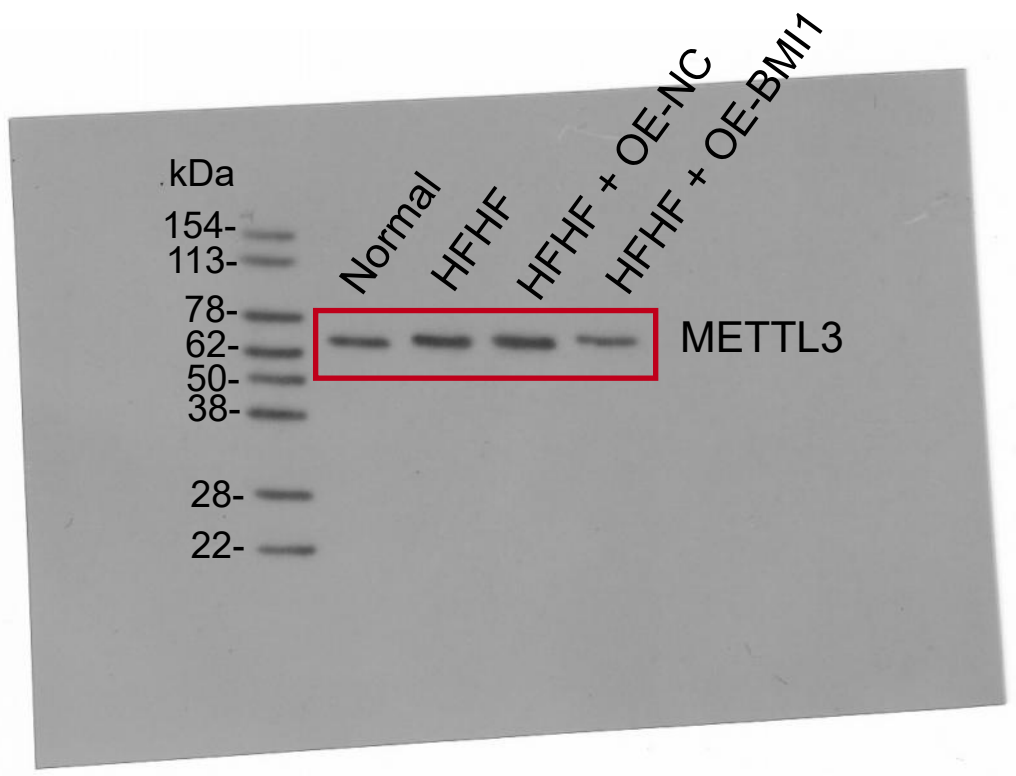
kDa
154-
113-
78-
62-
50-
38-
28-
22-
Normal
HFHF
HFHF + OE-NC
HFHF + OE-BMI1
METTL3

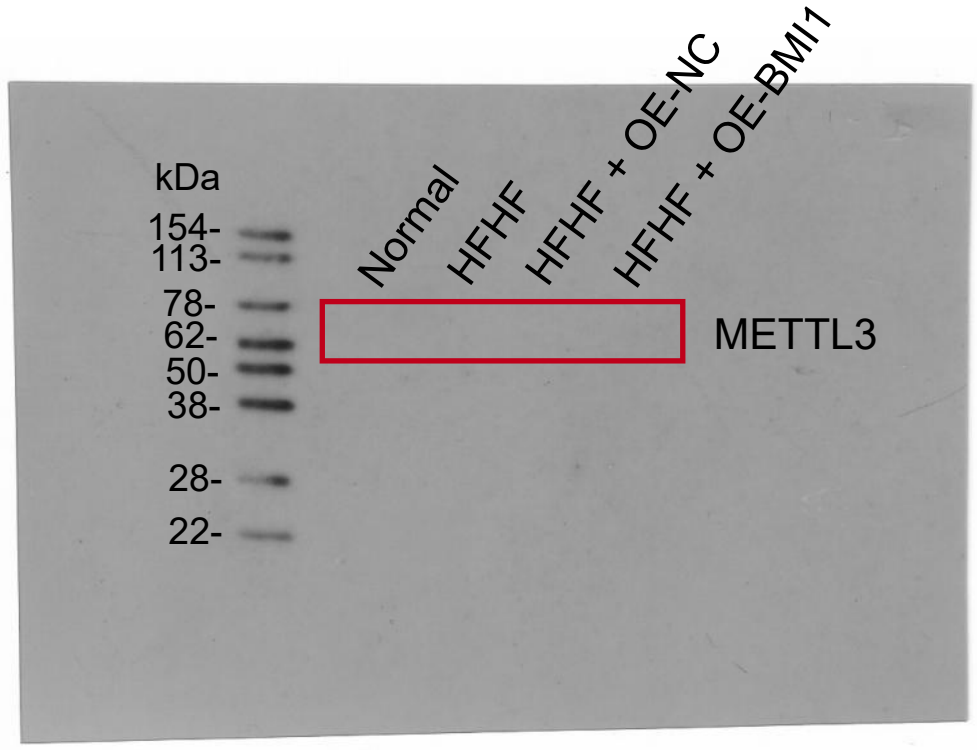
kDa
154-
113-
78-
62-
50-
38-
28-
22-
Normal
HFHF
HFHF + OE-NC
HFHF + OE-BMI1
METTL3

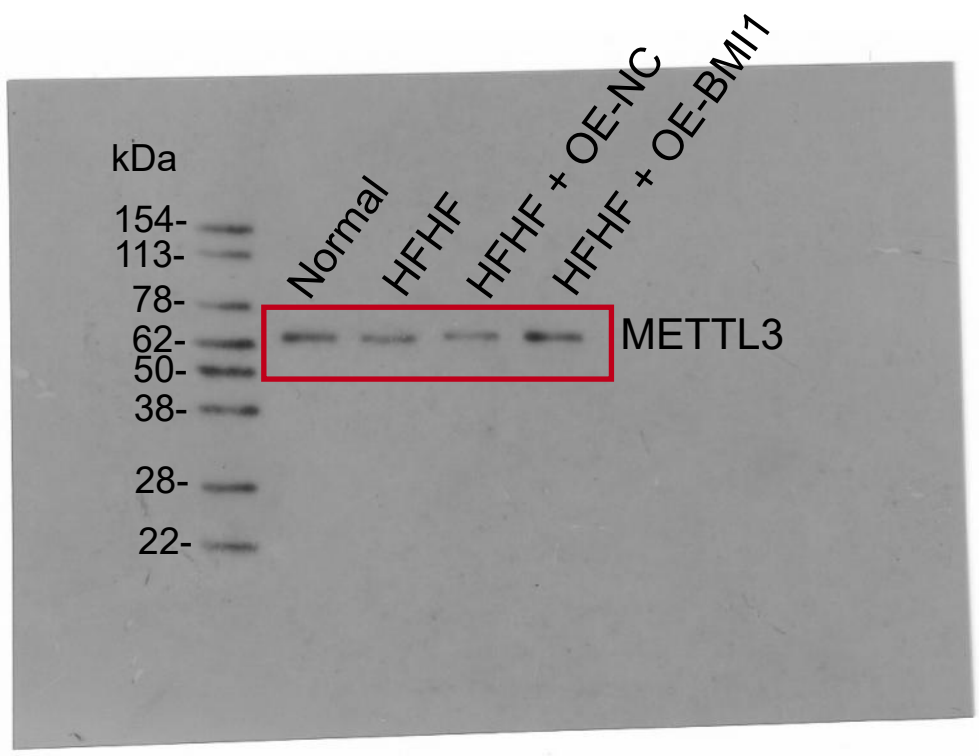
kDa
154-
113-
78-
62-
50-
38-
28-
22-
Normal
HFHF
HFHF + OE-NC
HFHF + OE-BMI1
METTL3

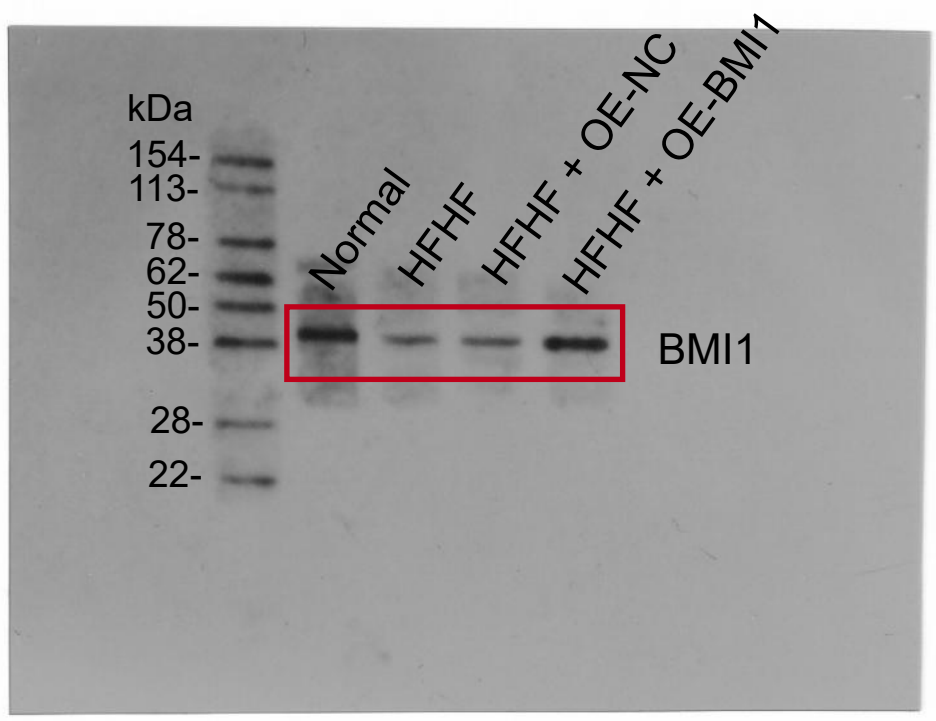
kDa
154-
113-
78-
62-
50-
38-
28-
22-
Normal
HFHF
HFHF + OE-NC
HFHF + OE-BMI1
BMI1

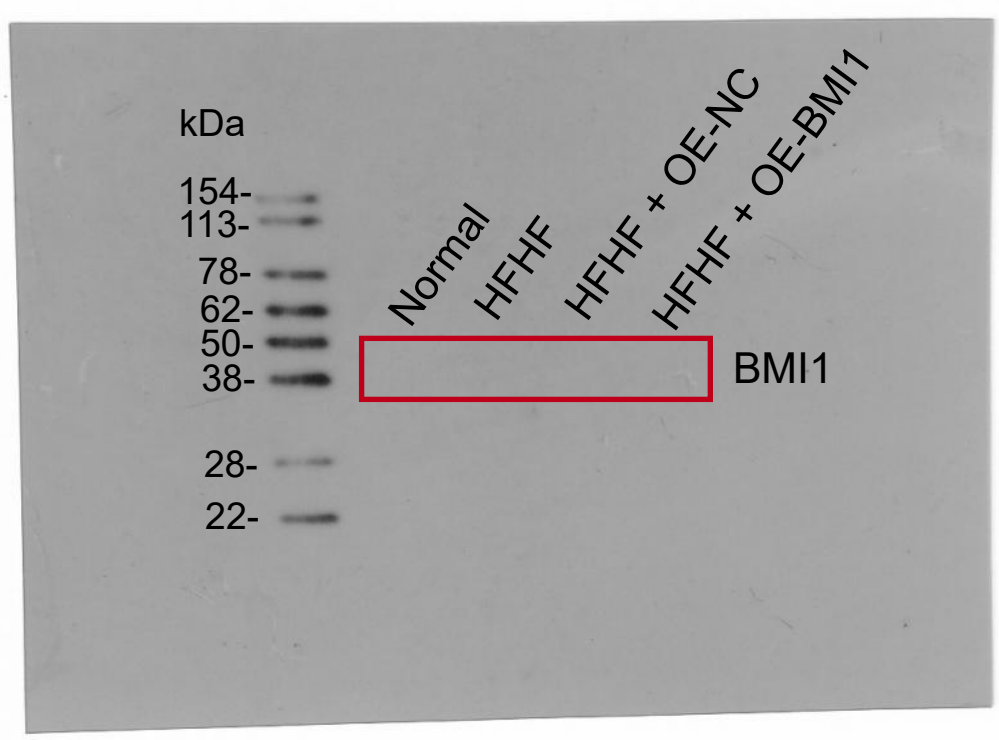
kDa
154-
113-
78-
62-
50-
38-
28-
22-
Normal
HFHF
HFHF + OE-NC
HFHF + OE-BMI1
BMI1

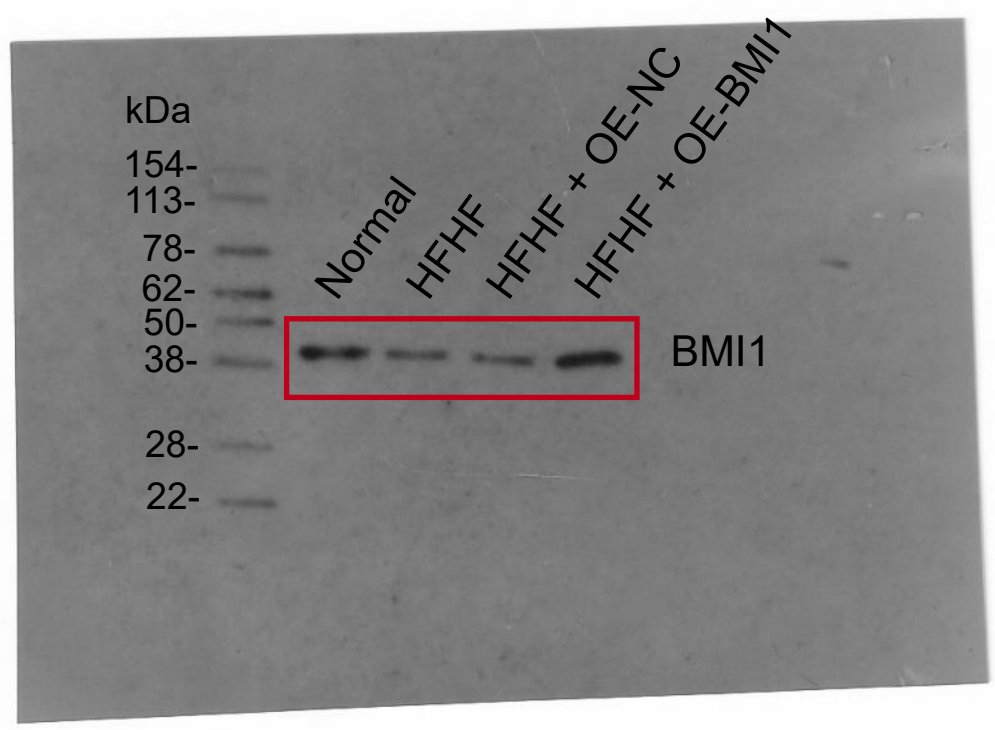
kDa
154-
113-
78-
62-
50-
38-
28-
22-
Normal
HFHF
HFHF + OE-NC
HFHF + OE-BMI1
BMI1

RAT #2

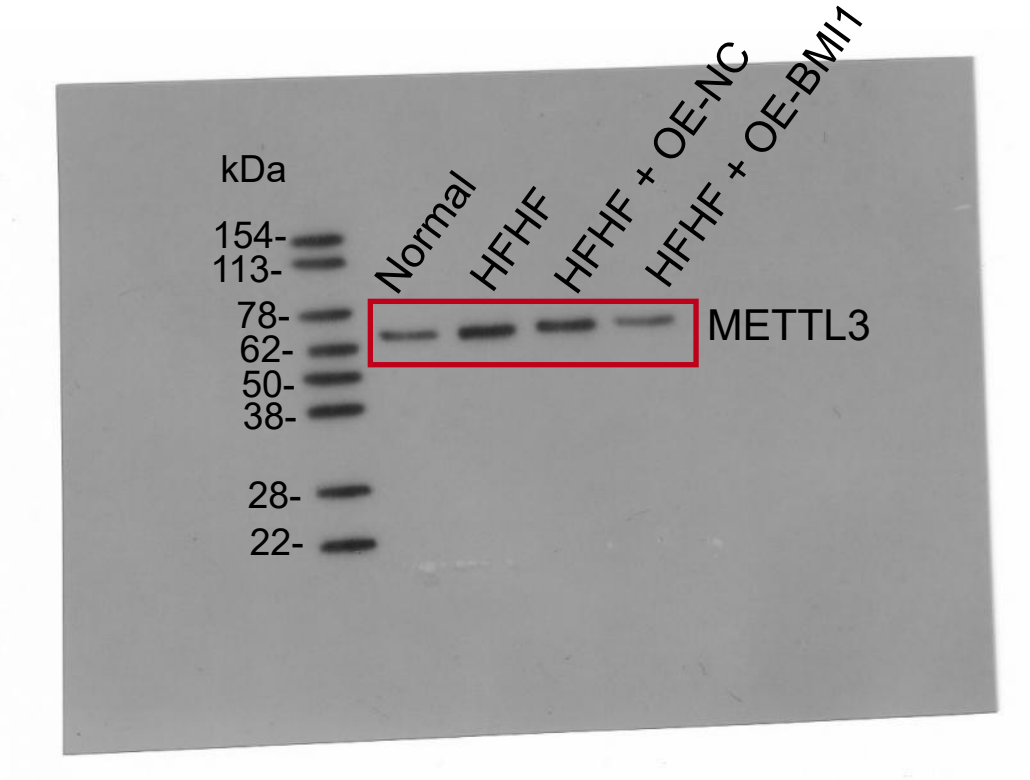

kDa
154-
113-
78-
62-
50-
38-
28-
22-
Normal
HFHF
HFHF + OE-NC
HFHF + OE-BMI1
METTL3

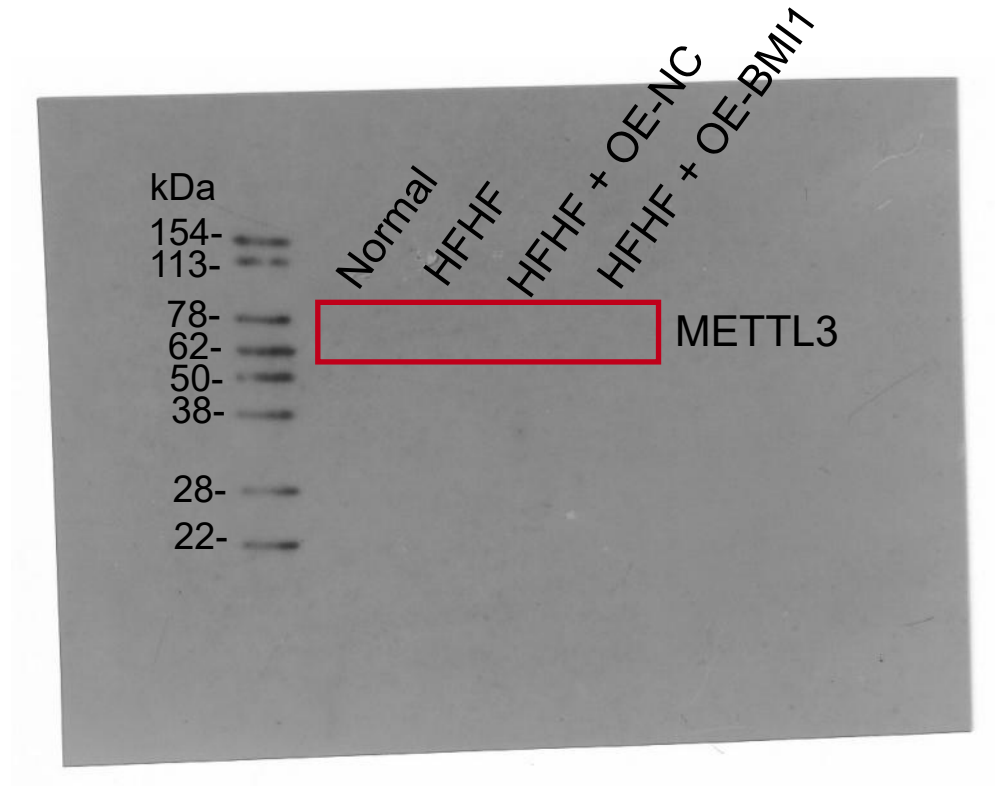

kDa
154-
113-
78-
62-
50-
38-
28-
22-
Normal
HFHF
HFHF + OE-NC
HFHF + OE-BMI1
METTL3

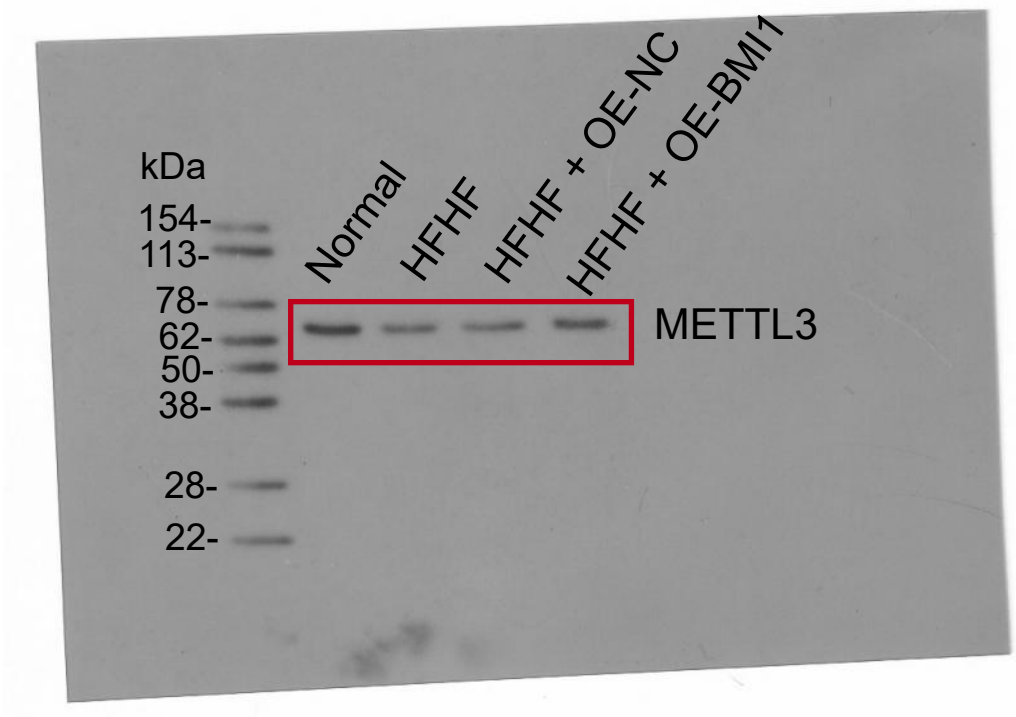

kDa
154-
113-
78-
62-
50-
38-
28-
22-
Normal
HFHF
HFHF + OE-NC
HFHF + OE-BMI1
METTL3

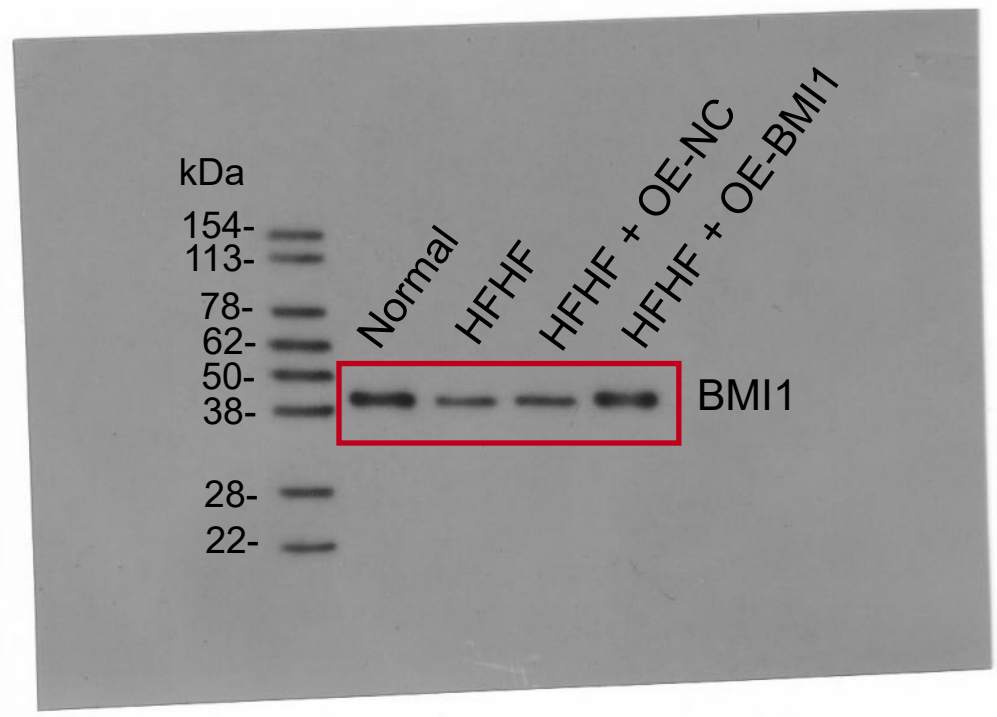

kDa
154-
113-
78-
62-
50-
38-
28-
22-
Normal
HFHF
HFHF + OE-NC
HFHF + OE-BMI1
BMI1

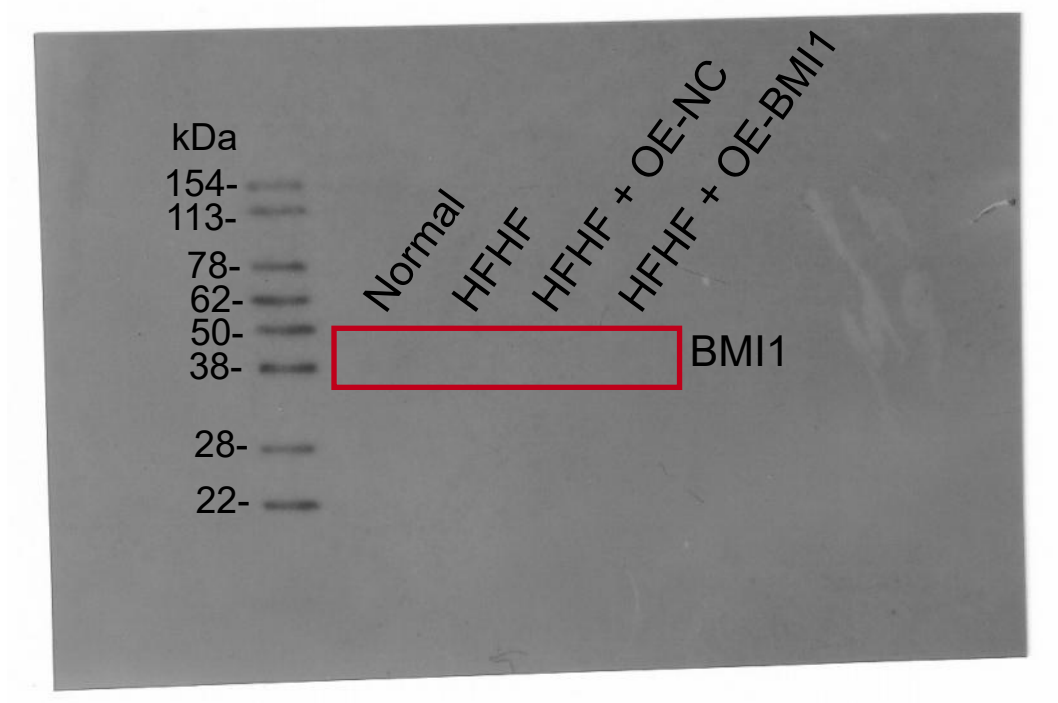

kDa
154-
113-
78-
62-
50-
38-
28-
22-
Normal
HFHF
HFHF + OE-NC
HFHF + OE-BMI1
BMI1

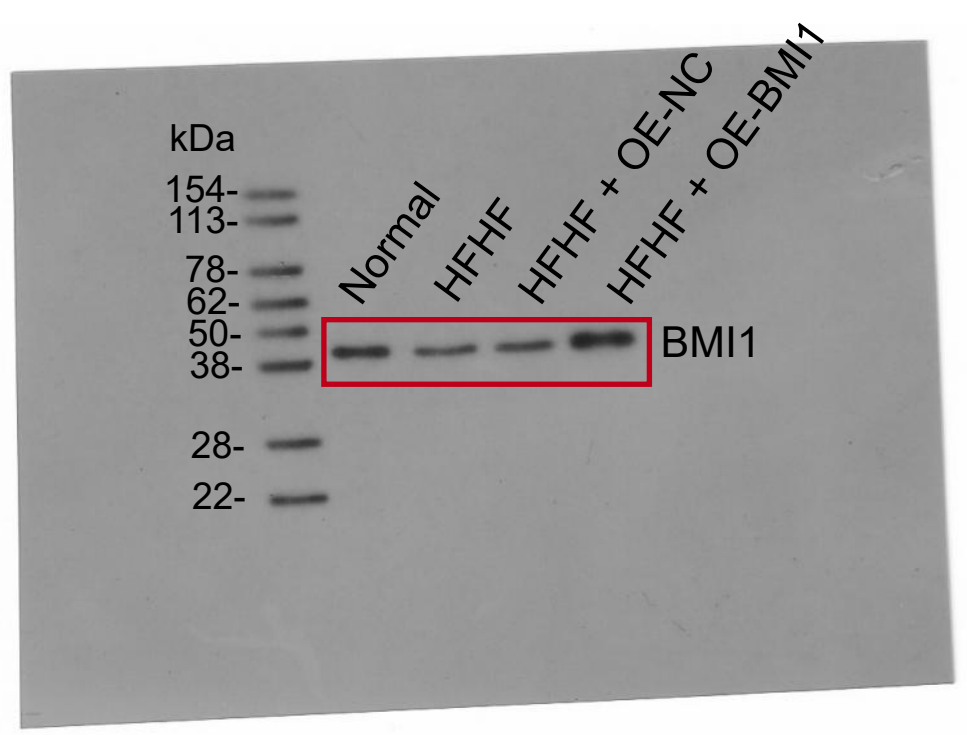

kDa
154-
113-
78-
62-
50-
38-
28-
22-
Normal
HFHF
HFHF + OE-NC
HFHF + OE-BMI1
BMI1

RAT #3

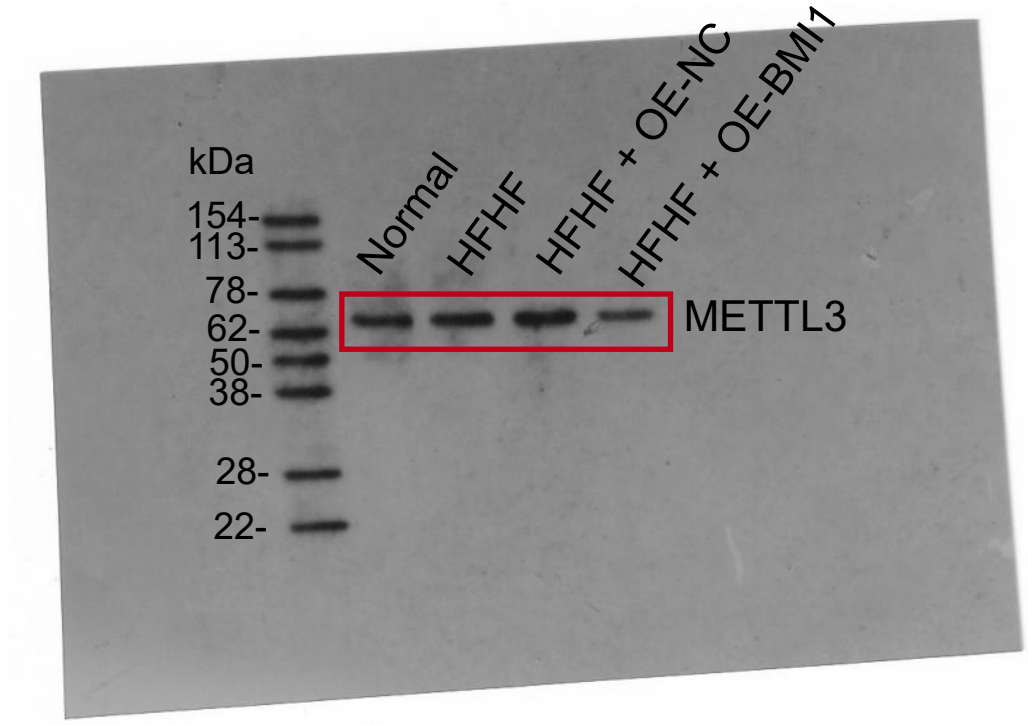
kDa
154-
113-
78-
62-
50-
38-
28-
22-
Normal
HFHF
HFHF + OE-NC
HFHF + OE-BMI1
METTL3

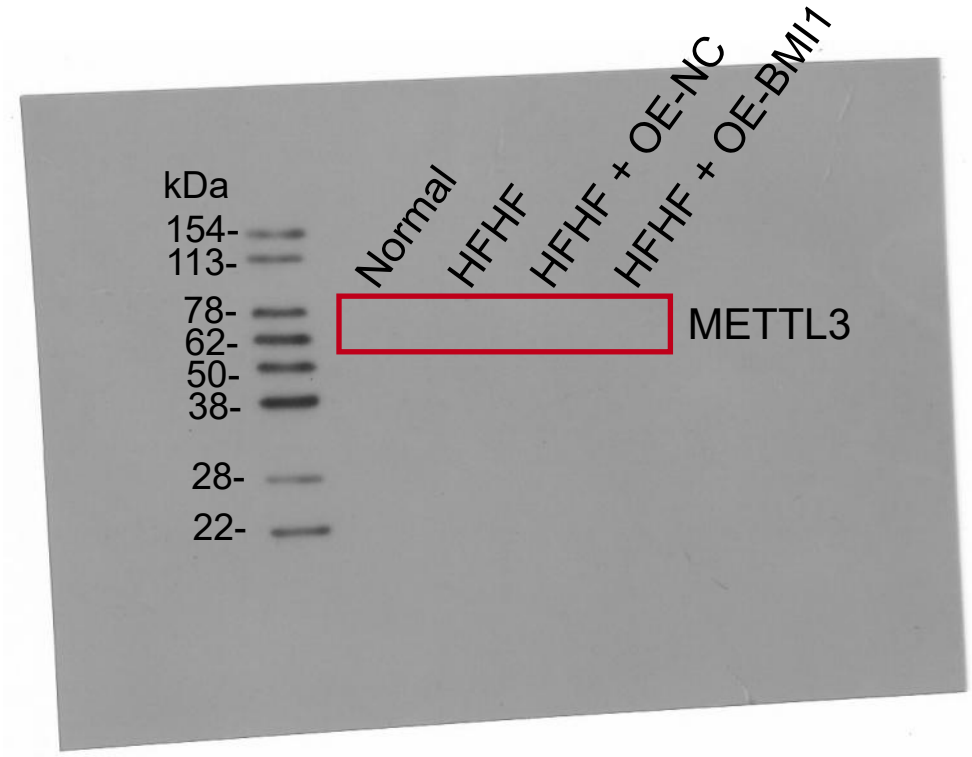
kDa
154-
113-
78-
62-
50-
38-
28-
22-
Normal
HFHF
HFHF + OE-NC
HFHF + OE-BMI1
METTL3

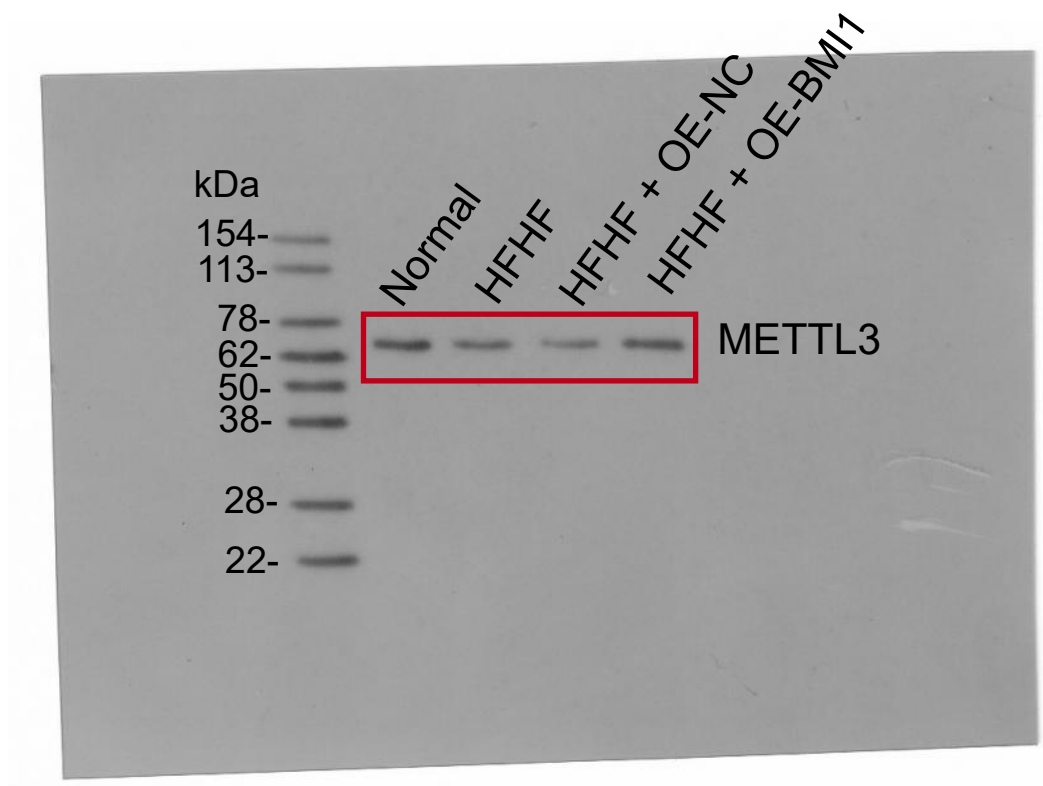
kDa
154-
113-
78-
62-
50-
38-
28-
22-
Normal
HFHF
HFHF + OE-NC
HFHF + OE-BMI1
METTL3

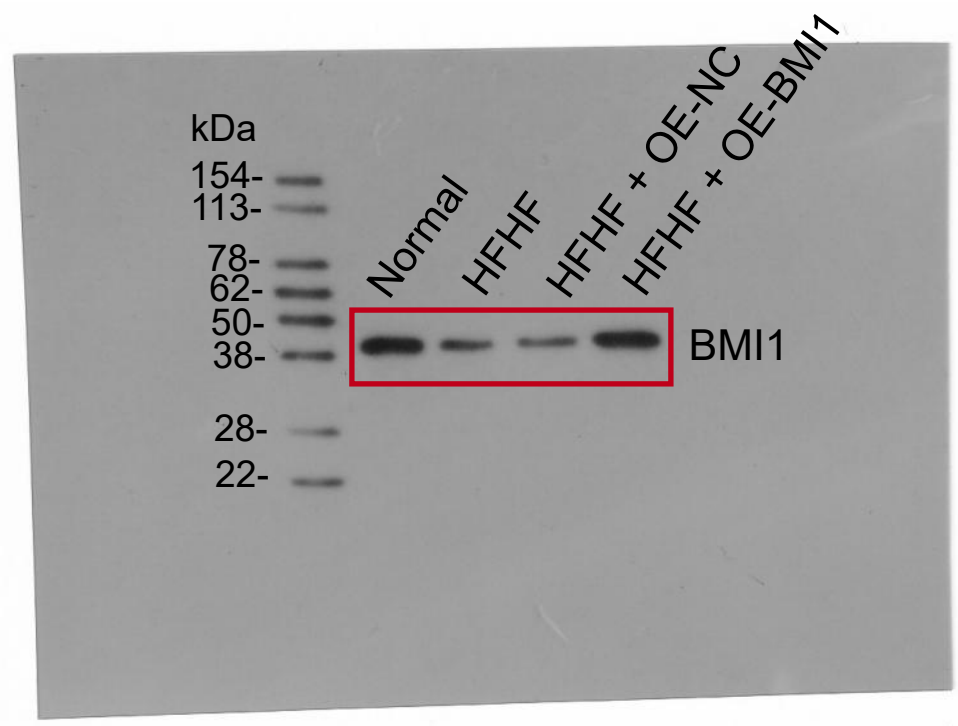
kDa
154-
113-
78-
62-
50-
38-
28-
22-
Normal
HFHF
HFHF + OE-NC
HFHF + OE-BMI1
BMI1

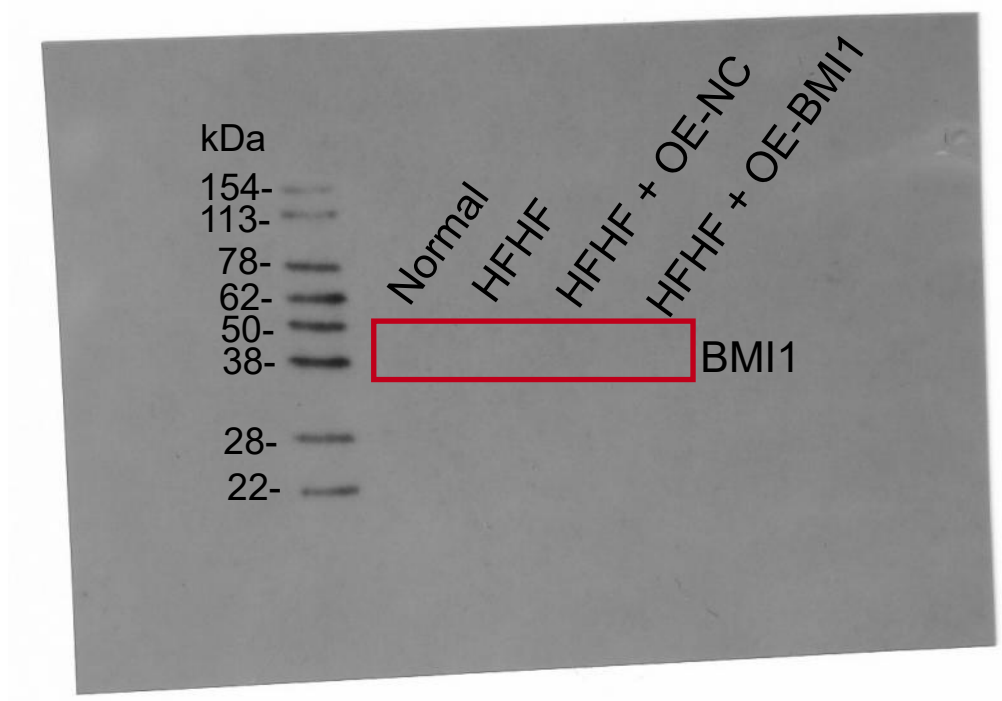
kDa
154-
113-
78-
62-
50-
38-
28-
22-
Normal
HFHF
HFHF + OE-NC
HFHF + OE-BMI1
BMI1

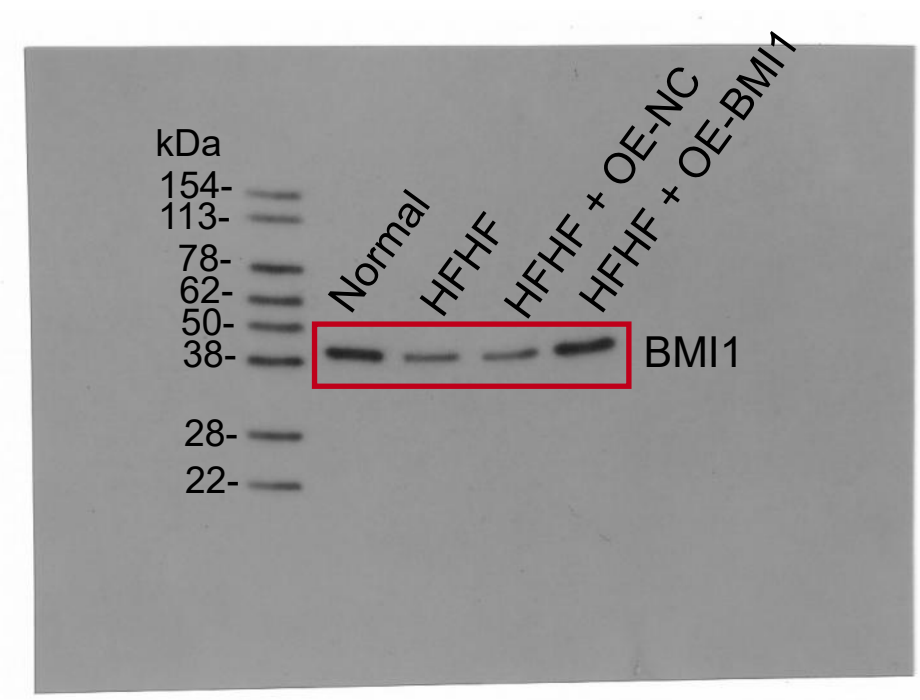
kDa
154-
113-
78-
62-
50-
38-
28-
22-
Normal
HFHF
HFHF + OE-NC
HFHF + OE-BMI1
BMI1

RAT #4

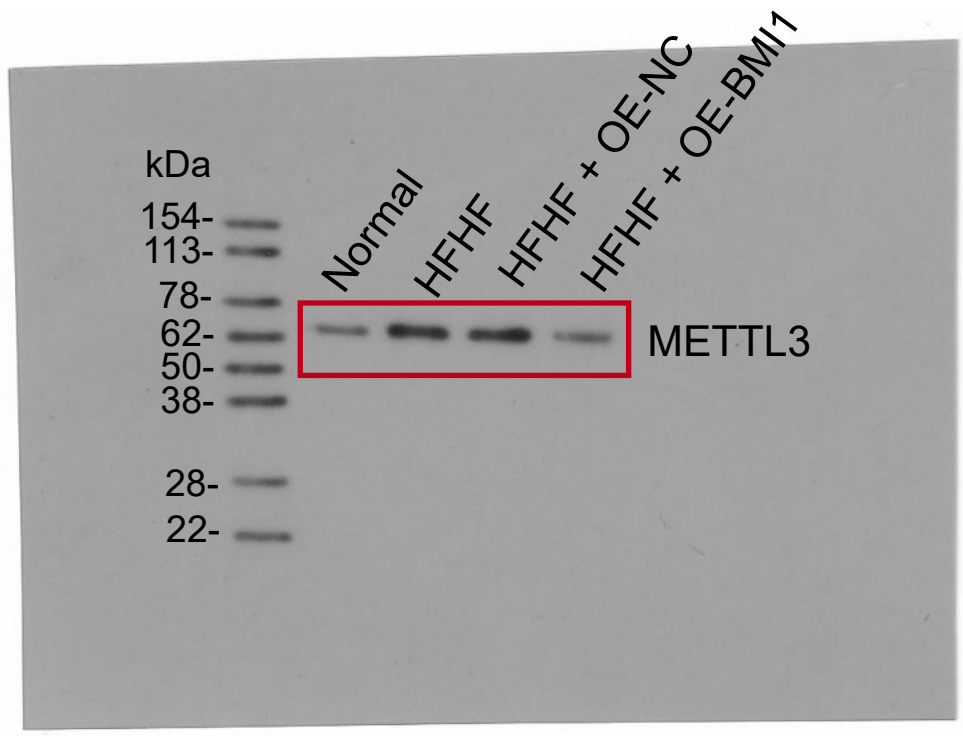
kDa
154-
113-
78-
62-
50-
38-
28-
22-
Normal
HFHF
HFHF + OE-NC
HFHF + OE-BMI1
METTL3

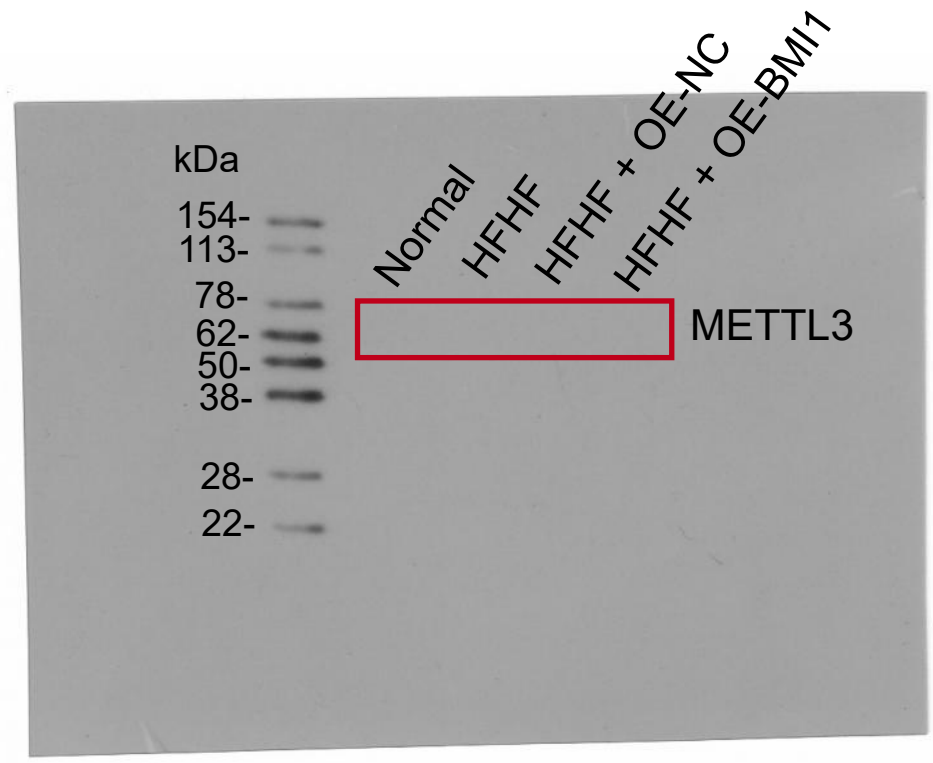
kDa
154-
113-
78-
62-
50-
38-
28-
22-
Normal
HFHF
HFHF + OE-NC
HFHF + OE-BMI1
METTL3

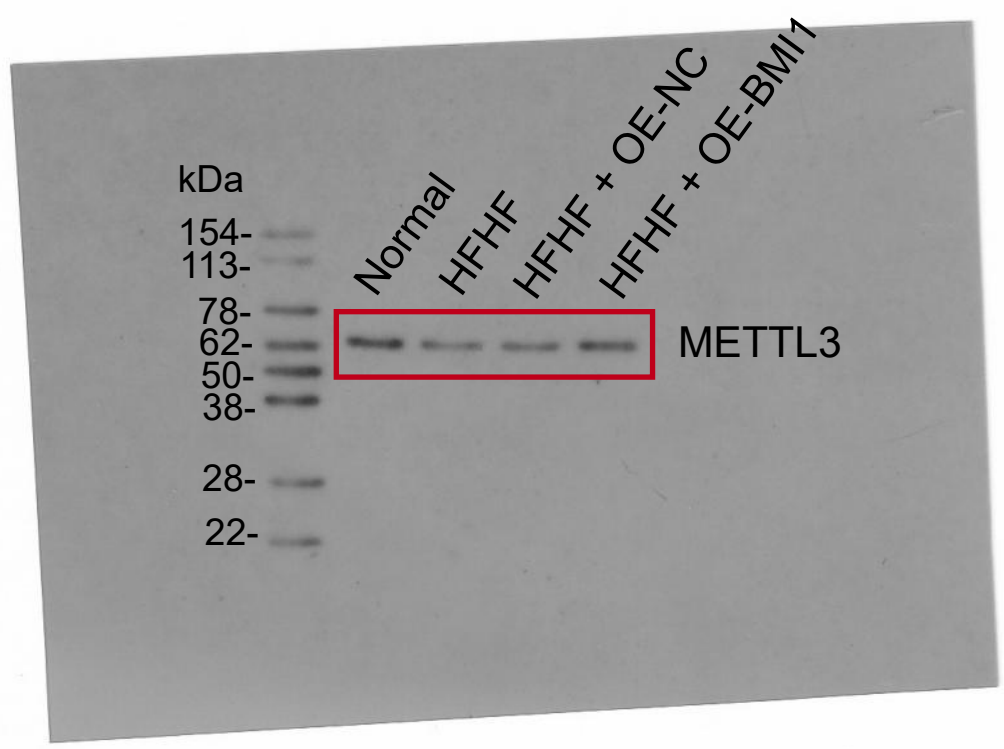
kDa
154-
113-
78-
62-
50-
38-
28-
22-
Normal
HFHF
HFHF + OE-NC
HFHF + OE-BMI1
METTL3

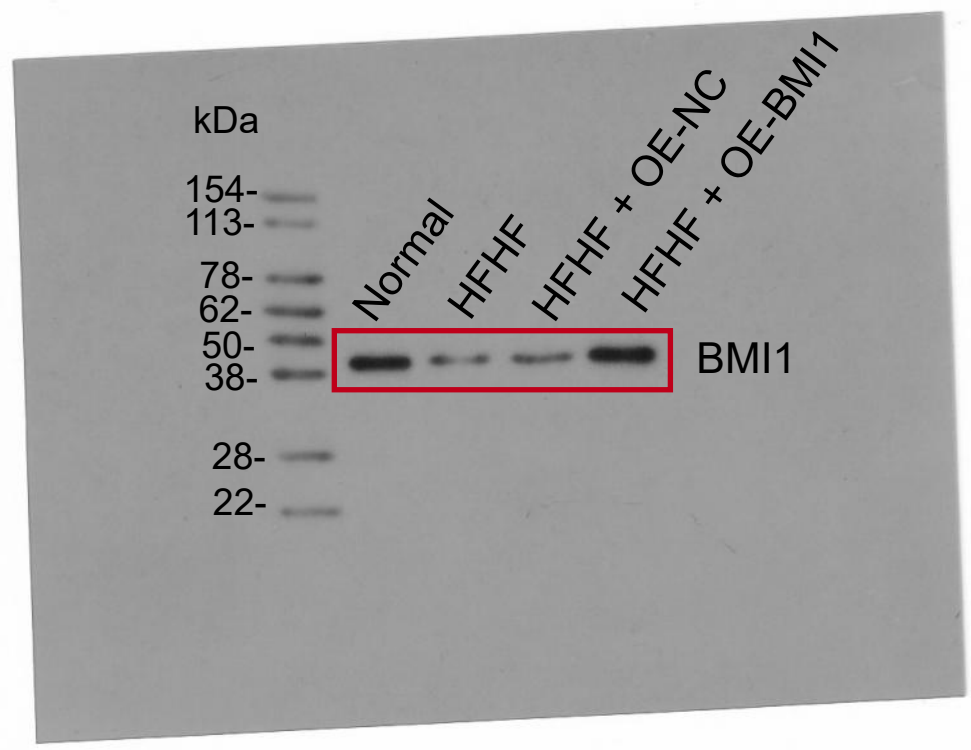
kDa
154-
113-
78-
62-
50-
38-
28-
22-
Normal
HFHF
HFHF + OE-NC
HFHF + OE-BMI1
BMI1

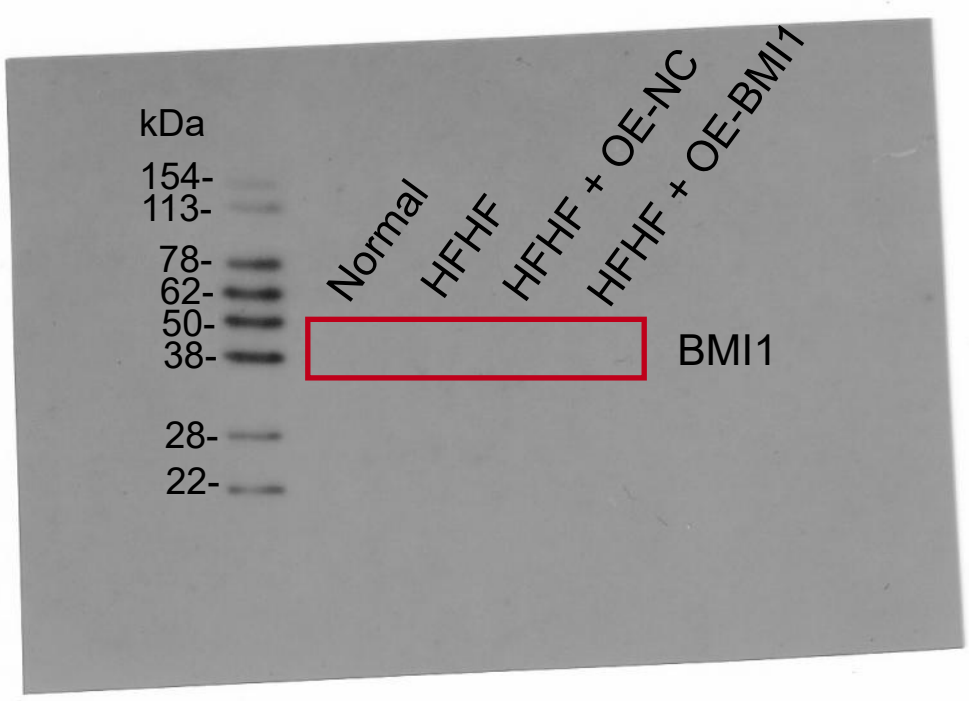
kDa
154-
113-
78-
62-
50-
38-
28-
22-
Normal
HFHF
HFHF + OE-NC
HFHF + OE-BMI1
BMI1

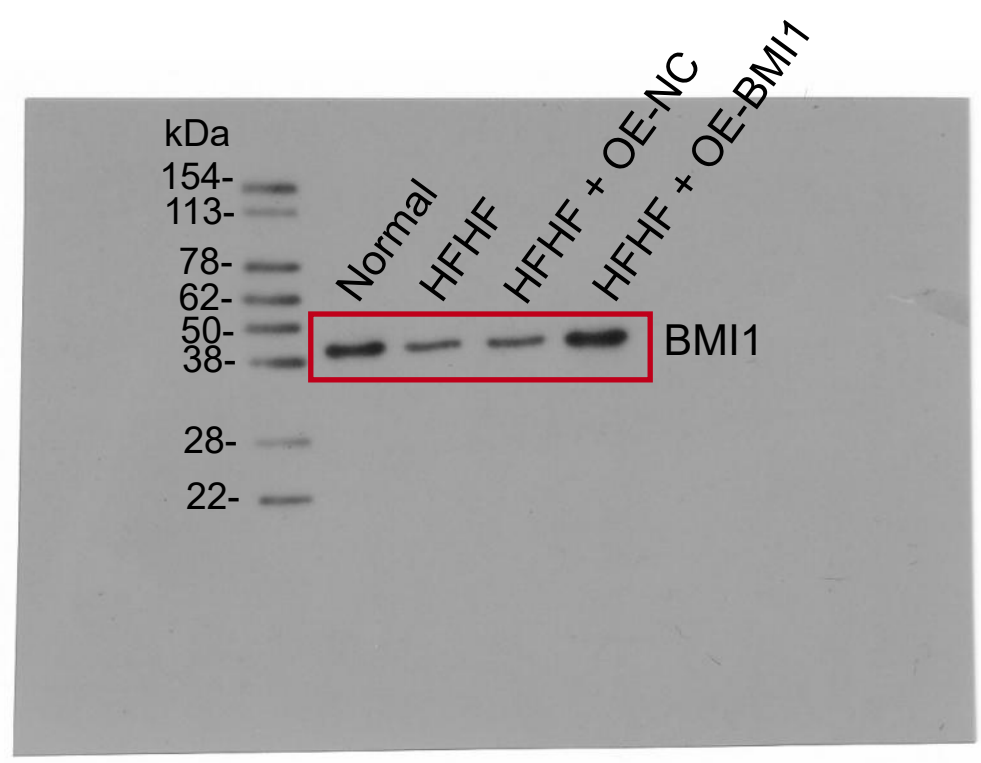
kDa
154-
113-
78-
62-
50-
38-
28-
22-
Normal
HFHF
HFHF + OE-NC
HFHF + OE-BMI1
BMI1

RAT #5

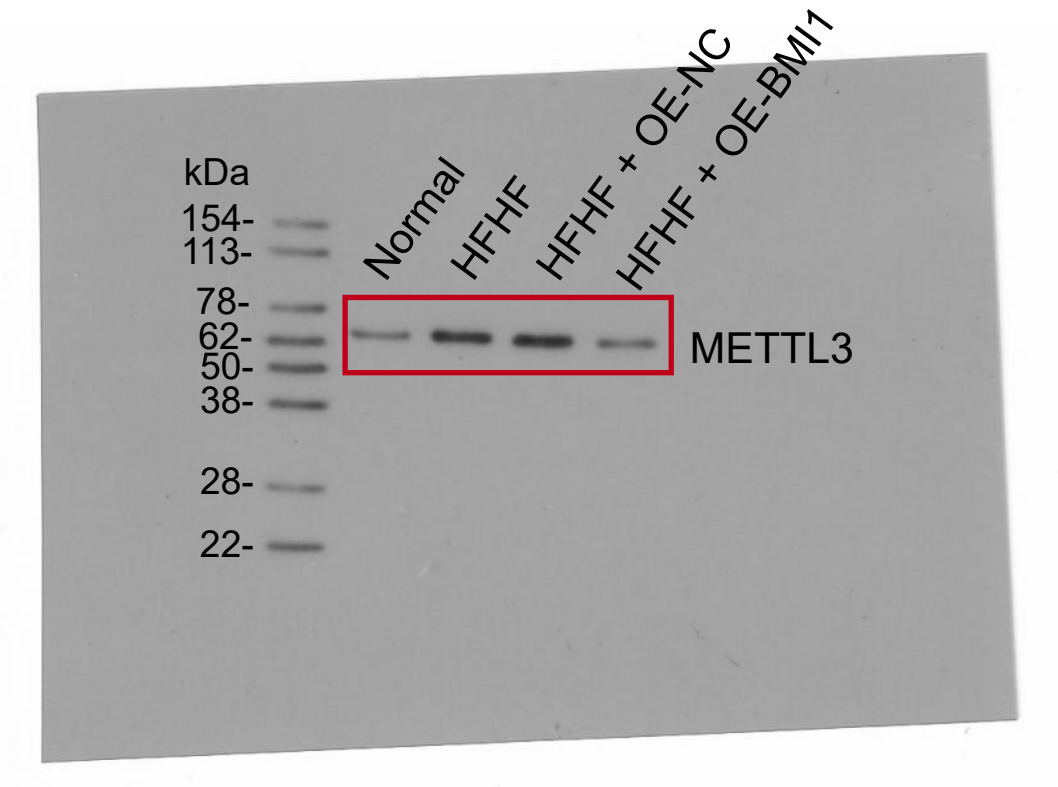
kDa
154-
113-
78-
62-
50-
38-
28-
22-
Normal
HFHF
HFHF + OE-NC
HFHF + OE-BMI1
METTL3

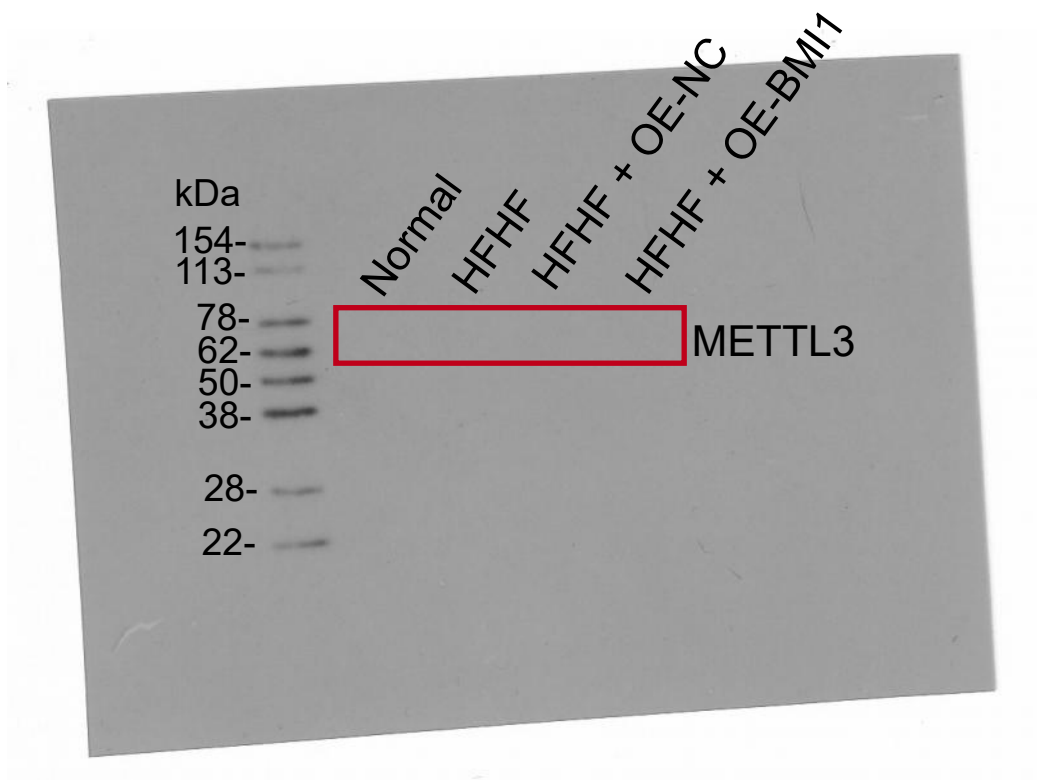
kDa
154-
113-
78-
62-
50-
38-
28-
22-
Normal
HFHF
HFHF + OE-NC
HFHF + OE-BMI1
METTL3

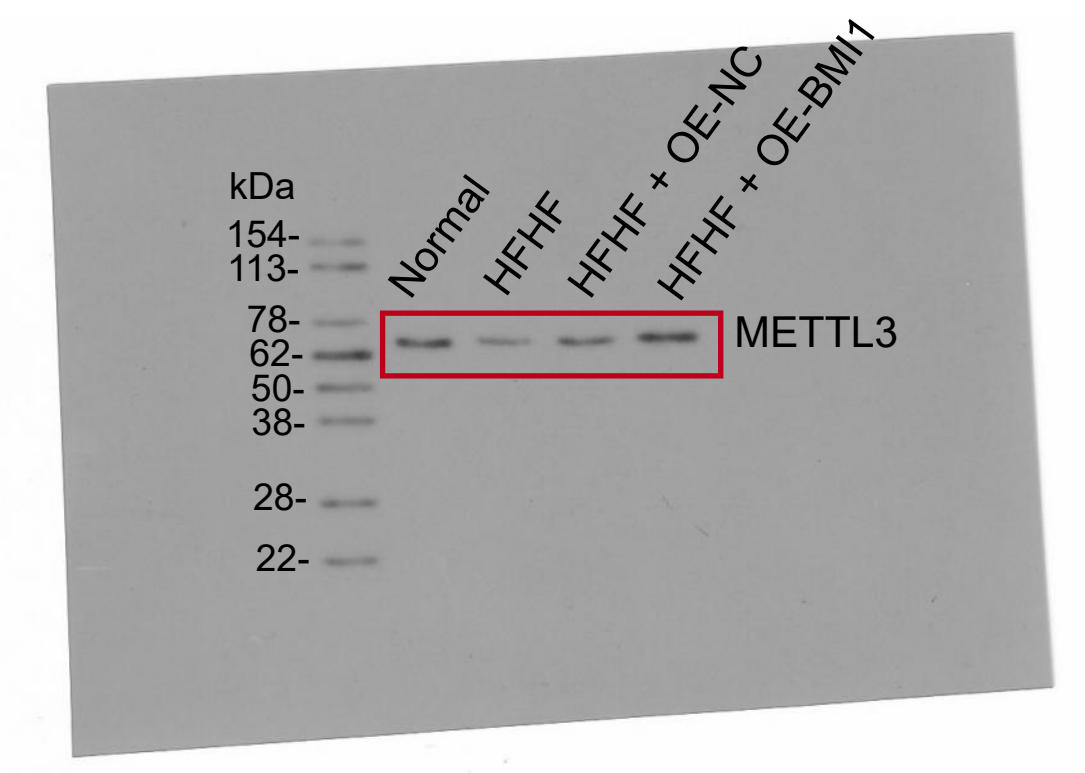
kDa
154-
113-
78-
62-
50-
38-
28-
22-
Normal
HFHF
HFHF + OE-NC
HFHF + OE-BMI1
METTL3

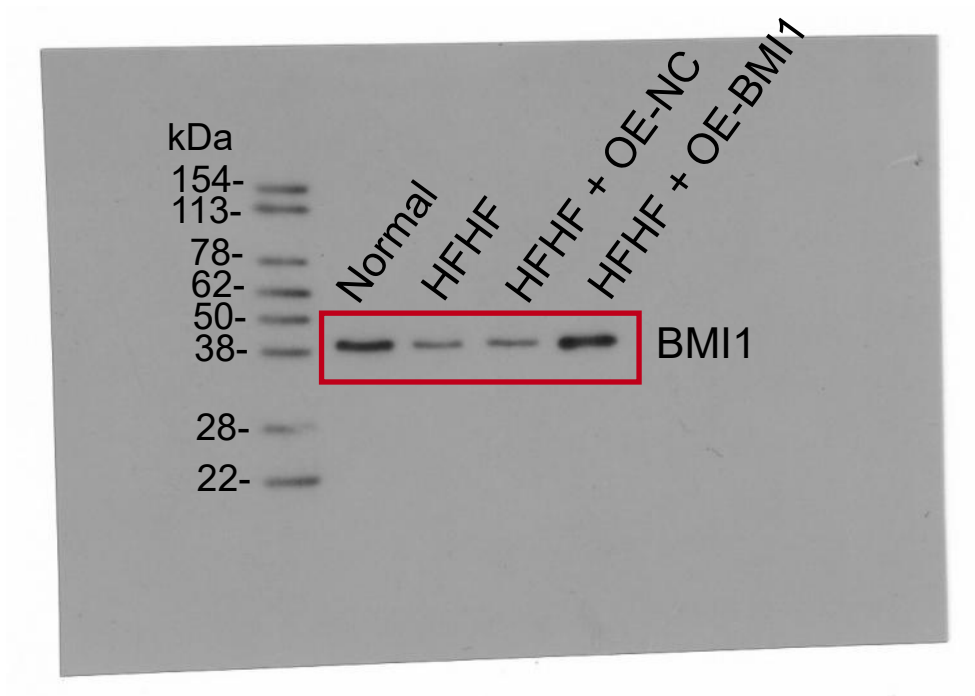
kDa
154-
113-
78-
62-
50-
38-
28-
22-
Normal
HFHF
HFHF + OE-NC
HFHF + OE-BMI1
BMI1

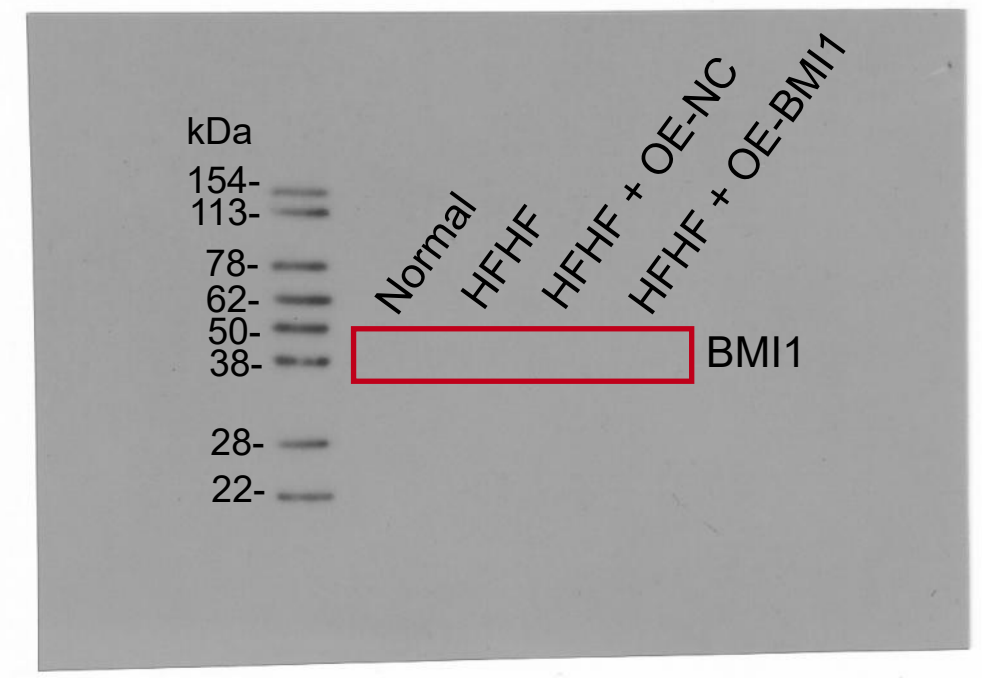
kDa
154-
113-
78-
62-
50-
38-
28-
22-
Normal
HFHF
HFHF + OE-NC
HFHF + OE-BMI1
BMI1

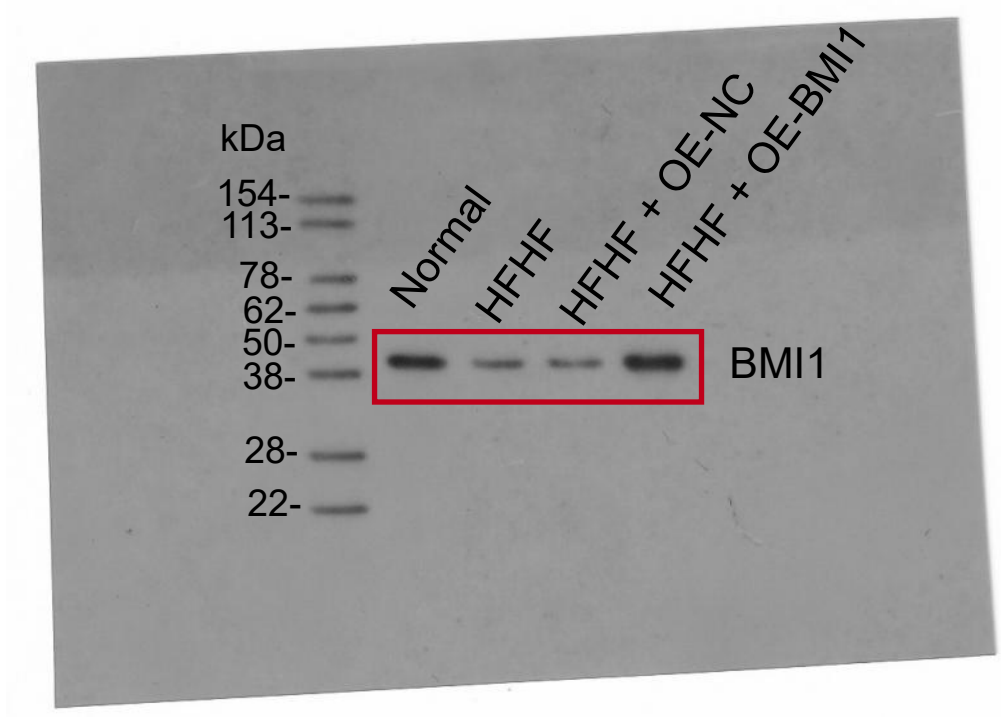
kDa
154-
113-
78-
62-
50-
38-
28-
22-
Normal
HFHF
HFHF + OE-NC
HFHF + OE-BMI1
BMI1

RAT #6

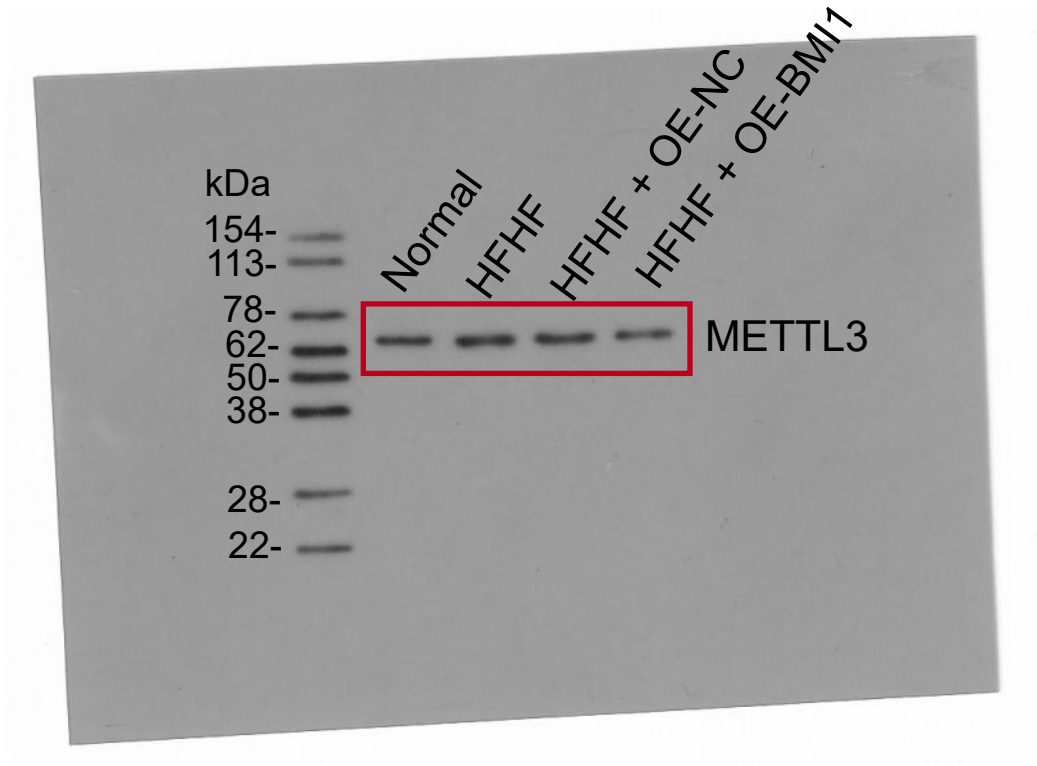
kDa
154-
113-
78-
62-
50-
38-
28-
22-
Normal
HFHF
HFHF + OE-NC
HFHF + OE-BMI1
METTL3

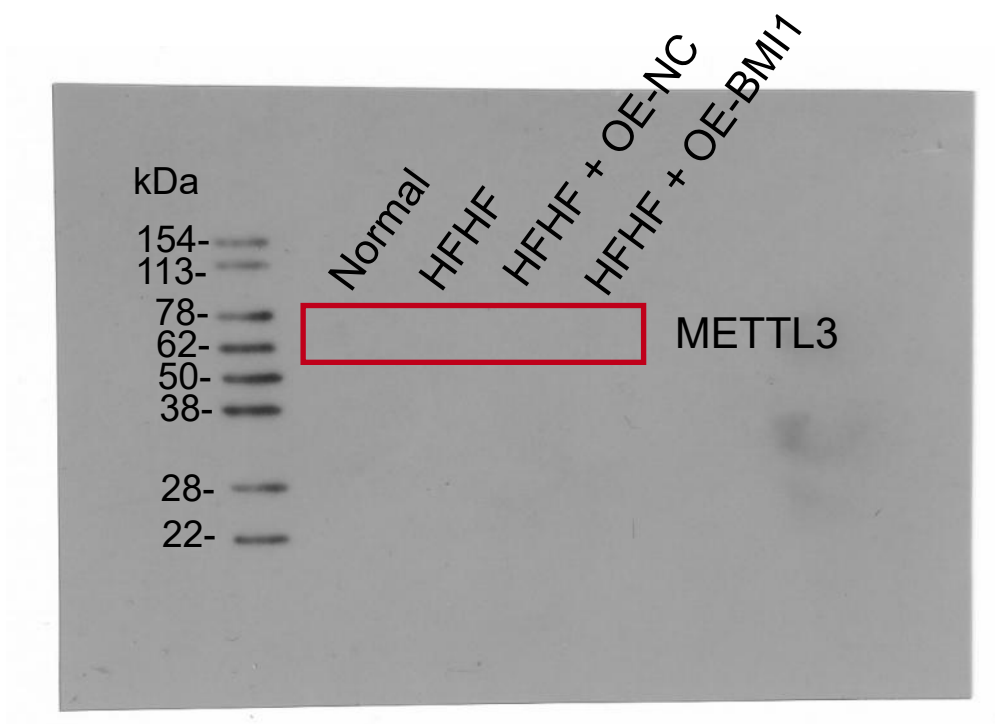
kDa
154-
113-
78-
62-
50-
38-
28-
22-
Normal
HFHF
HFHF + OE-NC
HFHF + OE-BMI1
METTL3

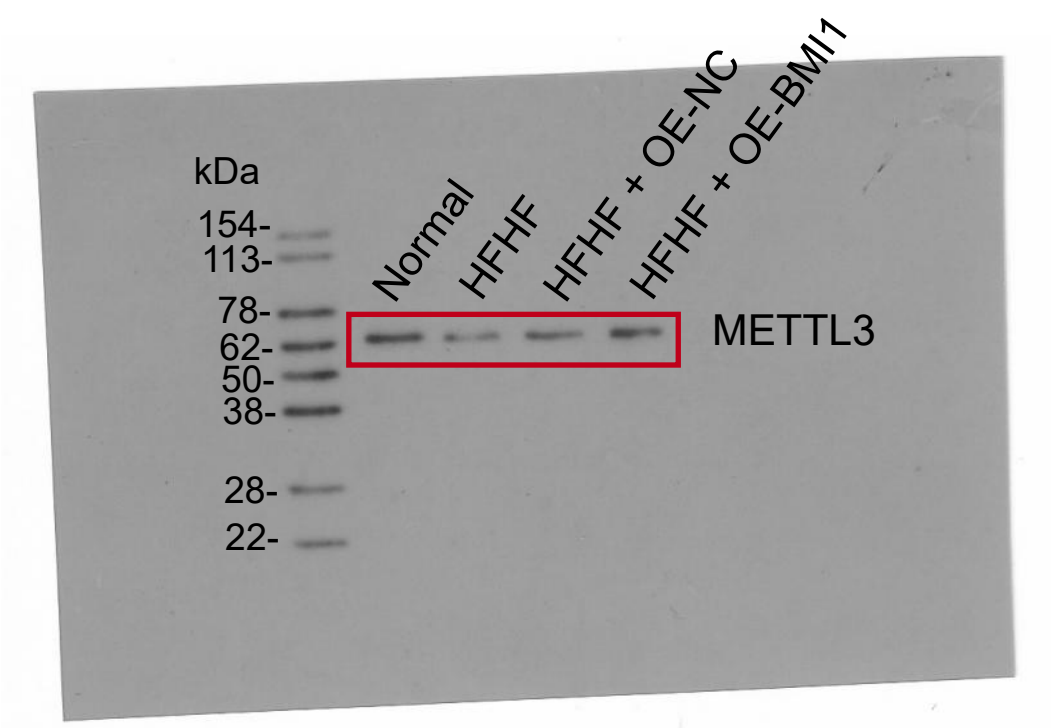
kDa
154-
113-
78-
62-
50-
38-
28-
22-
Normal
HFHF
HFHF + OE-NC
HFHF + OE-BMI1
METTL3

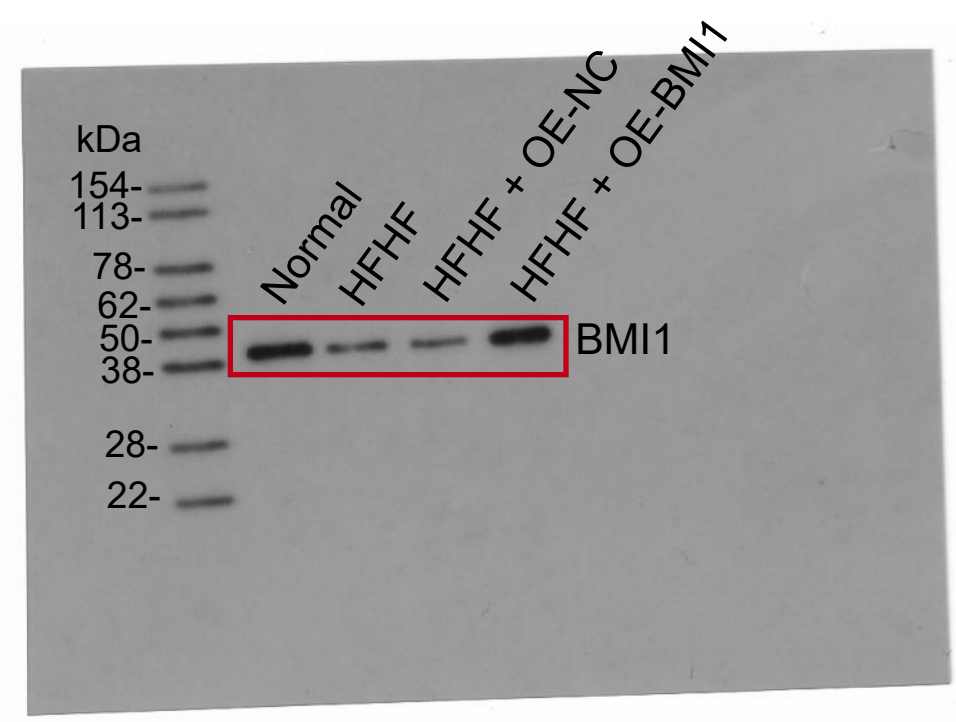
kDa
154-
113-
78-
62-
50-
38-
28-
22-
Normal
HFHF
HFHF + OE-NC
HFHF + OE-BMI1
BMI1

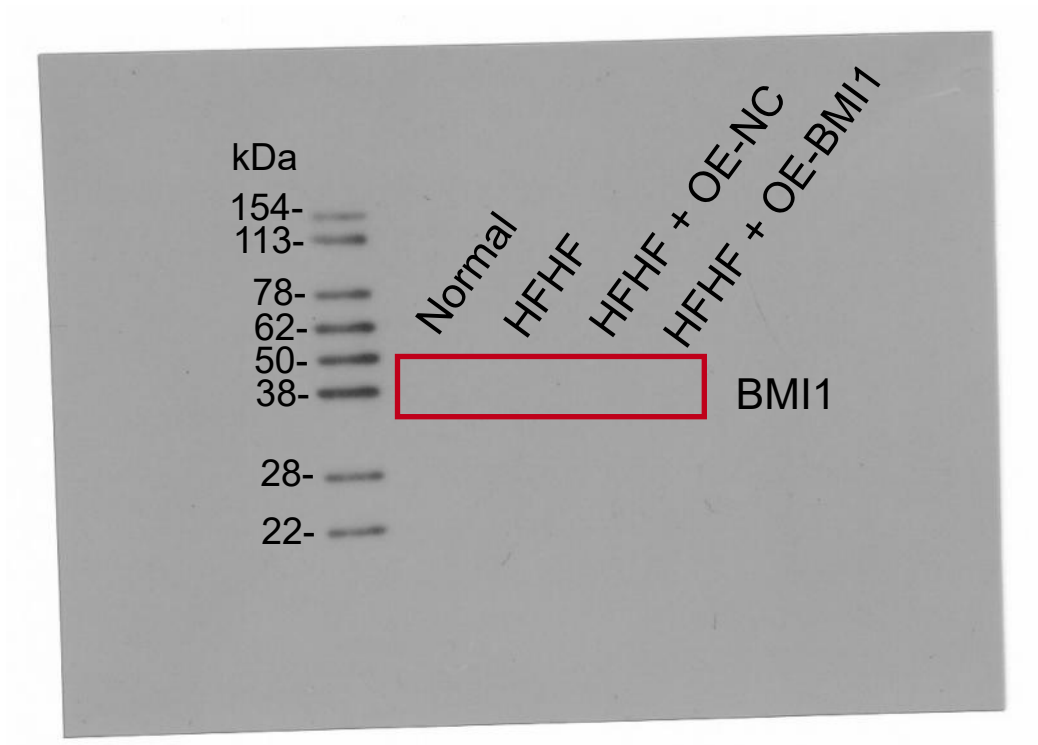
kDa
154-
113-
78-
62-
50-
38-
28-
22-
Normal
HFHF
HFHF + OE-NC
HFHF + OE-BMI1
BMI1

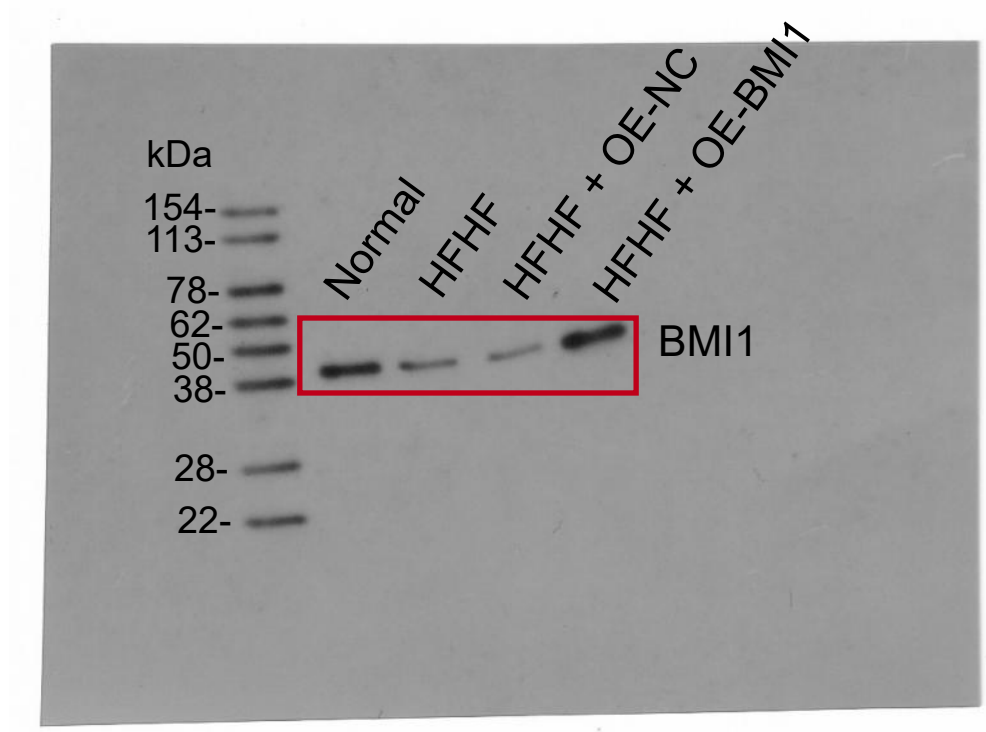
kDa
154-
113-
78-
62-
50-
38-
28-
22-
Normal
HFHF
HFHF + OE-NC
HFHF + OE-BMI1
BMI1

Fig 3A

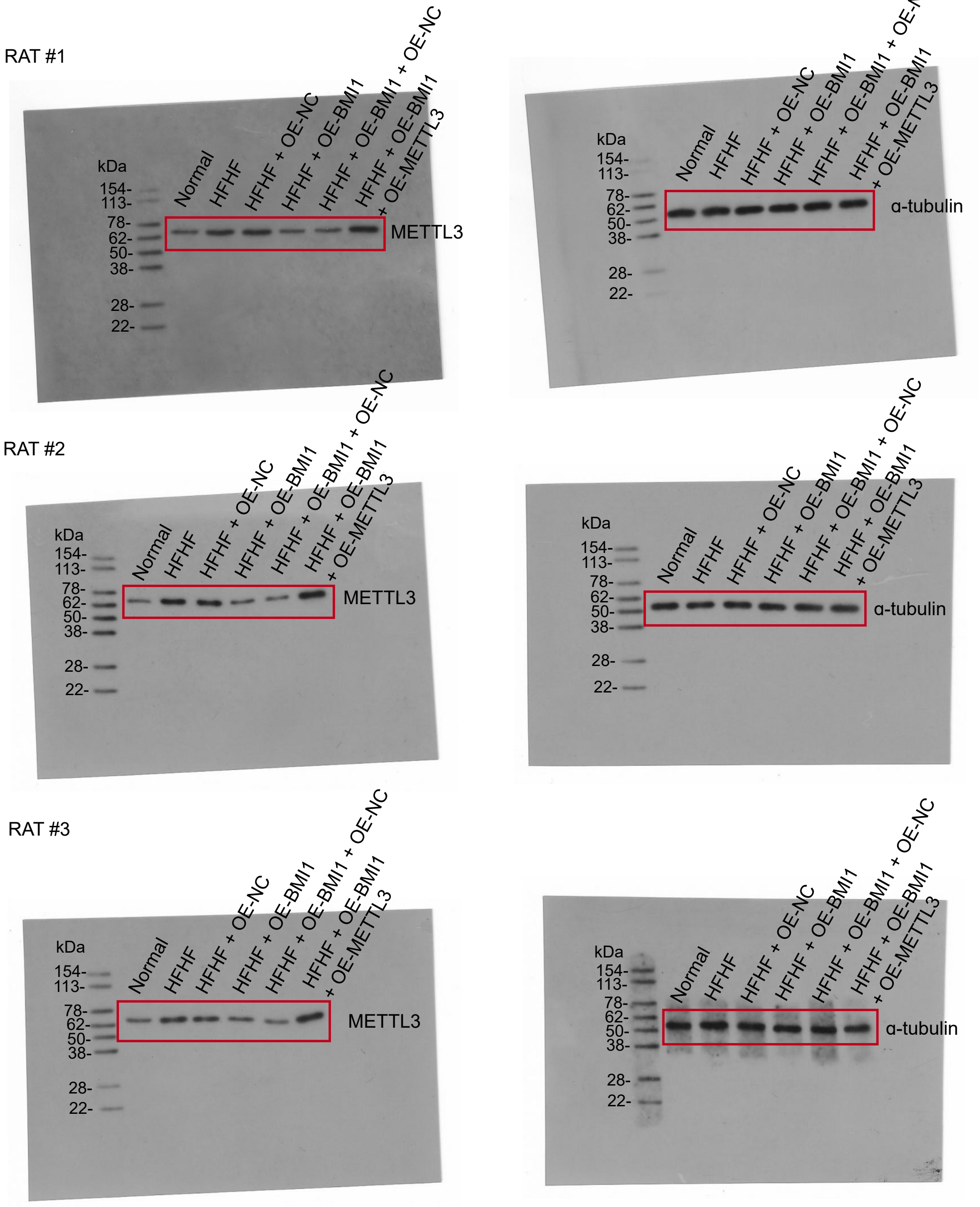
RAT #1
kDa
154-
113-
78-
62-
50-
38-
28-
22-
Normal
HFHF
HFHF + OE-NC
HFHF + OE-BMI1
HFHF + OE-BMI1 + OE-NC
HFHF + OE-BMI1 + OE-METTL3
METTL3
α-tubulin
RAT #2
METTL3
α-tubulin
RAT #3
METTL3
α-tubulin

RAT #4

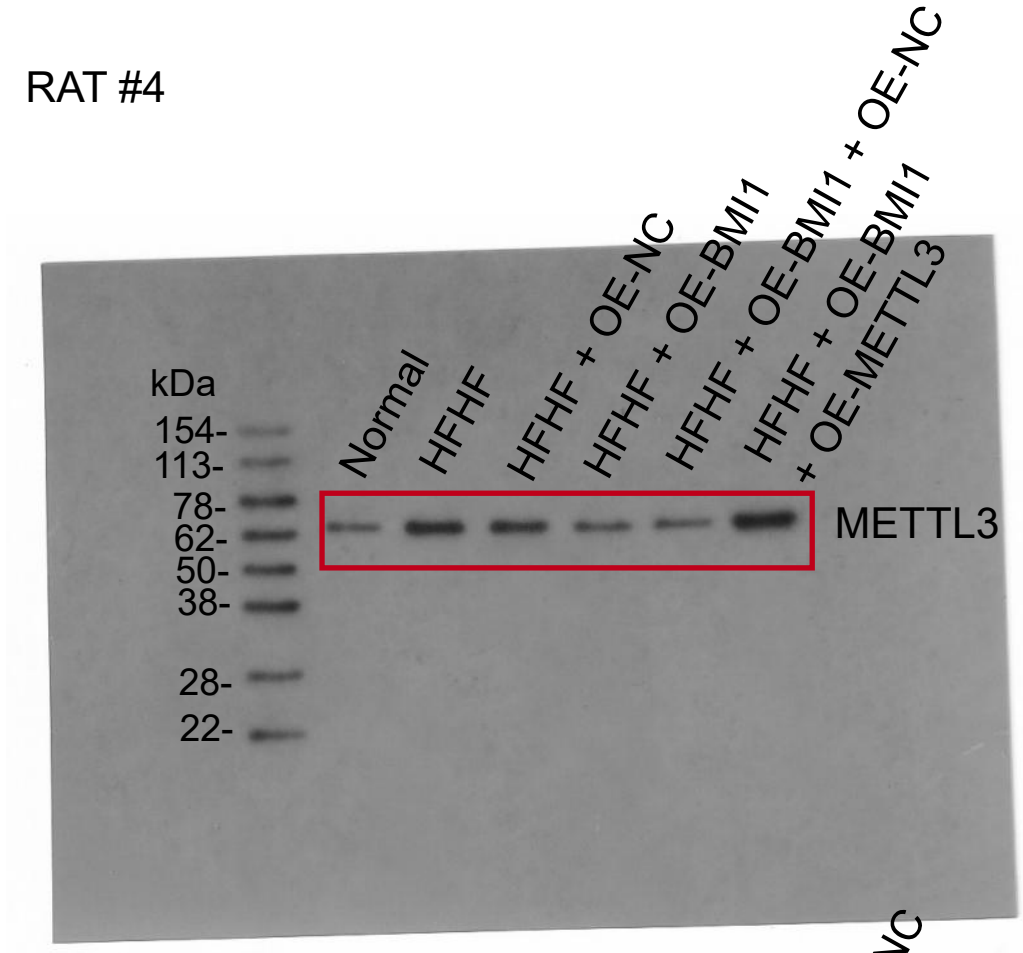

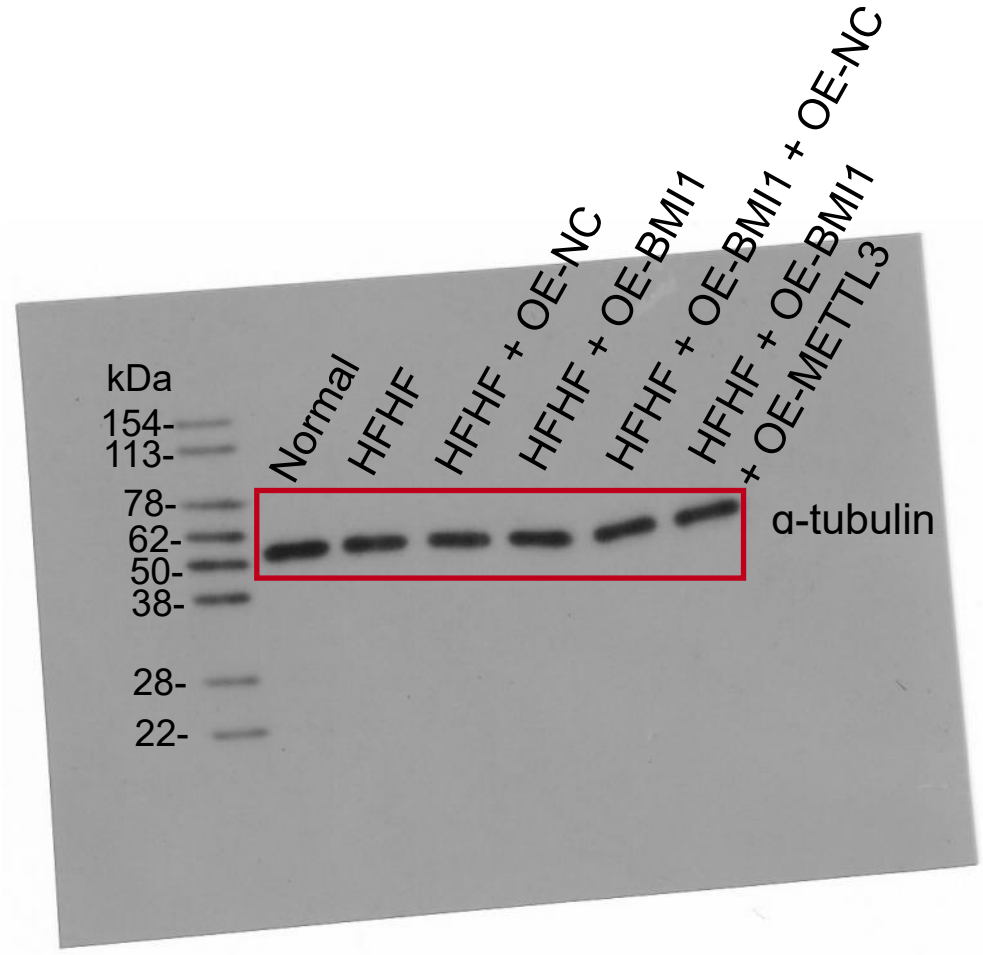

RAT #5

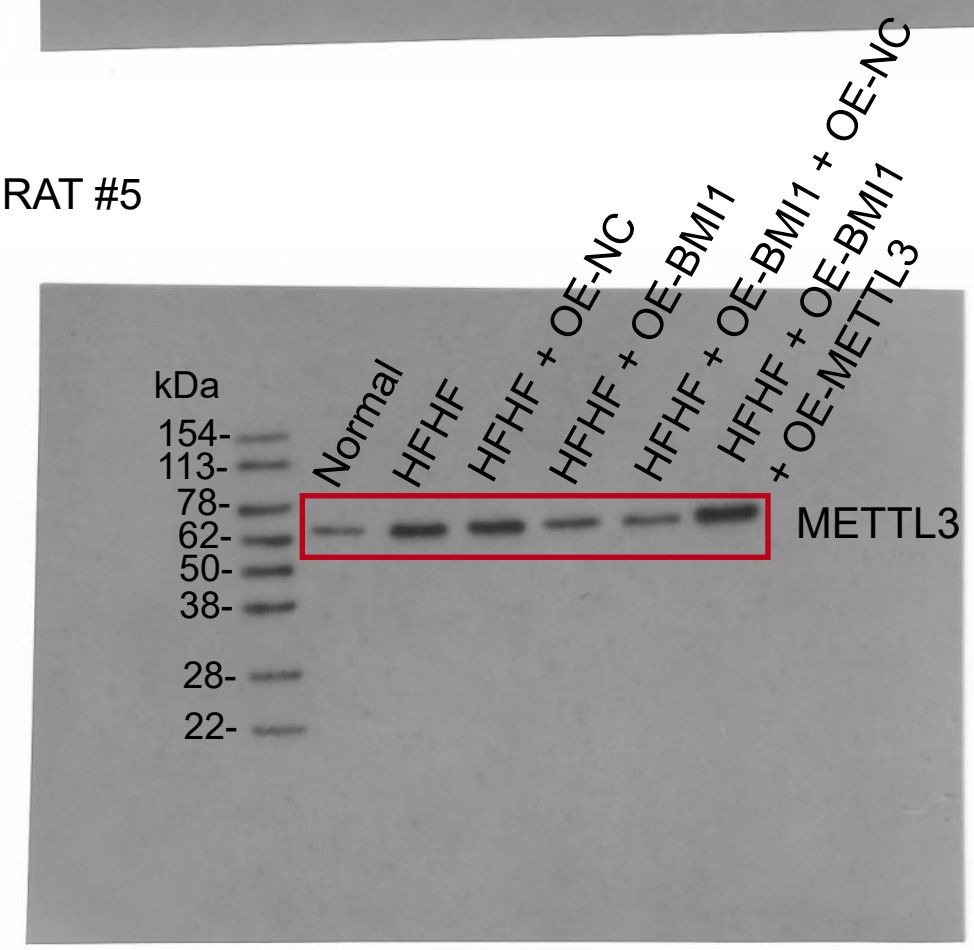

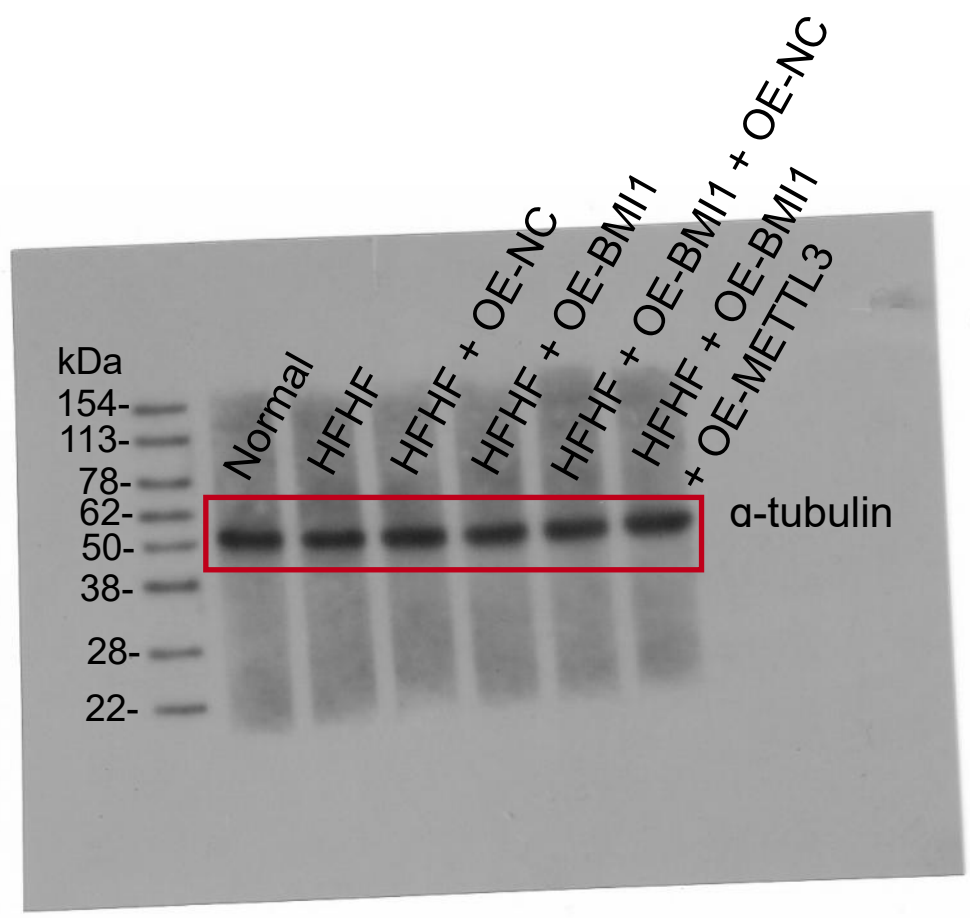

RAT #6

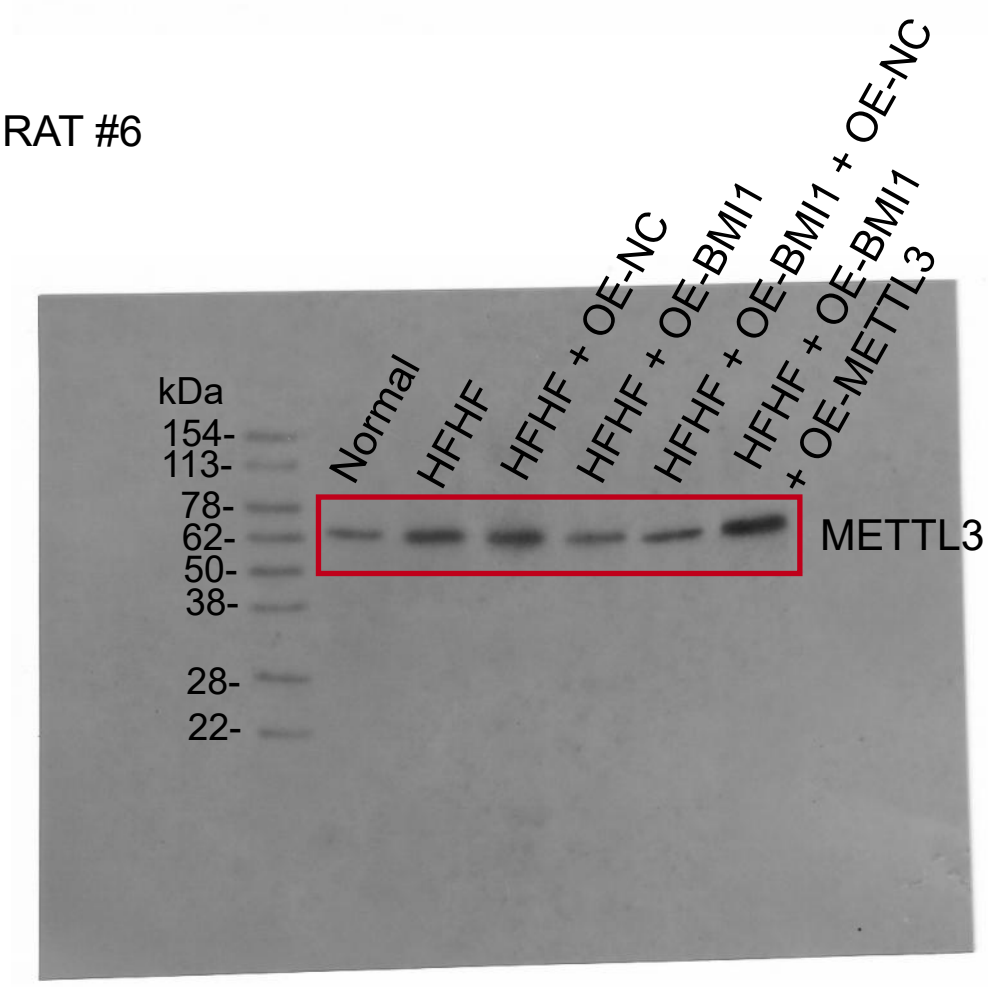

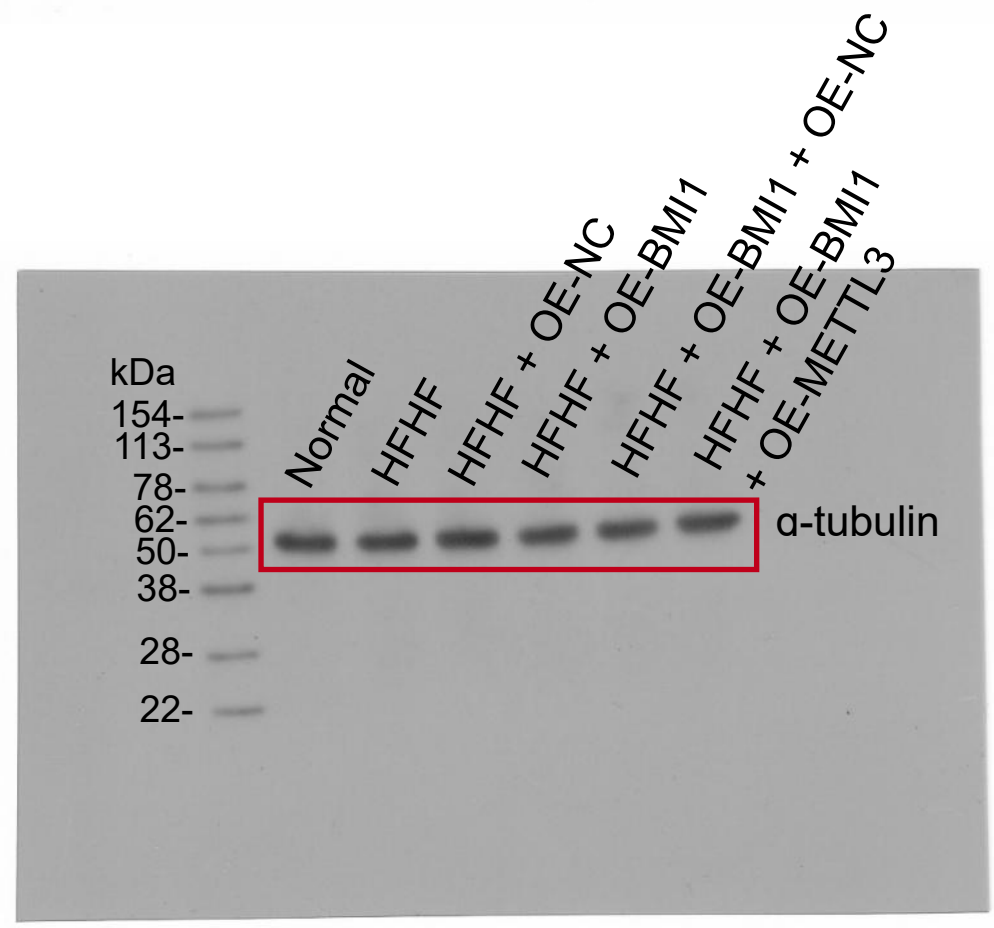

Fig 6A

RAT #1

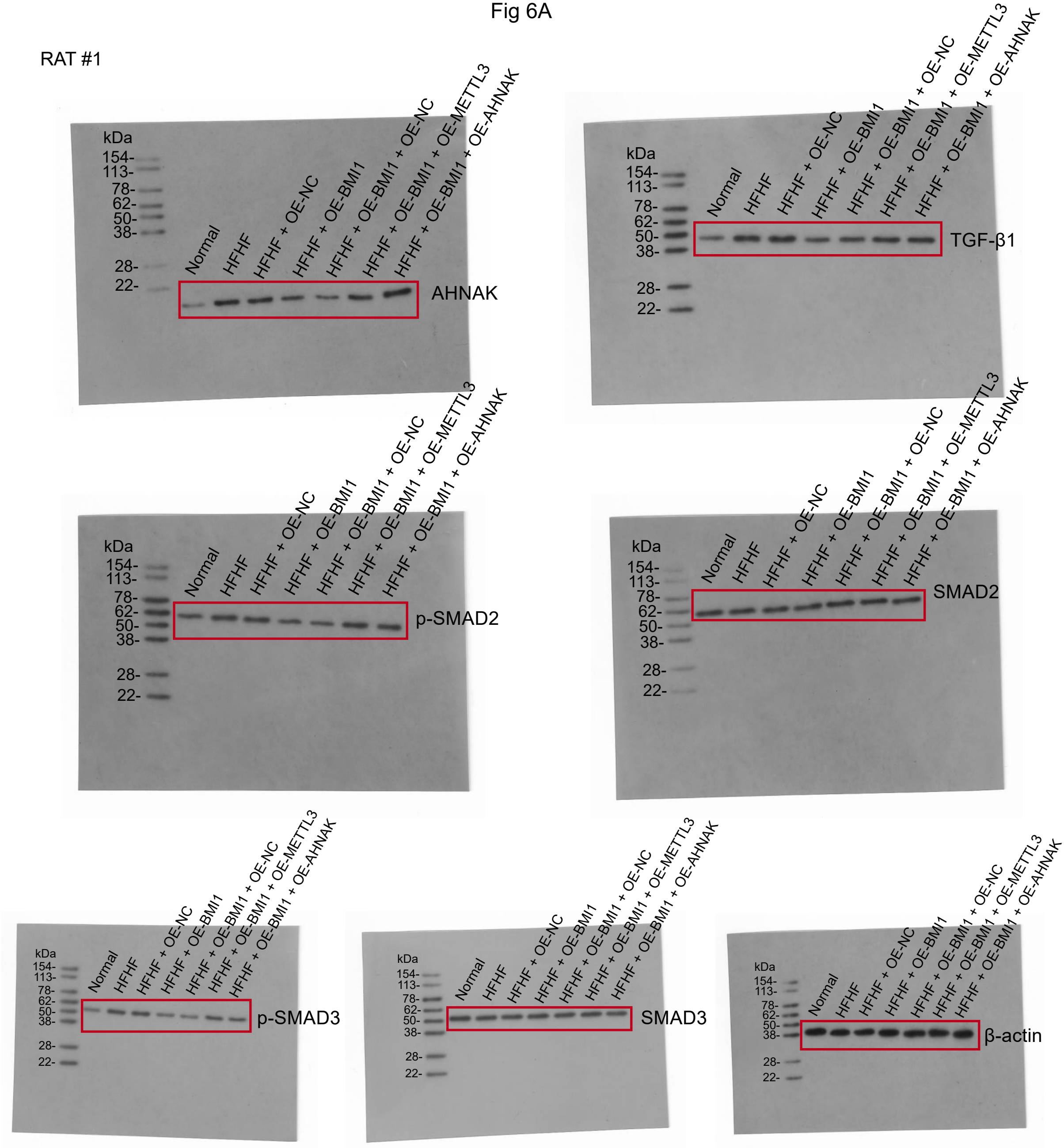
kDa
154-
113-
78-
62-
50-
38-
28-
22-
Normal
HFHF
HFHF + OE-NC
HFHF + OE-BMI1
HFHF + OE-BMI1 + OE-NC
HFHF + OE-BMI1 + OE-METTL3
HFHF + OE-BMI1 + OE-AHNAK
AHNAK
TGF-β1
p-SMAD2
SMAD2
p-SMAD3
SMAD3
β-actin

RAT #2

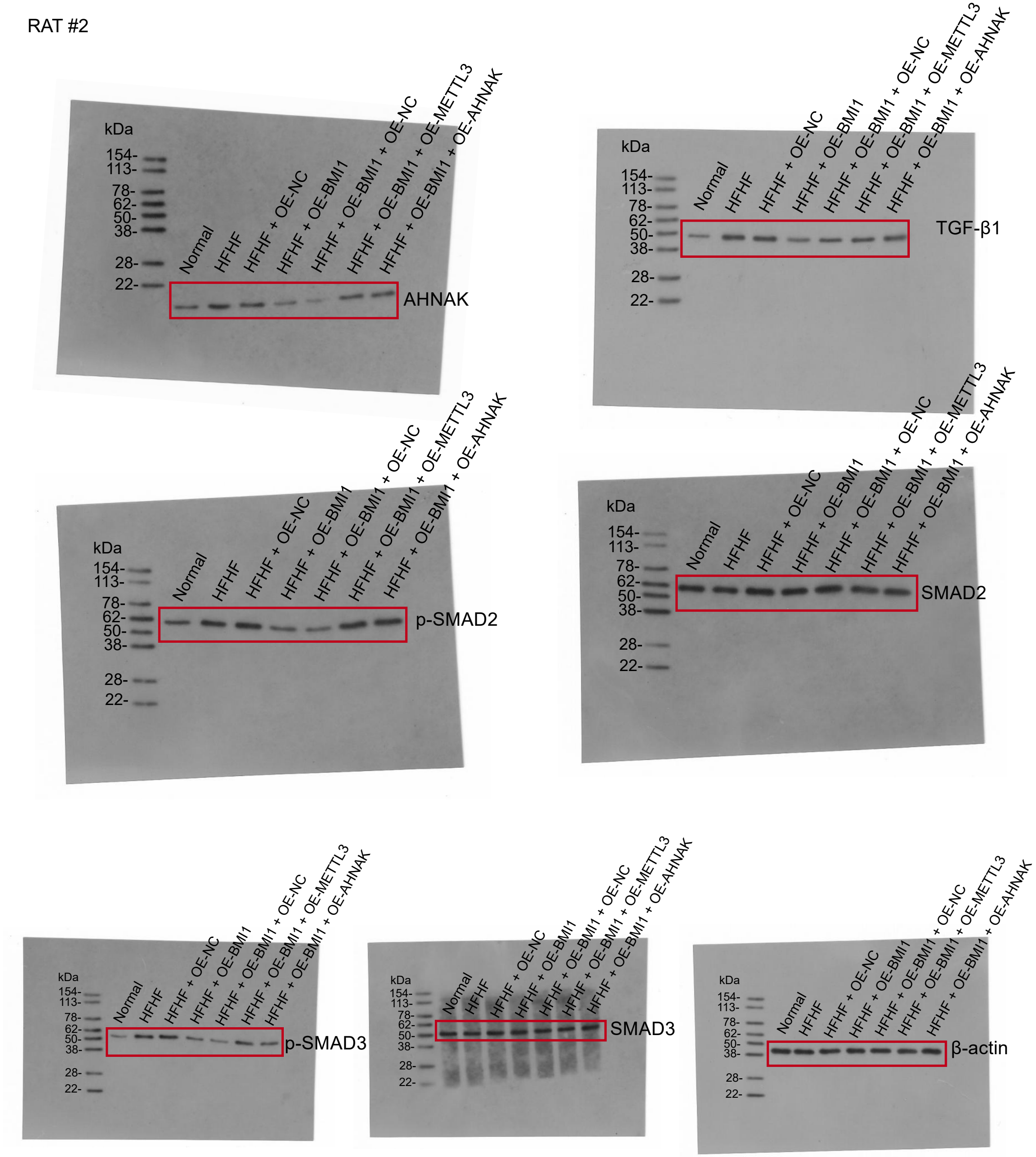

kDa
154-
113-
78-
62-
50-
38-
28-
22-
Normal
HFHF
HFHF + OE-NC
HFHF + OE-BMI1
HFHF + OE-BMI1 + OE-NC
HFHF + OE-BMI1 + OE-METTL3
HFHF + OE-BMI1 + OE-AHNAK
AHNAK
TGF-β1
p-SMAD2
SMAD2
p-SMAD3
SMAD3
β-actin

RAT #3

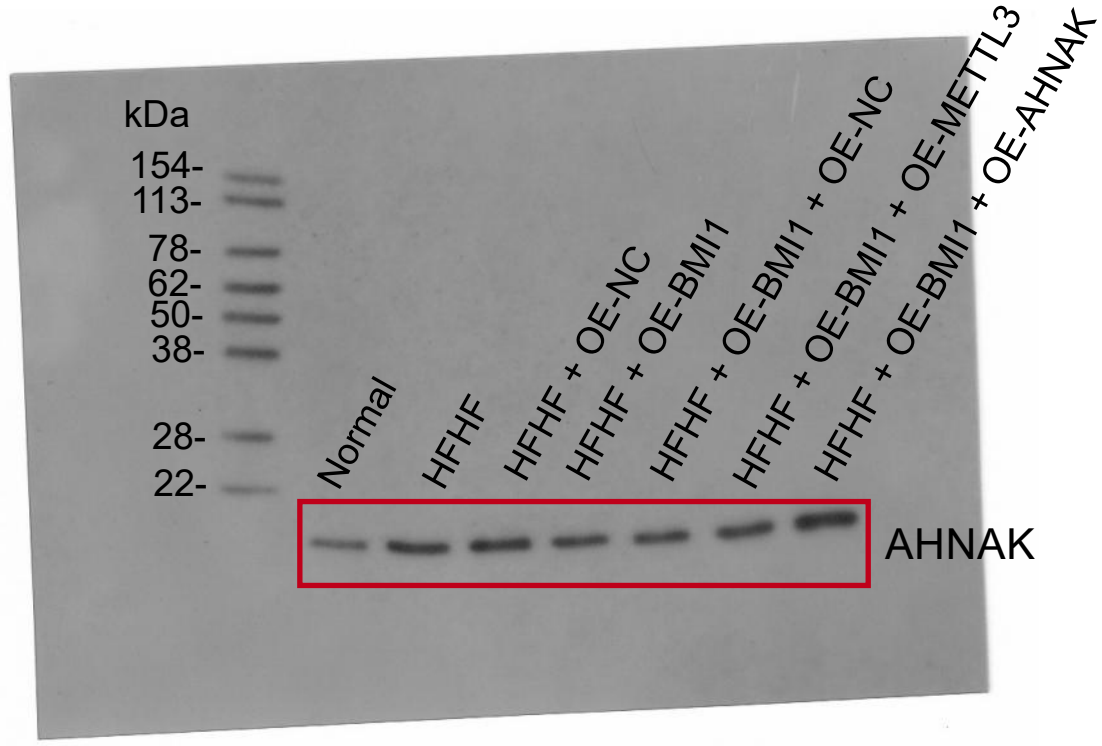
kDa
154-
113-
78-
62-
50-
38-
28-
22-
Normal
HFHF
HFHF + OE-NC
HFHF + OE-BMI1
HFHF + OE-BMI1 + OE-NC
HFHF + OE-BMI1 + OE-METTL3
HFHF + OE-BMI1 + OE-AHNAK
AHNAK

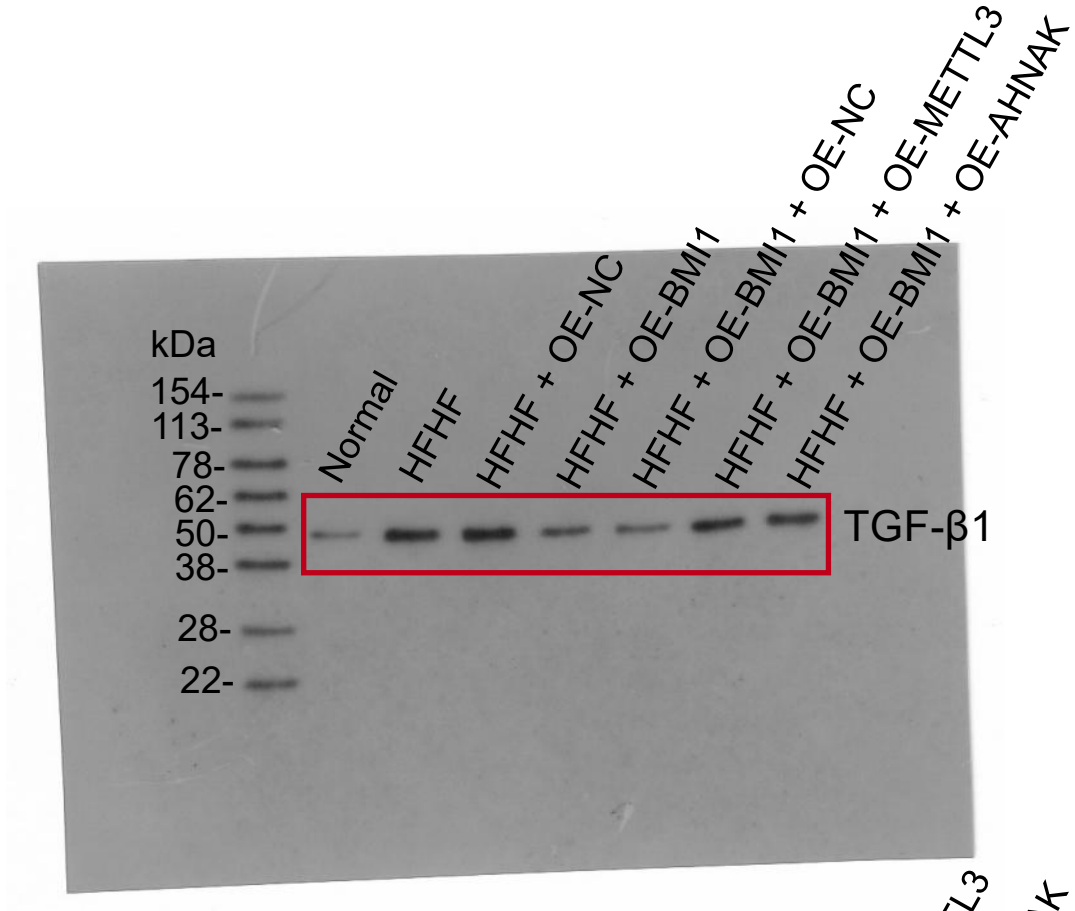
kDa
154-
113-
78-
62-
50-
38-
28-
22-
Normal
HFHF
HFHF + OE-NC
HFHF + OE-BMI1
HFHF + OE-BMI1 + OE-NC
HFHF + OE-BMI1 + OE-METTL3
HFHF + OE-BMI1 + OE-AHNAK
TGF-β1

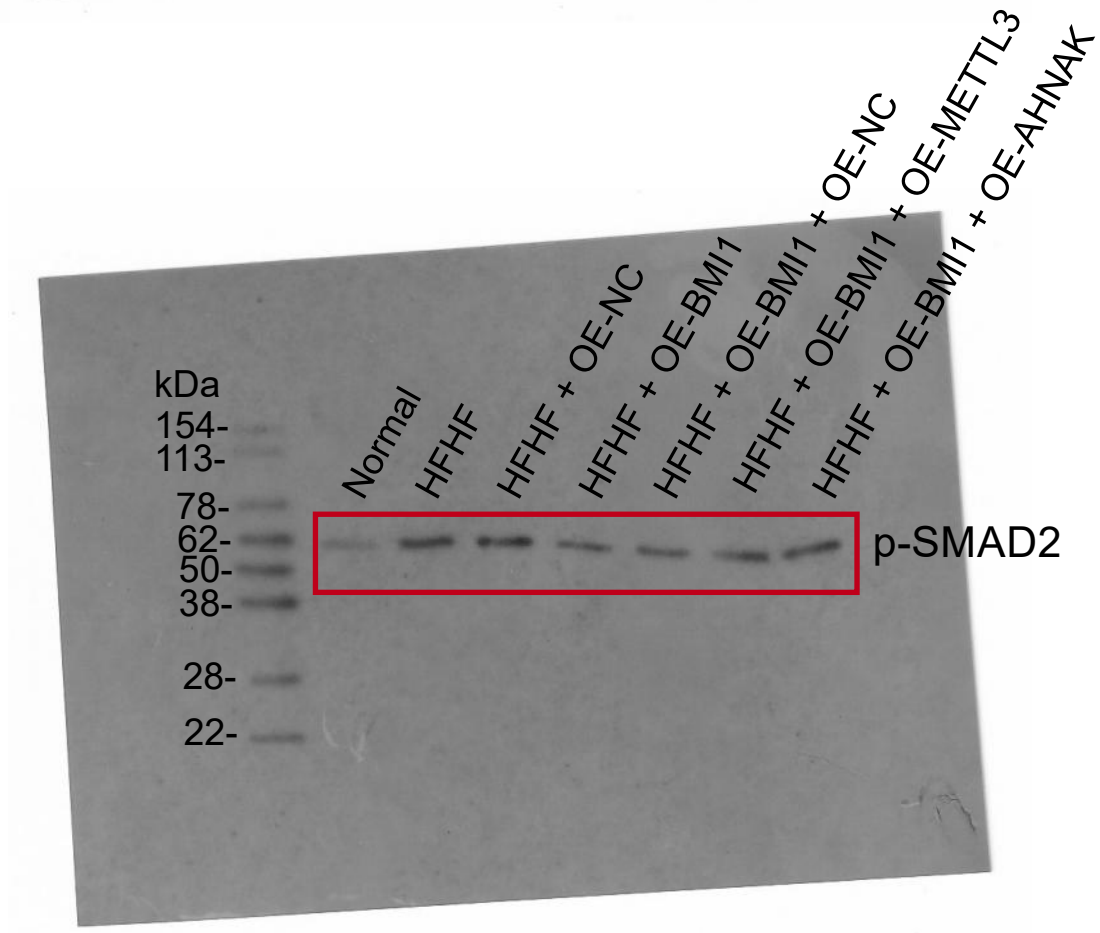
kDa
154-
113-
78-
62-
50-
38-
28-
22-
Normal
HFHF
HFHF + OE-NC
HFHF + OE-BMI1
HFHF + OE-BMI1 + OE-NC
HFHF + OE-BMI1 + OE-METTL3
HFHF + OE-BMI1 + OE-AHNAK
p-SMAD2

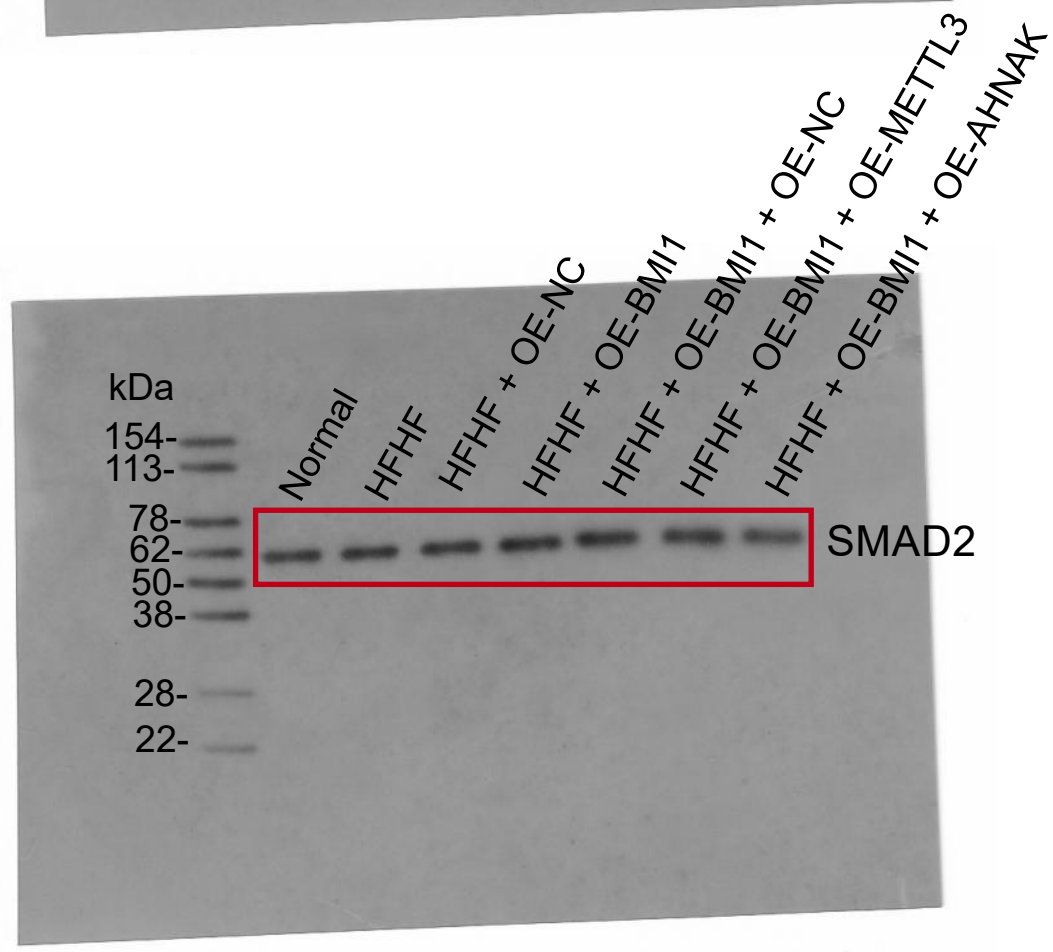
kDa
154-
113-
78-
62-
50-
38-
28-
22-
Normal
HFHF
HFHF + OE-NC
HFHF + OE-BMI1
HFHF + OE-BMI1 + OE-NC
HFHF + OE-BMI1 + OE-METTL3
HFHF + OE-BMI1 + OE-AHNAK
SMAD2

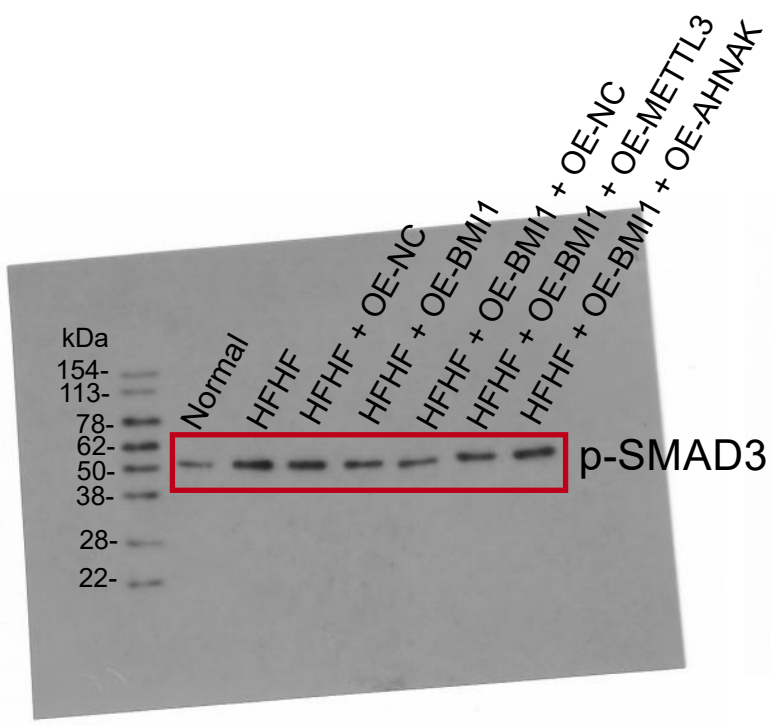
kDa
154-
113-
78-
62-
50-
38-
28-
22-
Normal
HFHF
HFHF + OE-NC
HFHF + OE-BMI1
HFHF + OE-BMI1 + OE-NC
HFHF + OE-BMI1 + OE-METTL3
HFHF + OE-BMI1 + OE-AHNAK
p-SMAD3

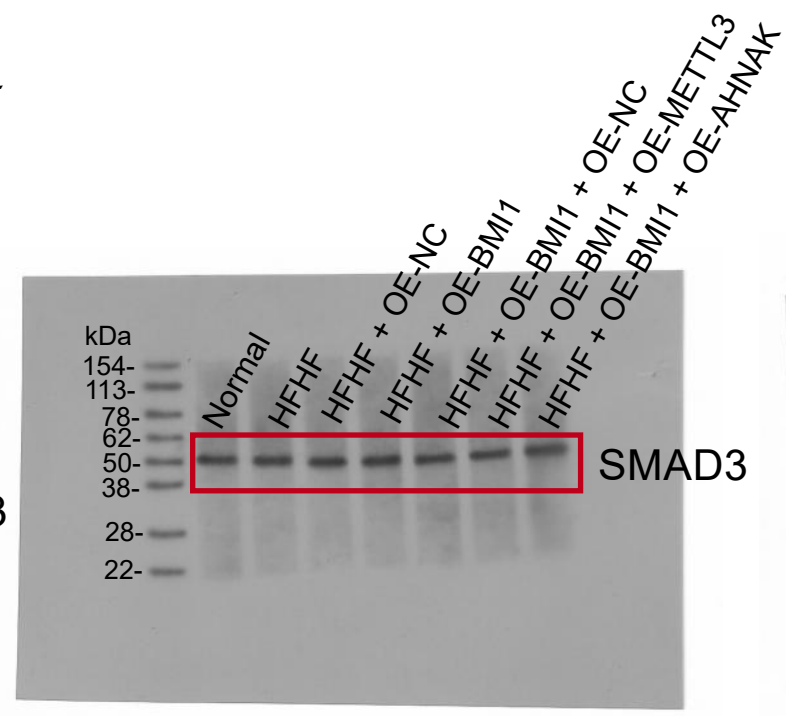
kDa
154-
113-
78-
62-
50-
38-
28-
22-
Normal
HFHF
HFHF + OE-NC
HFHF + OE-BMI1
HFHF + OE-BMI1 + OE-NC
HFHF + OE-BMI1 + OE-METTL3
HFHF + OE-BMI1 + OE-AHNAK
SMAD3

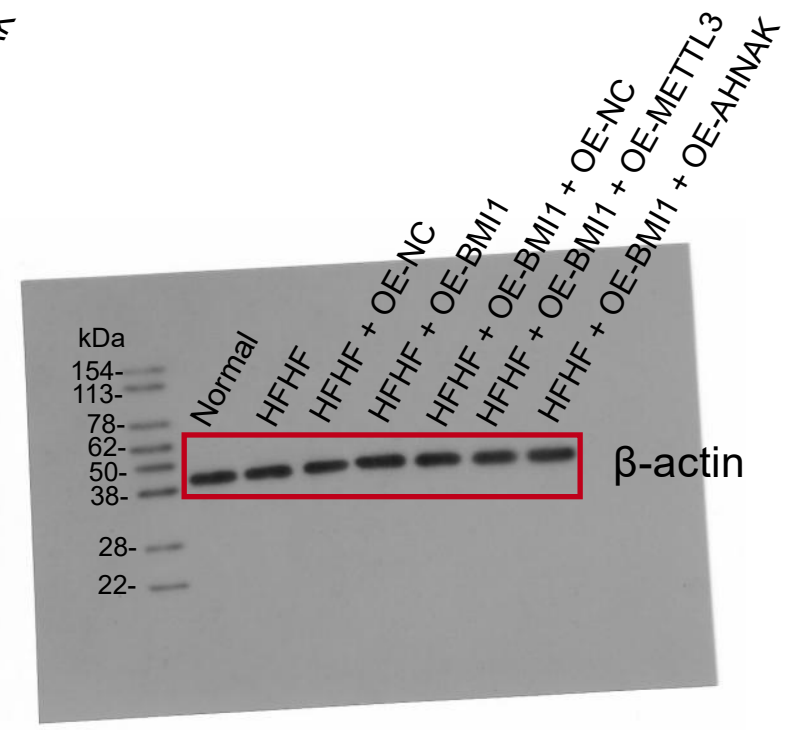
kDa
154-
113-
78-
62-
50-
38-
28-
22-
Normal
HFHF
HFHF + OE-NC
HFHF + OE-BMI1
HFHF + OE-BMI1 + OE-NC
HFHF + OE-BMI1 + OE-METTL3
HFHF + OE-BMI1 + OE-AHNAK
β-actin

RAT #4

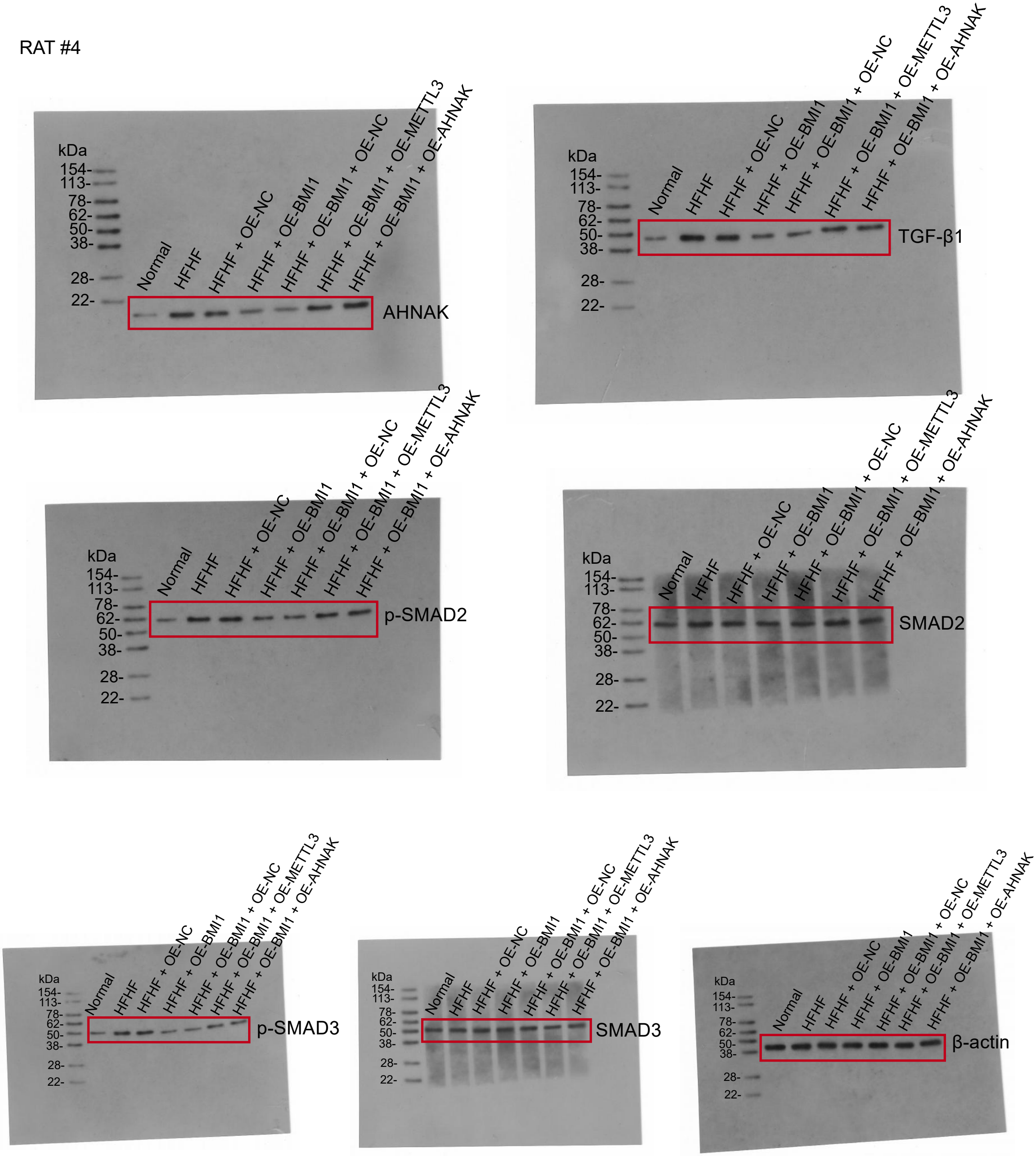
kDa
154-
113-
78-
62-
50-
38-
28-
22-
Normal
HFHF
HFHF + OE-NC
HFHF + OE-BMI1
HFHF + OE-BMI1 + OE-NC
HFHF + OE-BMI1 + OE-METTL3
HFHF + OE-BMI1 + OE-AHNAK
AHNAK
TGF-β1
p-SMAD2
SMAD2
p-SMAD3
SMAD3
β-actin

RAT #5

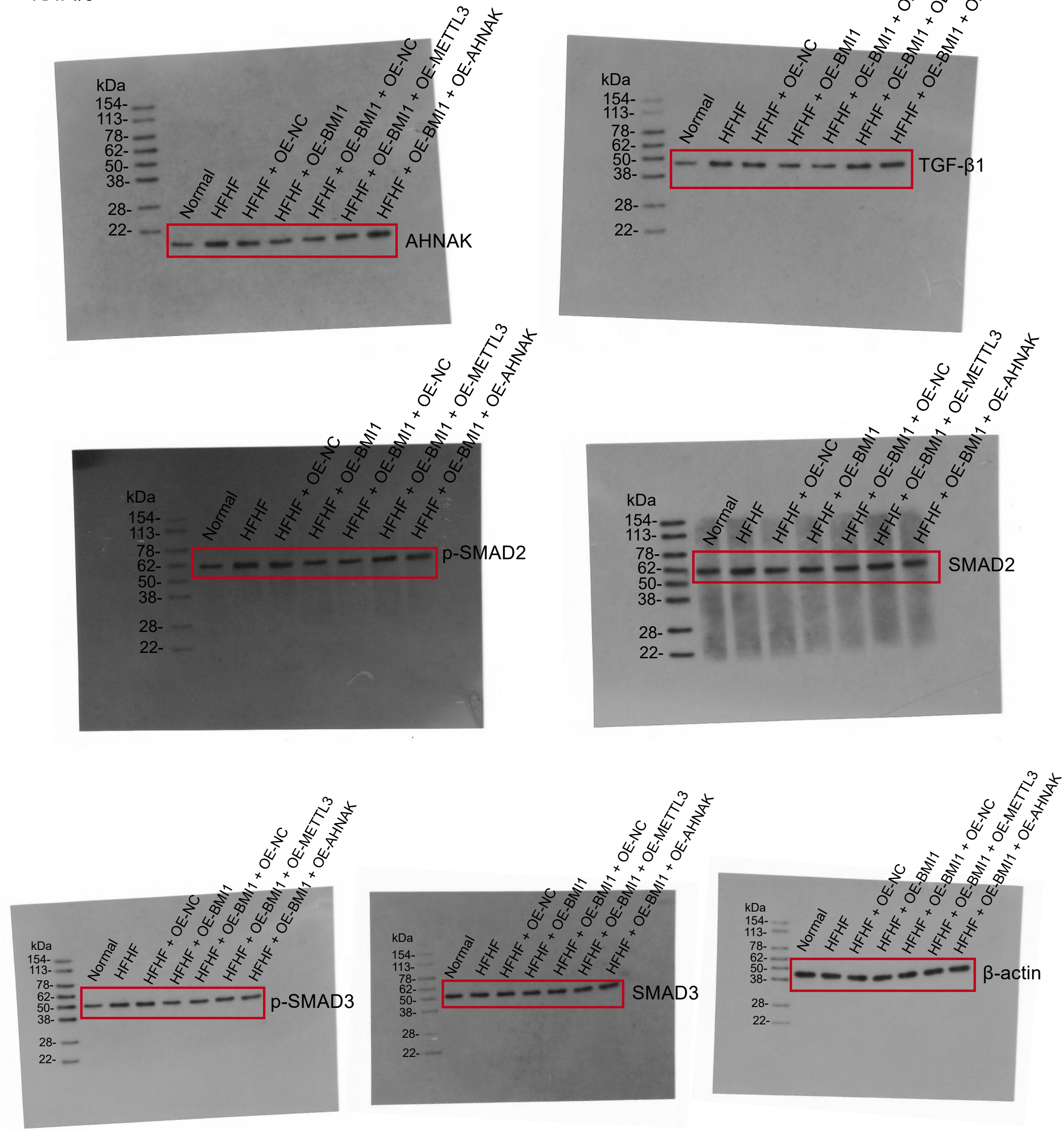
kDa
154-
113-
78-
62-
50-
38-
28-
22-
Normal
HFHF
HFHF + OE-NC
HFHF + OE-BMI1
HFHF + OE-BMI1 + OE-NC
HFHF + OE-BMI1 + OE-METTL3
HFHF + OE-BMI1 + OE-AHNAK
AHNAK
TGF-β1
p-SMAD2
SMAD2
p-SMAD3
SMAD3
β-actin

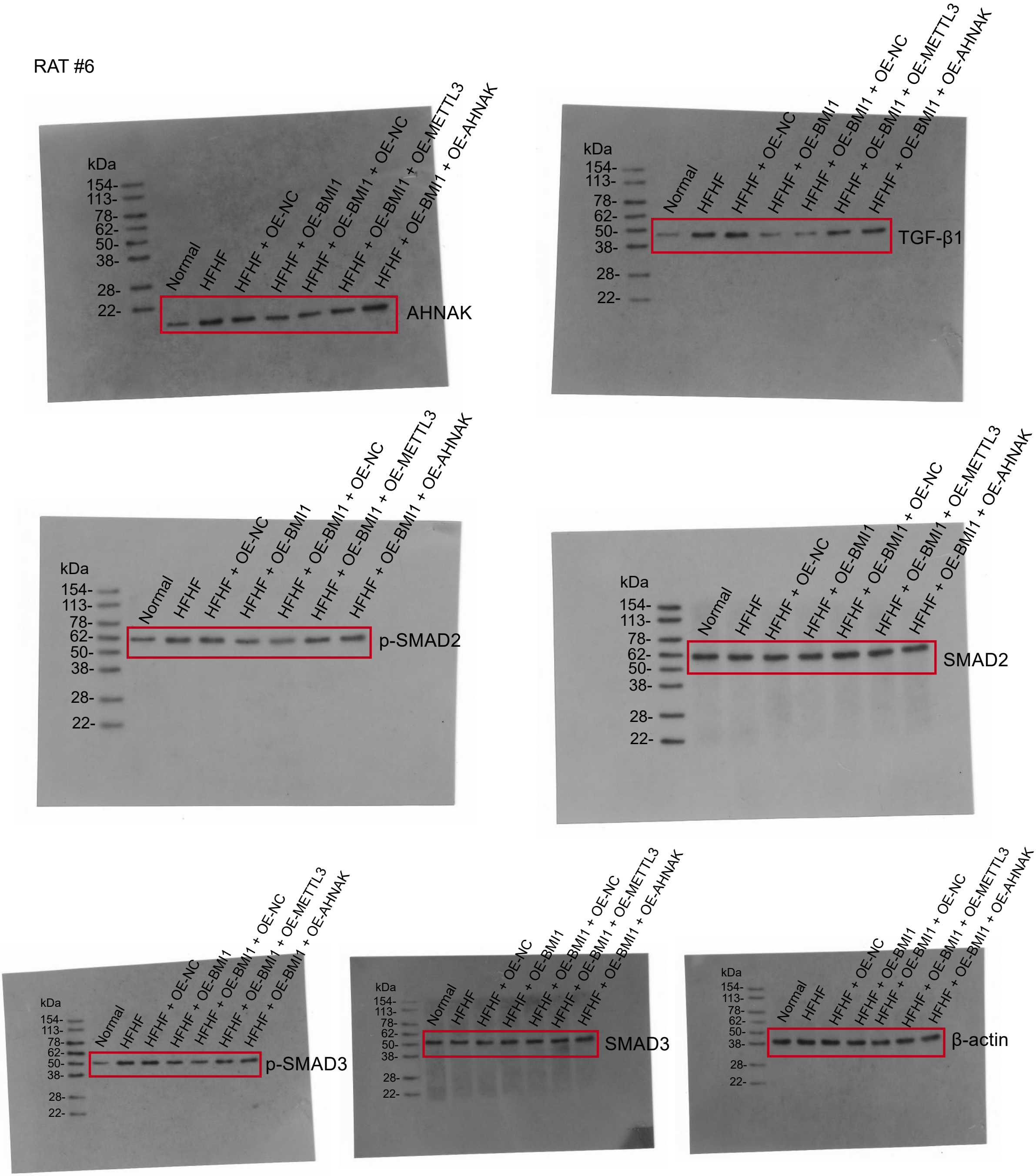
RAT #6
kDa
154-
113-
78-
62-
50-
38-
28-
22-
Normal
HFHF
HFHF + OE-NC
HFHF + OE-BMI1
HFHF + OE-BMI1 + OE-NC
HFHF + OE-BMI1 + OE-METTL3
HFHF + OE-BMI1 + OE-AHNAK
AHNAK
TGF-β1
p-SMAD2
SMAD2
p-SMAD3
SMAD3
β-actin
