# Supplementary material for: BMI1 Overexpression Alleviates Metabolic Syndrome‐Related Liver Injury by Inhibiting the Activation of the METTL3/AHNAK Axis
Source: Kaohsiung J Med Sci. 2026 Oct 26:e70308. Online ahead of print. doi: 10.1002/kjm2.70308 (PMC13697295; doi:10.1002/kjm2.70308)
Supplement: Supplementary file 2 [file KJM2-9999-e70308-s001.docx]

**Figure S1**

**Figure S1** Analysis of the subcellular localization of AHNAK. (A) The expression of AHNAK mRNA in the cytoplasm and nucleus following METTL3 overexpression was examined using FISH. (B) AHNAK protein expression in the nucleus and cytoplasm following METTL3 overexpression was examined using Western blot analysis. (C) Expression of AHNAK in liver samples from the Human Protein Atlas project. Data are presented as means ± SEM (n = 5), and statistical significance was assessed using an unpaired t-test.
